# Safety and immunogenicity of the Vi-DT typhoid conjugate vaccine in healthy volunteers in Nepal: an observer-blind, active-controlled, randomised, non-inferiority, phase 3 trial

**DOI:** 10.1016/S1473-3099(21)00455-2

**Published:** 2022-04

**Authors:** Ganesh Kumar Rai, Tarun Saluja, Shipra Chaudhary, Dipesh Tamrakar, Piush Kanodia, Bishnu Rath Giri, Rajeev Shrestha, Surendra Uranw, Deok Ryun Kim, Jae Seung Yang, Il-Yeon Park, Seung-Eun Kyung, Sridhar Vemula, Jagadeesh Reddy E, Bomi Kim, Birendra Prasad Gupta, Sue Kyoung Jo, Ji Hwa Ryu, Ho Keun Park, Jong Hoon Shin, Yoonyeong Lee, Hun Kim, Jerome H Kim, Zenaida Reynoso Mojares, T Anh Wartel, Sushant Sahastrabuddhe

**Affiliations:** a Department of Pediatrics, Kanti Children's Hospital, Kathmandu, Nepal; b International Vaccine Institute, SNU Research Park, Seoul, South Korea; c Department of Pediatrics and Medicine, B P Koirala Institute of Health Sciences, Dharan, Nepal; d Department of Community Medicine and Pharmacology, Dhulikhel Hospital, Kathmandu University Hospital, Dhulikhel, Nepal; e Department of Pediatrics, Nepalgunj Medical College, Nepalgunj, Nepal; f SK Bioscience, Seoul, South Korea

## Abstract

**Background:**

Typhoid fever is an endemic disease in many low-income and middle-income countries. The 2018 WHO position paper recommends that countries should consider typhoid vaccination in high-risk groups and for outbreak control. To address the typhoid vaccine supply and demand gap, a typhoid Vi polysaccharide-diphtheria toxoid (Vi-DT) conjugate vaccine development effort was undertaken to achieve WHO prequalification and contribute to the global supply of typhoid conjugate vaccine. The main aim of this study was to show immune non-inferiority of the Vi-DT vaccine compared with the WHO prequalified Vi polysaccharide-tetanus toxoid (Vi-TT) conjugate vaccine (Typbar TCV; Bharat Biotech India, Hyderabad, India) in participants of various ages from an endemic country.

**Methods:**

We did an observer-blind, active-controlled, randomised, non-inferiority, phase 3 trial at four hospitals in Kathmandu, Dhulikhel, Dharan, and Nepalgunj in Nepal. Eligible participants were healthy individuals aged 6 months to 45 years for whom informed consent was obtained, were willing to follow the study procedures and were available for the duration of the study. Patients with an acute or chronic illness that could interfere with interpretation of the study endpoints, or who were involved in any other clinical trial were excluded. Participants were randomly assigned (1:1:1:1) by block randomisation (block size of four and eight), stratified by age (6 months to <2 years, 2 years to <18 years, and 18 years to 45 years), into one of four groups (A–D). Participants in groups A–C received a single dose (25 μg; 0·5 mL) of Vi-DT test vaccine via intramuscular injection from one of three good manufacturing practice lots (group A received lot 1, group B received lot 2, and group C received lot 3), and those in group D received a single dose (25 μg; 0·5 mL) of the Vi-TT vaccine via intramuscular injection. All participants, site staff (except for those who administered the study vaccines), and those assessing the outcomes were masked to group assignment. The co-primary endpoints were: (1) non-inferiority of immunogenicity of the Vi-DT vaccine (pooled groups A–C) versus the Vi-TT vaccine (group D), measured by the anti-Vi IgG seroconversion rate at 4 weeks after vaccination; and (2) the lot-to-lot consistency of the Vi-DT vaccine, measured by immune equivalence of the anti-Vi IgG geometric mean titre (GMT) at 4 weeks after receipt of the three Vi-DT vaccine lots (lot 1 *vs* lot 2, lot 1 *vs* lot 3, and lot 2 *vs* lot 3). Non-inferiority of the Vi-DT vaccine compared with the Vi-TT vaccine was shown if the lower limit of the 97·5% CI for the difference between the seroconversion rates in Vi-DT vaccine groups A–C combined versus Vi-TT vaccine group D was above the predefined non-inferiority margin of −10%. Lot-to-lot immune equivalence was shown if the upper and lower bounds of the two-sided 99·17% CI around the GMT ratio for each pairwise lot-to-lot comparison was between 0·67 and 1·50, which is the predefined equivalence margin recommended by WHO. The co-primary immunogenicity endpoints were assessed in all randomised participants who had received their assigned vaccine and had completed at least one post-baseline immunogenicity assessment. Safety was descriptively summarised by group and age strata, and was assessed in all participants who had received one dose of the investigational vaccine. The trial is registered with ClinicalTrials.gov, NCT03933098.

**Findings:**

Between Nov 20, 2019, and March 10, 2020, 1854 individuals were screened, of whom 1800 were enrolled and randomly assigned to groups A–D (450 participants in each group). 1786 (99·2%; 443 in group A, 450 in group B, 447 in group C, and 446 in group D) were included in the immunogenicity assessments at 4 weeks post vaccination, and all 1800 participants were included in the safety analysis. In the immunogenicity analysis, the anti-Vi-IgG seroconversion rate in all age strata was 99·33% (97·5% CI 98·61 to 99·68; 1331 of 1340 participants) in Vi-DT vaccine groups A–C and 98·88% (97·10 to 99·57; 441 of 446) in Vi-TT vaccine group D. The difference in seroconversion rates between Vi-DT vaccine groups A–C combined versus Vi-TT group D was 0·47% (97·5% CI −0·68 to 1·61), indicating non-inferiority of the Vi-DT vaccine. Anti-Vi-IgG GMT ratios at 4 weeks post-vaccination were 1·02 (99·17% CI 0·85 to 1·22) for lot 1 versus lot 2, 1·02 (0·85 to 1·23) for lot 1 versus lot 3, and 1·01 (0·84 to 1·21) for lot 2 versus lot 3, indicating lot-to-lot equivalence according to the predefined, WHO-recommended equivalence margin. The proportion of participants reporting adverse events was similar between Vi-DT vaccine groups A–C and Vi-TT vaccine group D; 260 (19·3%) of 1350 participants in Vi-DT vaccine groups A–C and 115 (25·6%) of 450 in Vi-TT vaccine group D reported solicited adverse events within 7 days after vaccination, and 208 (15·4%) in Vi-DT vaccine groups A–C and 76 (16·9%) in Vi-TT vaccine group D reported unsolicited adverse events within 4 weeks after vaccination. Seven serious adverse events (four [0·3%] participants in Vi-DT vaccine groups A–C and three [0·7%] in Vi-TT vaccine group D), including one death in the Vi-TT vaccine group, were reported during the 24-week follow-up period, none of which were considered related to the investigational product.

**Interpretation:**

When administered as a single dose, the Vi-DT test vaccine was safe, immunogenic, and non-inferior to the Vi-TT vaccine at 4 weeks post vaccination. Equivalent immunogenicity of the three lots of Vi-DT vaccine was also shown, supporting the manufacturing process of this vaccine. Once prequalified by WHO, this vaccine could be an option for purchase by UN agencies.

**Funding:**

The Bill & Melinda Gates Foundation.

**Translation:**

For the Nepali translation of the abstract see Supplementary Materials section.

## Introduction

Typhoid fever, caused by *Salmonella enterica* serovar Typhi (*S* Typhi), is an important cause of morbidity and mortality in low-income and middle-income countries.^1^, ^2^ Typhoid fever is more common in children and young adults than in older people, and is most prevalent in impoverished and overcrowded areas with poor sanitation.^2^, ^3^, ^4^ Although typhoid fever can be effectively treated with antibiotics, the growing rates of antibiotic resistance in many countries are making available treatment options increasingly less effective and costly.^5^ Early intervention with vaccination, especially in children younger than 2 years, is essential.^6^, ^7^ The 2018 WHO position paper recommends that countries should consider the use of typhoid vaccines for high-risk groups and populations, and for outbreak control.^8^ In endemic countries, control of typhoid fever would require implementing immunisation of young children (ie, those aged up to 15 years) and incorporating the typhoid vaccine in the Expanded Program on Immunization schedule.^7^, ^9^

The Vi polysaccharide vaccine, produced by Sanofi Pasteur (Lyon, France), is a WHO-prequalified vaccine that is recommended for individuals aged 2 years and older. However, due to the short duration of protective immunity, re-vaccination every 3 years is advised.^8^ The limitations of Vi polysaccharide vaccines can be overcome by conjugation of the Vi to a carrier protein. The prototype typhoid conjugate vaccine, Vi-rEPA (developed by the National Institutes of Health, Bethesda, MD, USA), showed an overall efficacy of 89% against typhoid fever (diagnosed by the isolation of *S* Typhi from blood cultures after 3 or more days of fever) after 27 months of active surveillance followed by 19 months of passive surveillance.^10^ The protective efficacy of the Vi polysaccharide-tetanus toxoid (Vi-TT) conjugate vaccine (Typbar TCV; Bharat Biotech India, Hyderabad, India) reported in the Typhoid Vaccine Acceleration Consortium study in Nepal was 81·6% at approximately 9 months after the single-dose vaccination.^11^

**Research in context**

**Evidence before this study**

We searched PubMed and Google Scholar using the search terms “typhoid conjugate vaccine (TCV)”, “Vi-DT conjugate”, “typhoid”, “typhoid vaccine pipeline”, and “vaccine”. We searched for primary research articles and clinical trials published from database inception to Jan 31, 2021, with no language restrictions. We identified two phase 1 studies evaluating the safety and immunogenicity of the Vi polysaccharide-diphtheria toxoid (Vi-DT) vaccine in children and adults. The first phase 1 trial, published in 2018, evaluated the Vi-DT vaccine in 144 healthy Filipino participants, of whom 24 adults (aged 18–45 years), 24 adolescents (aged 6–17 years), and 24 children (aged 2–5 years) were included in the Vi-DT vaccine group. The second study, published in 2019, included 100 Indonesian participants, with 25 adults (aged 18–40 years) and 25 children (aged 2–5 years) included in the Vi-DT vaccine group. There were also two phase 2 studies of the Vi-DT vaccine in children; the first study (published in 2020) in Indonesia included 200 children (aged 2–11 years), with 100 children exposed to the Vi-DT test vaccine, and the second study (published in 2019) in the Philippines included 285 children (aged 6 months to 2 years), with 228 exposed to the Vi-DT vaccine. All of these studies concluded that the Vi-DT vaccine was safe and immunogenic.

**Added value of this study**

This phase 3 trial is a head-to-head comparison between the Vi-DT vaccine, manufactured by SK Bioscience (Seoul, South Korea), and the WHO prequalified Vi polysaccharide-tetanus toxoid (Vi-TT) Typbar TCV vaccine, manufactured by Bharat Biotech (Hyderabad, India), assessing the safety and immunogenicity of these vaccines in healthy individuals aged 6 months to 45 years in Nepal; a country where typhoid fever is highly endemic. Our results show that the Vi-DT vaccine is non-inferior to the Typbar TCV vaccine and has a similar safety profile.

**Implications of all the available evidence**

Our findings show that the Vi-DT vaccine is safe and immunogenic in all age groups, including in children aged 6–23 months. WHO recommends that introduction of the typhoid conjugate vaccine should be prioritised in countries with a high burden of typhoid disease. There are, however, only two WHO-prequalified typhoid conjugate vaccines with limited supply. The findings of our trial will be important for local licensure in the country of manufacture and WHO prequalification of the Vi-DT vaccine, contributing to filling the gap in supply and meeting an important public health need of resource-constrained countries.

Four typhoid conjugate vaccines from different sources, and with different carrier proteins for conjugation, are commercially available in India: Typbar TCV, PedaTyph (Bio-Med, Ghaziabad, India), the ZYVAC typhoid conjugate vaccine (Zydus Cadila, Ahmedabad, India; India Clinical Trials Registry India CTRI/2016/05/006975), and the recently (March, 2020) licensed TYPHIBEV vaccine containing Vi polysaccharide conjugated to the CRM197 protein (Biological E, Hyderabad, India).^12^, ^13^, ^14^, ^15^ Based on immunogenicity data, WHO prequalification was awarded to Bharat Biotech for Typbar TCV in January 2018, and to Biological E for TYPHIBEV in December 2020.

The International Vaccine Institute and SK Bioscience (both in Seoul, South Korea) are developing a typhoid conjugate vaccine comprised of purified Vi polysaccharide derived from *S* Typhi strain C6524 conjugated to diphtheria toxoid (Vi-DT), with the overall aim of attaining WHO prequalification and subsequently contributing to the global supply of typhoid conjugate vaccines. The phase 1 and 2 studies of the Vi-DT vaccine were done in the Philippines and showed the safety and immunogenicity of this vaccine when given at a dose of 25 μg to participants aged 6 months to 45 years in a two-dose regimen.^16^, ^17^ Herein, we report the phase 3 trial results of the immunogenicity and safety of a single dose of Vi-DT versus a single dose of Vi-TT vaccine at 4 weeks after vaccination.

## Methods

### Study design

We did a an observer-blind, active-controlled, randomised, non-inferiority, phase 3 trial comparing the immunogenicity and safety of a single dose of Vi-DT vaccine (SK Bioscience) versus a single dose of Vi-TT vaccine (Typbar TCV; Bharat Biotech International, Hyderabad, India). This study was done at four hospitals in Nepal: Kanti Children's Hospital (Kathmandu); BP Koirala Institute of Health Sciences (Dharan); Dhulikhel Hospital, Kathmandu University Hospital (Dhulikhel); and Nepalgunj Medical College (Nepalgunj). The trial and participant enrolment were approved by the Nepal Health Research Council (Nepal), the Department of Drug Administration (Nepal), the Institutional Review Board of the International Vaccine Institute, and the four participating hospital sites.

### Participants

Eligible participants were healthy individuals aged 6 months to 45 years, who themselves or their parents or legal guardians were willing to provide informed consent and to follow the study procedures, and who were available for the duration of the study (appendix 2 pp 3–4). Patients with an acute or chronic illness that could interfere with interpretation of the study endpoints, or who were involved in any other clinical trial were excluded. All participants, parents, or legal guardians provided written (signed and dated) informed consent or assent before participation in the screening activities. A copy of the informed consent or assent document was given to the participants, parents, or legal guardians for their records.

### Randomisation and masking

Eligible participants were randomly assigned (1:1:1:1) to one of four study groups (groups A–D), stratified by age (6 months to <2 years, 2 years to <18 years, and 18 years to 45 years), in which participants in groups A–C received the Vi-DT vaccine from one of three good manufacturing practice lots (group A received lot 1, group B received lot 2, and group C received lot 3) and participants in group D received the licensed Vi-TT vaccine comparator. An independent statistician generated the randomisation list for each of the four study sites. The randomisation list contained sequential numbers unique to each participant and vaccination group (A–D), and a block randomisation process, with random block sizes of four and eight, were used to ensure an effective balance between the interventions. Two types of randomisation lists, one with randomisation numbers only, and a second with randomisation numbers and vaccine allocation, were prepared. The randomisation list without information on vaccine allocation was given to the masked site staff for enrolling the trial participants and assigning them a randomisation number. The randomisation list with information on vaccine allocation was given to the unmasked vaccine administrator (ie, a nurse or pharmacist).

After eligibility assessment and baseline blood draws had been completed, participants were directed to the unmasked vaccine administrator located in a separate room. The randomisation number of participants was written on the empty vaccine vial and recorded in the vaccine accountability log for reconciliation.

Other than the unmasked study staff, site staff remained masked to vaccine allocation. The unmasked study nurse was not involved in the evaluation of vaccine safety and was not allowed to discuss the administered vaccines with the investigator and clinical staff. All participants and outcome assessors were masked to group assignment.

### Procedures

The schedule of events is summarised in appendix 2 (pp 5–6). After informed consent was obtained and the predefined inclusion and exclusion criteria were met, participants were given 0·5 mL (25 μg) of either Vi-DT vaccine or Vi-TT vaccine via intramuscular injection, preferably in the left anterolateral thigh or the left deltoid region. Participants were observed at the study site for at least 30 min after vaccination for any immediate reactions. Eligible participants aged 9–15 months received the primary dose of a locally licensed measles-rubella vaccine as a concomitant vaccine.

Solicited adverse events were recorded during 7 days after vaccination and unsolicited adverse events were recorded until 4 weeks post vaccination in a diary card. Serious adverse events were recorded throughout the 24-week study period in a diary card, and participants or their legal guardians were contacted by telephone. Each participant's weight, height, heart rate, respiratory rate, and body temperature were checked at each study visit as part of the safety evaluation.

For immunogenicity analyses, blood samples were taken at baseline (day 0) before vaccination, and at day 28 (week 4) and day 168 (week 24) after vaccination. Anti-Vi IgG antibody concentrations were measured with an in-house ELISA, as previously described,^17^, ^18^ and antibody titres (measured in international units [IUs] per mL) were measured by use of a WHO international standard reference panel from the National Institute for Biological Standards and Control (NIBSC 16/138; appendix 2 p 8). The lower limit of detection for anti-Vi IgG titres was 0·14 IU/mL.

### Outcomes

The co-primary endpoints were: (1) non-inferiority of immunogenicity of the Vi-DT vaccine (pooled groups A–C) versus the Vi-TT vaccine (group D) at 4 weeks after vaccination, measured by the anti-Vi IgG seroconversion rate, which was defined as the proportion of participants with a four-fold or greater increase in antibody titres compared with baseline; and (2) the lot-to-lot consistency of the Vi-DT vaccine, measured by immune equivalence of the anti-Vi IgG geometric mean titre (GMT) at 4 weeks after receipt of the three Vi-DT vaccine lots (lot 1 *vs* lot 2, lot 1 *vs* lot 3, and lot 2 *vs* lot 3).

Secondary endpoints were the seroconversion rates at 24 weeks after vaccination; anti-Vi IgG GMTs and GMT ratios at baseline compared with 24 weeks after vaccination; the seroconversion rates of anti-Vi IgG antibody titres at 4 weeks across the three Vi-DT vaccine lots; measles and rubella IgG antibodies following a single dose of the measles-rubella vaccine at baseline and at 4 weeks post-vaccination; and non-inferiority of immunogenicity of the Vi-DT vaccine (groups A–C) to the Vi-TT vaccine (group D) at 4 weeks after vaccination, measured by the anti-Vi IgG GMT (appendix 2 p 7). Results on immunological non-interference of the Vi-DT vaccine with the measles-rubella vaccine (ie, ELISA IgG antibody titres against measles and rubella) are not presented herein and will be published elsewhere.

The frequency of local and systemic solicited adverse events during 7 days after vaccination, the frequency of unsolicited adverse events during 28 days after vaccination, and the occurrence of serious adverse events throughout the study were safety endpoints.

### Statistical analysis

The sample size of 450 participants per vaccine group provided 99% power to determine non-inferiority of immunogenicity in Vi-DT vaccine groups A–C combined (n=1350) compared with Vi-TT vaccine group D (n=450), according to the seroconversion rate. The assumed seroconversion rate of the Vi-TT vaccine was 90% (conservatively assumed from the seroconversion rate of 95–98% observed in a previous phase 3 trial of the Vi-TT vaccine in India^13^). Non-inferiority of the Vi-DT vaccine compared with the Vi-TT vaccine was shown if the lower limit of the 97·5% CI for the difference between the seroconversion rates in Vi-DT vaccine groups A–C combined versus Vi-TT vaccine group D was above the predefined non-inferiority margin of −10%, as recommended by the WHO Technical Report Series 924.^19^ A one-sided test of non-inferiority was used with a significance level of 0·0125. This sample size also provided 97% power for three equivalence tests of anti-Vi IgG GMT ratios among the three Vi-DT vaccine lot-to-lot comparisons with an overall two-sided significance level of 0·025. Each two-sided equivalence test had 97% power to show the equivalence of anti-Vi IgG GMTs in each lot-to-lot comparison, each with a two-sided significance level of 0·0083. Lot-to-lot immune equivalence was shown if the upper and lower bounds of the two-sided 99·17% CI (for all age strata) or 95% CI (for individual age strata) around the GMT ratio for each pairwise lot-to-lot comparison was between 0·67 and 1·50, which is the predefined equivalence margin recommended by the WHO Technical Report Series 924.^19^ The true anti-Vi IgG GMT ratio was assumed as 1·0, and the coefficient of variation of the titre of immunogenicity was assumed as 2·0. The coefficient of variation of anti-Vi IgG GMTs in the Vi-DT vaccine group was assumed conservatively on the basis of phase 1 trial data.^16^ In both sample size calculations, a 15% drop-out rate was assumed, considering potential losses to follow-up and withdrawal of consent, as per experience from participating sites.

For the analysis of the secondary endpoint of immune non-inferiority in terms of anti-Vi IgG GMTs, non-inferiority of the Vi-DT vaccine was shown when the lower limit of the two-sided 95% CI around the GMT ratio for Vi-DT vaccine groups A–C combined versus Vi-TT vaccine group D at 4 weeks after vaccination was greater than the non-inferiority margin of 0·67. For the analysis of the secondary endpoint of the lot-to-lot consistency in terms of seroconversion rates, the equivalence of two lots was shown if the upper and lower bounds of the two-sided 98·33% CI (for all age strata) or 95% CI (for individual age strata) around the difference in seroconversion rate for each pairwise lot-to-lot comparison was within the equivalence margin of −10% to 10%. At 24 weeks, secondary immunogenicity endpoints were summarised descriptively by group and age strata.

The intention-to-treat analysis included all randomised participants. The full analysis set included all randomised participants who had received their assigned study vaccine (modified intention-to-treat). The immunogenicity analysis set included all participants who had received their assigned study vaccine and had completed at least one post-baseline immunogenicity assessment. The per-protocol analysis set included all randomised participants who did not have any major protocol deviations, defined as those that jeopardised the safety or rights of the participant or the scientific integrity of the study (eg, violation of inclusion and exclusion criteria, attendance of a follow-up visit outside the prespecified visit window, or the measles-rubella vaccine was given to participants outside the prespecified age range). A protocol deviation was considered minor if blood sample collection at the 4-week visit was done within 14 days of the planned allowable visit window; if the blood sample was collected more than 14 days after the planned allowable visit window, it was considered a major protocol deviation. The co-primary immunogenicity endpoints were assessed in the immunogenicity analysis set. Demographics and safety endpoints were assessed in the full analysis set. Safety endpoints were descriptively summarised by group and age strata. All adverse events were classified by system organ class and preferred terms of the Medical Dictionary for Regulatory Activities (version 22.0). A per-protocol analysis was done as a sensitivity analysis for the primary and secondary immunogenicity endpoints.

Statistical analyses were done using SAS 9.4. All analyses were done according to the study protocol and the statistical analysis plan (appendix 3). p values for categorical variables were calculated by use of χ^2^ tests. An independent data safety management board, consisting of independent clinical experts and a biostatistician, monitored the safety aspects of this study, according to data safety monitoring board charter guidelines.

This trial is registered with ClinicalTrials.gov, NCT03933098.

### Role of the funding source

The funder of the study had no role in study design, data collection, data analysis, data interpretation, or writing of the report.

## Results

Between Nov 20, 2019, and March 10, 2020, 1854 individuals aged 6 months to 45 years were screened, and 1800 were enrolled and randomly assigned to groups A, B, C, or D (450 participants to each group); all screening failures were due to pre-existing medical conditions (figure). The full analysis set included all 1800 participants, and the immunogenicity analysis set included 1786 participants (443 in group A, 450 in group B, 447 in group C, and 446 in group D). A total of 33 participants were excluded from the per-protocol analysis set; five were lost to follow-up, four withdrew consent, and 24 had major protocol deviations, mainly due to missing the follow-up visit at 4 weeks or attending this follow-up visit outside the predefined visit window (appendix 2 p 31). Most visits that occurred outside the predefined visit window were due to travel restrictions introduced by local government because of the COVID-19 pandemic.

**Figure:**
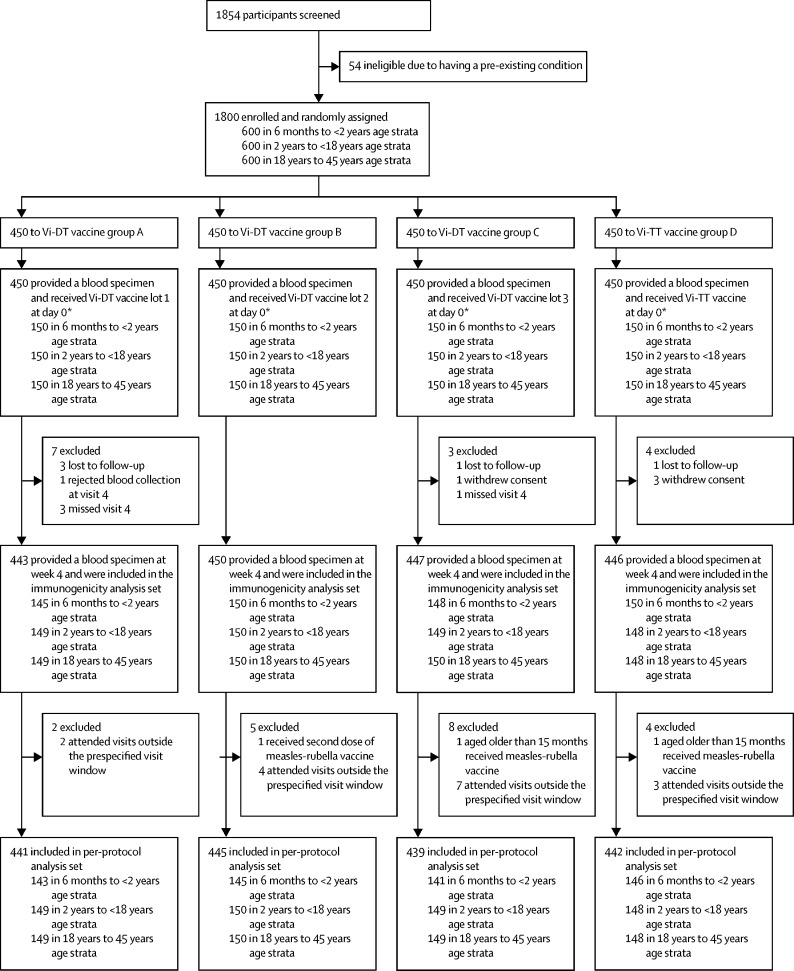
Trial profile Vi-DT=Vi polysaccharide-diphtheria toxoid. Vi-TT=Vi polysaccharide-tetanus toxoid. *These participants were included in the full analysis set, which was used to assess safety.

The age and sex distributions of participants across groups A–D and by age stratum were well balanced (table 1). Data on the participant's weight, height, heart rate, respiratory rate, and body temperature showed that the mean and median values for all vital signs were similar between Vi-DT vaccine groups A–C and Vi-TT vaccine group D at each visit, and between each lot of Vi-DT vaccine received (data not shown).

**Table 1:** Age and sex distribution of randomised participants by age stratum

		**Vi-DT vaccine group**				**Vi-TT vaccine group (n=450)**	**Total (n=1800)**
		All lots (n=1350)	Lot 1 (n=450)	Lot 2 (n=450)	Lot 3 (n=450)		
**Overall**							
Sex							
	Male	681 (50·4%)	226 (50·2%)	223 (49·6%)	232 (51·6%)	222 (49·3%)	903 (50·2%)
	Female	669 (49·6%)	224 (49·8%)	227 (50·4%)	218 (48·4%)	228 (50·7%)	897 (49·8%)
Age, years		13·2 (12·1)	13·0 (11·7)	13·5 (12·4)	13·3 (12·3)	13·0 (12·1)	13·2 (12·1)
**Age 6 months to <2 years**							
Number of participants		450 (33·3%)	150 (33·3%)	150 (33·3%)	150 (33·3%)	150 (33·3%)	600 (33·3%)
Sex							
	Male	232 (51·6%)	79 (52·7%)	77 (51·3%)	76 (50·7%)	81 (54·0%)	313 (52·2%)
	Female	218 (48·4%)	71 (47·3%)	73 (48·7%)	74 (49·3%)	69 (46·0%)	287 (47·8%)
Age, years		14·1 (4·7)	14·7 (4·9)	13·9 (4·6)	13·7 (4·7)	14·1 (4·7)	12·5 (4·3)
**Age 2 years to <18 years**							
Number of participants		450 (33·3%)	150 (33·3%)	150 (33·3%)	150 (33·3%)	150 (33·3%)	600 (33·3%)
Sex							
	Male	247 (54·9%)	78 (52·0%)	80 (53·3%)	89 (59·3%)	69 (46·0%)	316 (52·7%)
	Female	203 (45·1%)	72 (48·0%)	70 (46·7%)	61 (40·7%)	81 (54·0%)	284 (47·3%)
Age, years		10·6 (4·5)	10·7 (4·5)	10·8 (4·6)	10·4 (4·4)	10·2 (4·5)	10·5 (4·5)
**Age 18 years to 45 years**							
Number of participants		450 (33·3%)	150 (33·3%)	150 (33·3%)	150 (33·3%)	150 (33·3%)	600 (33·3%)
Sex							
	Male	202 (44·9%)	69 (46·0%)	66 (44·0%)	67 (44·7%)	72 (48·0%)	274 (45·7%)
	Female	248 (55·1%)	81 (54·0%)	84 (56·0%)	83 (55·3%)	78 (52·0%)	326 (54·3%)
Age, years		27·9 (7·3)	27·1 (6·9)	28·5 (7·6)	28·2 (7·4)	27·8 (7·3)	27·9 (7·3)

Data are n (%) or mean (SD). Vi-DT=Vi polysaccharide-diphtheria toxoid. Vi-TT=Vi polysaccharide-tetanus toxoid.

The anti-Vi-IgG seroconversion rates at 4 weeks after vaccination in all age strata were 99·33% (97·5% CI 98·61 to 99·68; 1331 of 1340 participants) in Vi-DT vaccine groups A–C and 98·88% (97·10 to 99·57; 441 of 446 participants) in Vi-TT vaccine group D. The difference between proportions was 0·47% (97·5% CI −0·68 to 1·61) in all age strata. The Vi-DT vaccine was considered non-inferior to the Vi-TT vaccine, as the lower limit of the 97·5% CI for the difference in seroconversion rates between the two groups was greater than the predefined non-inferiority limit of −10%. The anti-Vi-IgG seroconversion rate at 4 weeks in participants aged 6 months to younger than 2 years was 98·87% (95% CI 97·39 to 99·52; 438 of 443 participants) in Vi-DT vaccine groups A–C versus 98·67% (95·27 to 99·63; 148 of 150) in Vi-TT vaccine group D; in those aged 2 years to younger than 18 years was 99·78% (98·75 to 99·96 447 of 448) in Vi-DT vaccine groups A–C versus 99·32% (96·27 to 99·88; 147 of 148) in Vi-TT vaccine group D; and in those aged 18 years to 45 years was 99·33% (98·05 to 99·77; 446 of 449) in Vi-DT vaccine groups A–C versus 98·65% (95·21 to 99·63; 146 of 148) in Vi-TT vaccine group D. The difference in seroconversion rates between Vi-DT vaccine groups A–C and Vi-TT vaccine group D was 0·20% (95% CI −2·78 to 3·19) in participants aged 6 months to younger than 2 years, 0·45% (−2·42 to 3·32) in those aged 2 years to younger than 18 years, and 0·68% (−2·28 to 3·64) in those aged 18 years to 45 years. Non-inferiority of the Vi-DT vaccine across the three age strata was shown, as the lower limit of the two-sided 95% CI of the difference in seroconversion rates between Vi-DT vaccine groups A–C and Vi-TT vaccine group D was greater than the non-inferiority margin of −10%. In a sensitivity analysis involving participants in the per-protocol analysis set, the difference between the anti-Vi-IgG seroconversion rates between Vi-DT vaccine groups A–C and Vi-TT vaccine group D remained non-inferior in all age strata (0·47% [97·5% CI −0·69 to 1·62]) and in each individual age strata (appendix 2 p 9).

In the analysis of lot-to-lot consistency of the Vi-DT vaccine, each lot yielded anti-Vi IgG GMTs at 4 weeks post-vaccination of greater than 440 IU/mL in all age strata; the anti-Vi IgG GMT ratio of lot 1 versus lot 2 was 1·02 (99·17% CI 0·85–1·22), of lot 1 versus lot 3 was 1·02 (0·85–1·23), and of lot 2 versus lot 3 was 1·01 (0·84–1·21). Therefore, lot-to-lot equivalence was shown, as the upper and lower bounds of the two-sided 99·17% CI around the GMT ratio for each pairwise lot-to-lot comparison was between 0·67 and 1·50 (table 2). Lot-to-lot equivalence in each age stratum was also shown. Anti-Vi IgG GMT ratios in the per-protocol analysis set verified lot-to-lot equivalence in all participants and in each age stratum, as the 99·17% CIs (for all participants) or 95% CIs (for each age stratum) around the GMT ratios were also within the equivalence margin (appendix 2 p 10).

**Table 2:** Lot-to-lot consistency of anti-Vi IgG GMTs in participants who received the Vi-DT vaccine at week 4 in the immunogenicity analysis set

		**Number of participants**	**GMT, IU/mL**	Lot 1 *vs* lot 2 GMT ratio*	Lot 1 *vs* lot 3 GMT ratio*	Lot 2 *vs* lot 3 GMT ratio*
All ages		..	..	1·02 (99·17% CI 0·85–1·22)	1·02 (99·17% CI 0·85–1·23)	1·01 (99·17% CI 0·84–1·21)
	Lot 1	443	450·54 (99·17% CI 395·58–513·13)	..	..	..
	Lot 2	450	443·70 (99·17% CI 390·42–504·25)	..	..	..
	Lot 3	447	440·83 (99·17% CI 387·35–501·69)	..	..	..
Age 6 months to <2 years		..	..	1·02 (95% CI 0·86–1·22)	1·20 (95% CI 0·98–1·47)	1·17 (95% CI 0·97–1·41)
	Lot 1	145	541·03 (95% CI 472·15–619·96)	..	..	..
	Lot 2	150	528·81 (95% CI 474·11–589·82)	..	..	..
	Lot 3	148	451·53 (95% CI 387·99–525·47)	..	..	..
Age 2 years to <18 years		..	..	1·02 (95% CI 0·83–1·25)	0·92 (95% CI 0·74–1·15)	0·90 (95% CI 0·73–1·11)
	Lot 1	149	410·48 (95% CI 352·60–477·87)	..	..	..
	Lot 2	150	402·84 (95% CI 350·68–462·77)	..	..	..
	Lot 3	149	445·14 (95% CI 380·55–520·70)	..	..	..
Age 18 years to 45 years		..	..	1·01 (95% CI 0·75–1·36)	0·97 (95% CI 0·74–1·28)	0·96 (95% CI 0·72–1·29)
	Lot 1	149	413·82 (95% CI 337·54–507·34)	..	..	..
	Lot 2	150	410·05 (95% CI 328·03–512·57)	..	..	..
	Lot 3	150	426·37 (95% CI 352·51–515·71)	..	..	..

GMT=geometric mean titre. Vi-DT=Vi polysaccharide-diphtheria toxoid.

* The equivalence of two vaccine lots was shown if the upper and lower bounds of the two-sided 99·17% CI (for all ages) or 95% CI (for individual age strata) around the GMT ratio for each pairwise lot-to-lot comparison was between 0·67 and 1·50.

In the immunogenicity analysis set, the anti-Vi IgG GMT ratio of Vi-DT vaccine groups A–C compared with Vi-TT vaccine group D was 1·17 (95% CI 1·05–1·30) in all age strata, 1·20 (0·99–1·45) in participants aged 6 months to younger than 2 years, 0·93 (0·80–1·08) in those aged 2 years to younger than 18 years, and 1·43 (1·18–1·74) in those aged 18 years to 45 years. Non-inferiority of the Vi-DT vaccine was shown when a lower limit of the two-sided 95% CI around the GMT ratio for Vi-DT vaccine groups A–C to Vi-TT vaccine group D was greater than the non-inferiority margin of 0·67 (appendix 2 pp 11–12). Using the same margin, non-inferiority of the Vi-DT vaccine was also shown in all participants and in each age stratum in the per-protocol analysis set (appendix 2 p 13).

The lot-to-lot consistency of the Vi-DT vaccine in terms of the anti-Vi IgG seroconversion rates at week 4 in the immunogenicity analysis set is shown in table 3. In all age strata, the lot-to-lot difference in anti-Vi IgG seroconversion rates were −0·69 (98·33% CI −2·10 to 0·73) for lot 1 versus lot 2, −0·68 (−2·10 to 0·73) for lot 1 versus lot 3, and 0·01 (−1·06 to 1·08) for lot 2 versus lot 3. The results show lot-to-lot equivalence, as the upper and lower bounds of the two-sided 98·33% CI around the difference in seroconversion rate for each pairwise lot-to-lot comparison was within the equivalence margin of −10 to 10% (table 3; appendix 2 p 14). Lot-to-lot equivalence was shown for each individual age stratum (based on the 95% CIs; table 3), and in the per-protocol analysis set (appendix 2 pp 15–16).

**Table 3:** Vi-DT vaccine lot-to-lot consistency in anti-Vi IgG seroconversion rates at week 4 in the immunogenicity analysis set

		**n/N**	**Seroconversion rate**	Lot 1 *vs* lot 2 difference*	Lot 1 *vs* lot 3 difference*	Lot 2 *vs* lot 3 difference*
All ages		..	..	−0·69 (98·33% CI −2·10 to 0·73)	−0·68 (98·33% CI −2·10 to 0·73)	0·01 (98·33% CI −1·06 to 1·08)
	Lot 1	438/443	98·87% (98·33% CI 96·90–99·59)	..	..	..
	Lot 2	448/450	99·56% (98·33% CI 97·96–99·90)	..	..	..
	Lot 3	445/447	99·55% (98·33% CI 97·95–99·90)	..	..	..
Age 6 months to <2 years		..	..	0·64 (95% CI −4·55 to 5·83)	0·66 (95% CI −4·53 to 5·86)	0·02 (95% CI −5·13 to 5·17)
	Lot 1	144/145	99·31% (95% CI 96·20–99·88)	..	..	..
	Lot 2	148/150	98·67% (95% CI 95·27–99·63)	..	..	..
	Lot 3	146/148	98·65% (95% CI 95·21–99·63)	..	..	..
Age 2 years to <18 years		..	..	−0·67 (95% CI −5·62 to 4·28)	−0·67 (95% CI −5·64 to 4·30)	0·00 (95% CI 0·00 to 0·00)
	Lot 1	148/149	99·33% (95% CI 96·30–99·88)	..	..	..
	Lot 2	150/150	100·00% (95% CI 97·50–100·00)	..	..	..
	Lot 3	150/150	100·00% (95% CI 97·49–100·00)	..	..	..
Age 18 years to 45 years		..	..	−2·01 (95% CI −7·25 to 3·22)	−2·01 (95% CI −7·25 to 3·22)	0·00 (95% CI 0·00 to 0·00)
	Lot 1	146/149	97·99% (95% CI 94·25–99·31)	..	..	..
	Lot 2	150/150	100·00 (95% CI 97·50–100·00)	..	..	..
	Lot 3	150/150	100·00 (95% CI 97·50–100·00)	..	..	..

n=number of participants seroconverted. N=total number of participants. The seroconversion rate is defined as the proportion of participants who had a four-fold or greater increase in anti-Vi antibody titres compared with baseline.

* The equivalence of two lots was shown if the upper and lower bounds of the two-sided 98·33% CIs (for all ages) or 95% CIs (individual age strata) around the difference in seroconversion rate for each pairwise lot-to-lot comparison was within the equivalence margin of −10% to 10%.

In 107 participants aged 9–15 months in the immunogenicity analysis set who received a concomitant measles-rubella vaccine, the anti-Vi IgG seroconversion rates were 100%, both in Vi-DT plus measles-rubella vaccine groups A–C (95% CI 95·25–100·00) and Vi-TT plus measles-rubella vaccine group D (88·65–100·0) at 4 weeks following vaccination, indicating non-interference of the measles-rubella vaccine with the Vi-DT and Vi-TT vaccines. Anti-Vi IgG GMTs were 501·50 IU/mL (95% CI 423·11–594·41) in Vi-DT vaccine plus measles-rubella vaccine groups A–C and 464·38 IU/mL (366·80–587·92) in Vi-TT vaccine plus measles-rubella vaccine group D (appendix 2 p 17). These high seroconversion rates were verified in the per-protocol analysis set, in which anti-Vi IgG seroconversion rates were 100%, both in Vi-DT vaccine plus measles-rubella vaccine groups A–C (95% CI 94·87–100·00) and Vi-TT vaccine plus measles-rubella vaccine group D (87·94 −100·00), whereas the anti-Vi GMTs were 529·09 IU/mL (95% CI 444·85–629·29) in Vi-DT vaccine plus measles-rubella vaccine groups A–C and 494·09 IU/mL (95% CI 390·44–625·25) in Vi-TT vaccine plus measles-rubella vaccine group D.

At 24 weeks (168 days) post-vaccination, the anti-Vi IgG seroconversion rate in Vi-DT vaccine groups A–C in all age strata was 98·54% (95% CI 97·70–99·07; 1213 of 1231 participants) compared with 97·26% (95·17–98·47; 391 of 402) in Vi-TT vaccine group D (appendix 2 p 18). In Vi-DT vaccine groups A–C, seroconversion rates were 98·66% (96·89–99·42; 367 of 372) in participants aged 6 months to younger than 2 years, 99·32% (98·01–99·77; 435 of 438) in those aged 2 years to younger than 18 years, and 97·62% (95·68–98·70; 411 of 421) in those aged 18 years to 45 years. In Vi-TT vaccine group D, seroconversion rates were 100·00% (97·04–100·00; 126 of 126) in participants aged 6 months to younger than 2 years, 97·22% (93·08–98·91; 140 of 144) in those aged 2 years to younger than 18 years, and 94·70% (89·46–97·41; 125 of 132) in those aged 18 years to 45 years. Similar seroconversion rates were observed in the per-protocol analysis set (appendix 2 p 19).

In all age strata, the anti-Vi IgG GMT at 24 weeks after vaccination was 88·07 IU/mL (95% CI 83·04–93·41) in Vi-DT vaccine groups A–C and 99·31 IU/mL (91·07–108·29) in Vi-TT vaccine group D (Vi-DT groups A–C *vs* Vi-TT group D p=0·017; appendix 2 p 20). In participants aged 6 months to younger than 2 years, the anti-Vi IgG GMT in Vi-DT vaccine groups A–C at 24 weeks after vaccination was 50·17 IU/mL (47·07–53·48), which was significantly lower than in Vi-TT vaccine group D (73·41 IU/mL [65·52–82·26]; p<0·0001). In participants aged 2 years to younger than 18 years, the anti-Vi IgG GMT was 92·87 IU/mL (85·01–101·44) in Vi-DT vaccine groups A–C and 110·03 IU/mL (94·30–128·39) in Vi-TT vaccine group D (p=0·061), and in participants aged 18 years to 45 years, the anti-Vi IgG GMT was 137·03 IU/mL (95% CI 121·87–154·07) in Vi-DT vaccine groups A–C and 118·47 IU/mL (100·61–139·51) in the Vi-TT vaccine group D (p=0·15). A similar trend was observed in the per-protocol analysis set (appendix 2 p 21).

A total of 20 participants reported immediate reactions during the first 30 min post vaccination; 16 (1·2%) of 1350 participants in Vi-DT vaccine groups A–C (three participants who received lot 1, five who received lot 2, and eight who received lot 3) and four (0·9%) of 450 participants in Vi-TT vaccine group D (appendix 2 pp 24–25). All reported immediate reactions were mild to moderate in severity. The most common immediate reaction in all groups was pain and tenderness at the injection site (14 participants), observed in 11 participants in Vi-DT vaccine groups A–C (two participants who received lot 1, three who received lot 2, and six who received lot 3) and three in Vi-TT vaccine group D. Four participants had erythema or redness, including three in Vi-DT vaccine groups A–C (one participant per each lot of Vi-DT vaccine who received the concomitant measles-rubella vaccine) and one participant in Vi-TT vaccine group D. One participant in the 2 years to younger than 18 years age stratum reported headache after receiving lot 2 of the Vi-DT vaccine, and one participant in the same age stratum who received lot 3 of the Vi-DT vaccine had vomiting. Immediate reactions did not differ in frequency between the individual Vi-DT vaccine groups A–C and Vi-TT vaccine group D.

A total of 644 solicited adverse events (453 in Vi-DT vaccine groups A–C and 191 in Vi-TT vaccine group D) were reported by 375 participants within 7 days following vaccination; in 260 (19·3%) of 1350 participants in Vi-DT vaccine groups A–C and in 115 (25·6%) of 450 in Vi-TT vaccine group D (table 4). The most frequent systemic solicited adverse events reported were fever, headache, vomiting, and diarrhoea (appendix 2 pp 26–27). Most adverse events were mild to moderate in severity and resolved within a few days. There were ten solicited adverse events classified as severe, seven in Vi-DT vaccine groups A–C (two [both with fever] who received lot 1, one [with erythema] who received lot 2, and four [one with pain, one with diarrhoea, one with vomiting, and one with lethargy] who received lot 3) and three (two with erythema and one with fever) in Vi-TT vaccine group D. Of these severe solicited adverse events, nine were reported in the 6 months to younger than 2 years age stratum and one was reported in the 2 years to younger than 18 years age stratum.

**Table 4:** Proportion of participants with solicited adverse events within 7 days after vaccination

	**Vi-DT vaccine group**				**Vi-TT vaccine group**	Vi-DT vaccine lot 1 plus lot 2 plus lot 3 *vs* Vi-TT vaccine p value*	Vi-DT vaccine lot 1 *vs* lot 2 *vs* lot 3 *vs* Vi-TT vaccine p value*
	All lots	Lot 1	Lot 2	Lot 3			
All ages	260/1350 (19·3%; 17·2–21·5)	81/450 (18·0%; 14·7–21·8)	91/450 (20·2%; 16·8–24·2)	88/450 (19·6%; 16·2–23·5)	115/450 (25·6%; 21·7–29·8)	0·0044	0·032
Age 6 months to <2 years	97/450 (21·6%; 18·0–25·6)	31/150 (20·7%;15·0–27·8	29/150 (19·3%; 13·8–26·4)	37/150 (24·7%; 18·5–32·1)	42/150 (28·0%; 21·4–35·7)	0·11	0·27
Age 2 years to <18 years	70/450 (15·6%; 12·5–19·2)	23/150 (15·3%; 10·4–22·0)	28/150 (18·7%; 13·2–25·7)	19/150 (12·7%; 8·3–18·9)	34/150 (22·7%; 16·7–30·0)	0·046	0·12
Age 18 years to 45 years	93/450 (20·7%; 17·2–24·7)	27/150 (18·0%; 12·7–24·9)	34/150 (22·7%; 16·7–30·0)	32/150 (21·3%; 15·5–28·6)	39/150 (26·0%; 19·6–33·6)	0·17	0·41

Data are n/N (%; 95% CI), unless otherwise indicated. n=number of participants who reported events. N=total number of participants. Vi-DT=Vi polysaccharide-diphtheria toxoid. Vi-TT=Vi polysaccharide-tetanus toxoid.

* p values were calculated by use of χ^2^ tests.

There were 504 unsolicited adverse events reported within 4 weeks of vaccination, including 361 events in Vi-DT vaccine groups A–C (110 in those who received lot 1, 135 in those who received lot 2, and 116 in those who received lot 3) and 143 events in Vi-TT vaccine group D (table 5). Most unsolicited adverse events were classified as mild to moderate in severity, none were related to the study vaccine, and all resolved with no sequelae. There was no difference in the frequency of unsolicited adverse events observed between Vi-DT vaccine groups A–C (208 [15·4%] of 1350 participants) and Vi-TT vaccine group D (76 [16·9%] of 450). Diarrhoea, vomiting, pyrexia, nasopharyngitis, and cough were the most common unsolicited adverse events reported across all age strata (appendix 2 pp 28–30).

**Table 5:** Proportion of participants with unsolicited adverse events within 4 weeks after vaccination

	**Vi-DT vaccine group**				**Vi-TT vaccine group**	Vi-DT vaccine lot 1 plus lot 2 plus lot 3 *vs* Vi-TT vaccine p value*	Vi-DT vaccine lot 1, lot 2, lot 3, and Vi-TT vaccine p value*
	All lots	Lot 1	Lot 2	Lot 3			
All ages	208/1350 (15·4%; 13·6–17·4)	63/450 (14·0%; 11·1–17·5)	74/450 (16·4%; 3·3–20·2)	71/450 (15·8%; 12·7–19·4)	76/450 (16·9%; 13·7–20·6)	0·46	0·65
Age 6 months to <2 years	137/450 (30·4%; 26·7–34·9)	37/150 (24·7%; 18·5–32·1)	48/150 (32·0%; 25·1–39·8)	52/150 (34·7%; 27·5–42·6)	54/150 (36·0%; 28·8–43·9)	0·21	0·15
Age 2 years to <18 years	45/450 (10·0%; 7·6–13·1)	17/150 (11·3%; 7·2–17·4)	20/150 (13·3%; 8·8–19·7)	8/150 (5·3%; 2·7–10·2)	16/150 (10·7%; 6·7–16·6)	0·82	0·12
Age 18 years to 45 years	26/450 (5·8%; 4·0–8·3)	9/150 (6·0%; 3·2–11·0)	6/150 (4·0%; 1·9–8·5)	11/150 (7·3%; 4·1–12·7)	6/150 (4·0%; 1·9–8·5)	0·40	0·50

Data are n/N (%; 95% CI), unless otherwise indicated. n=number of participants who reported events. N=total number of participants. Vi-DT=Vi polysaccharide-diphtheria toxoid. Vi-TT=Vi polysaccharide-tetanus toxoid.

* p values were calculated by use of χ^2^ tests.

A total of seven participants reported serious adverse events during the 24-week study period, including four (0·30%) of 1350 participants in Vi-DT vaccine groups A–C (two who received lot 1, one who received lot 2, and one who received lot 3) and in three (0·7%) of 450 in Vi-TT vaccine group D. Three serious adverse events occurred in participants aged 6 months to younger than 2 years and four serious adverse events occurred in those aged 18 years to 45 years. Serious adverse events included pneumonia in participants aged 6 months to younger than 2 years, and gastroenteritis, rotavirus gastroenteritis, voluntary surgical or medical procedures, pleural effusion, and cardiorespiratory arrest in those aged 18 years to 45 years (data not shown). The voluntary surgical or medical procedures, which occurred in one participant each from Vi-DT vaccine groups A–C and Vi-TT vaccine group D, were reported by the BP Koirala Institute of Health Sciences within 28 days of vaccination as abortions in two women aged 25 years and 36 years, both of whom had a negative urine pregnancy test before enrolment. Both women opted for medical termination of the pregnancy. The serious adverse event of acute viral gastroenteritis with mild dehydration was reported 4 days following vaccination with the Vi-DT vaccine in one boy aged 14 months at Nepalgunj Medical College Hospital. This participant was admitted to the paediatric ward, and after 24 h of observation and medical management, they were discharged. Acute rotavirus gastroenteritis, with the classic triad of projectile vomiting, diarrhoea, and fever, was reported in a girl aged 18 months at 4 days after receiving the Vi-DT vaccine at Kanti Children's Hospital. This participant was admitted to the paediatric ward, and after 24 h of medical management, they were discharged in a stable condition. Lower lobe pneumonia was reported in a boy aged 15 months at 28 days after receiving the Vi-DT vaccine at Kanti Children's Hospital. This participant was admitted to the paediatric ward, and after 4 days of medical management, they were discharged on oral medication. The serious adverse event of cardiopulmonary arrest was reported in a man aged 34 years at 40 days after receiving the Vi-TT vaccine at Nepalgunj Medical College Hospital. This participant was admitted to the emergency department for emergency management and, within a few minutes of admission, the patient was declared dead. The serious adverse event of right pleural effusion was reported in a man aged 19 years at 63 days following vaccination with the Vi-TT vaccine at the BP Koirala Institute of Health Sciences. This participant was admitted to the general medical ward and, after 9 days of medical management, they were discharged on anti-tubercular drugs. None of these serious adverse events were judged to be related to the study vaccines by site investigators, and this decision was agreed on by the data safety monitoring board.

## Discussion

Our study shows that the Vi-DT vaccine is immunogenic and non-inferior to the locally licensed, WHO-prequalified Vi-TT vaccine when given as a single dose, with or without a concomitant measles-rubella vaccine, at 4 weeks after vaccination in all age groups and within each age stratum. Equivalence among three good manufacturing practice lots of Vi-DT vaccine was also shown.

Five serious adverse events were reported during 4 weeks after vaccination, none of which were considered related to the Vi-DT or Vi-TT vaccines. The immediate adverse reactions did not differ in frequency between Vi-DT and Vi-TT vaccine groups. There were fewer solicited adverse events reported for all age groups in the Vi-DT vaccine groups A–C than in Vi-TT vaccine group D (19·3*% vs* 25·6%). Unsolicited adverse events were reported in 15·4% of participants in Vi-DT vaccine groups A–C and in 16·9% of those in Vi-TT vaccine group D during 4 weeks post-vaccination. Most unsolicited adverse events were classified as mild to moderate in severity and were judged as unrelated to vaccine administration. There was no difference in the frequency of unsolicited adverse events reported between each lot of Vi-DT vaccine and Vi-TT vaccine group D. Two serious adverse events were reported between 4 weeks and 24 weeks in Vi-TT vaccine groups A–C, both of which were considered unrelated to the study vaccine. Overall, the Vi-DT vaccine was well tolerated, and its safety profile is satisfactory and similar to the safety profile of the Vi-TT vaccine.

We found no difference in the anti-Vi IgG seroconversion rate at 24 weeks between Vi-DT vaccine groups A–C and Vi-TT group D. There was a significant difference in the anti-Vi IgG GMT in all participants at 24 weeks, which was due to a significant difference in participants aged 6 months to younger than 2 years; no differences in participants aged 2 years to younger than 18 years, or those aged 18 years to 45 years were observed. These finding needs to be investigated further to fully understand the reason for these age-specific differences.

Several studies have reported the safety and immunogenicity of the Vi-TT vaccine and other typhoid conjugate vaccines in individuals older than 2 years.^11^, ^20^, ^21^, ^22^, ^23^, ^24^, ^25^ However, data on the safety and immunogenicity of the Vi-TT vaccine in younger children are scarce, except from studies done in Indonesia and India. A recent single-blind study in India, involving a small sample of individuals aged 6 months to 45 years and a short-term follow-up period, compared a locally produced typhoid conjugate vaccine (manufactured by Cadila Healthcare, Ahmedabad, India) with the Typbar TCV Vi-TT vaccine, and showed similar immunogenicity and safety of the two vaccines.^24^ The long-term extension of this study is ongoing, and will evaluate the persistence of antibodies at around 3 years after primary vaccination. The Vi-DT vaccine safety and reactogenicity data are consistent with those reported in other phase 3 studies of the Vi-TT vaccine, and they confirm the safety profile of the phase 2 study of the Vi-DT vaccine in children younger than 2 years.^11^, ^13^, ^17^

Overall, our study shows that the Vi-DT test vaccine is safe, immunogenic, and non-inferior to the licensed Vi-TT vaccine when given as a single dose in all age groups, including in children aged 6 months to younger than 2 years. The Vi-DT vaccine was non-inferior to the Vi-TT vaccine in all participants and in each age stratum in terms of the anti-Vi IgG seroconversion rate, and the Vi-DT vaccine lot-to-lot consistency, measured by anti-Vi IgG GMTs, was shown at 4 weeks following vaccination. Non-inferiority of the Vi-DT vaccine compared with the Vi-TT vaccine measured by anti-Vi IgG GMTs, the lot-to-lot consistency measured by anti-Vi IgG seroconversion rates, and non-interference of the measles-rubella vaccine with the Vi-DT vaccine at 4 weeks post-vaccination were also shown.

Antibiotic resistance is a challenge for the effective treatment of typhoid, which is likely to become increasingly problematic with the spread of multidrug-resistant *S* Typhi strains, as evidenced by a large outbreak of typhoid fever in Pakistan in 2016.^26^ The emergence of antimicrobial-resistant *S* Typhi infections contributed to the recommendations by WHO for the introduction of typhoid conjugate vaccines in populations at high risk of infection. However, there are only two WHO-prequalified typhoid conjugate vaccines, both of which are from India and contain the same carrier protein (Vi-TT), and for which demand currently exceeds supply. The results of our trial will be important for licensure and WHO prequalification of the Vi-DT vaccine. This will be the first commercial vaccine to be developed and manufactured outside of India, providing an important addition to the current supply chain and diversity to the typhoid conjugate vaccine portfolio.

One of the limitations of our study was that a sufficient number of participants eligible for the concomitant measles-rubella vaccine could not be enrolled in the study due to a government-initiated measles-rubella vaccine catch-up campaign that occurred during the course of the study. Therefore, the immune non-interference of typhoid conjugate vaccine with the measles-rubella vaccine, in terms of the concentration of antibodies against measles and rubella, could not be established. Therefore, to achieve the objective of showing immune non-interference of the Vi-DT vaccine with a measles-containing vaccine, a separate, add-on study with sample size of 360 infants aged 9–15 months was planned as an amendment to the current study protocol. These results will be published in a separate publication.

Another limitation of our study was caused by the ongoing COVID-19 pandemic. The associated travel restrictions resulted in an increased number of follow-up visits that occurred out of the predefined visit window, and hence, many protocol deviations. However, these protocol deviations had minor effects on the per-protocol analyses. By conducting the study in Nepal, which is a typhoid-endemic country, there was a possibility of enrolling individuals with subclinical infection or asymptomatic carriers. However, we consider that any potential sources of error were equally distributed among the four randomised groups, and that statistical comparisons were therefore unaffected.

In conclusion, the findings of our study show that a single dose of Vi-DT vaccine elicits seroconversion rates similar to that of the WHO-prequalified Vi-TT typhoid conjugate vaccine. The results also indicate lot-to-lot equivalence of the Vi-DT vaccine, supporting the robustness of the manufacturing process. The overall results show that the Vi-DT vaccine is safe and immunogenic in individuals aged 6 months to 45 years, and they support initiation of the licensure process leading to WHO prequalification.

## Data sharing

Deidentified individual participant data will be made available with publication and on request. Proposals should be directed to the corresponding author. After approval of a proposal, data can be shared through a secure online platform.

## Declaration of interests

JHR, HKP, JHS, YL, and HK are employees of SK Bioscience. JHK is a scientific consultant to SK Bioscience for COVID 19 vaccine research. All remaining authors declare no competing interests.

## Supplementary Materials

Nepali translation of the Summary
