# Supplementary material for: Safety and immunogenicity of the Vi-DT typhoid conjugate vaccine in healthy volunteers in Nepal: an observer-blind, active-controlled, randomised, non-inferiority, phase 3 trial
Source: Lancet Infect Dis. 2022 Apr;22(4):529–40. doi: 10.1016/S1473-3099(21)00455-2 (PMC8942857; doi:10.1016/S1473-3099(21)00455-2)
Supplement: Nepali translation of the Summary [file mmc1.pdf]

THE LANCET
Infectious Diseases

Supplementary appendix 1

This translation in Urdu was submitted by the authors and we reproduce it as supplied. It has not been peer reviewed. *The Lancet*'s editorial processes have only been applied to the original in English, which should serve as reference for this manuscript.

यो नेपाली अनुवाद अनुसन्धानकर्ताहरु बाट प्रेषण गरिएको हो र हामीले जस्ताकोतस्तै प्रकाशन गरेका छौं । समकक्षीहरुबाट यसको समीक्षा गरिएको छैन । अंग्रेजी भाषामा रहेको मूल आलेखमा मात्रै 'द ल्यान्सेट' को सम्पादकीय प्रक्रियाहरु पूरा गरिएको छ र त्यसलाई नै यो पाण्डुलिपिको सन्दर्भको रूपमा लिनुपर्दछ ।

Supplement to: Rai GK, Saluja T, Chaudhary S, et al. Safety and immunogenicity of the Vi-DT typhoid conjugate vaccine in healthy volunteers in Nepal: an observer-blind, active-controlled, randomised, non-inferiority, phase 3 trial. *Lancet Infect Dis* 2021; published online Dec 20. https://doi.org/10.1016/S1473-3099(21)00455-2.

सारांश

पृष्ठभूमि:

कम तथा मध्यम आय भएका धेरैजसो देशहरुमा टाइफोइड ज्वरो एउटा निरन्तर भैरहने स्थानिक रोग हो । सन् २०१८ को आफ्नो अवधारणापत्र मार्फत विश्व स्वास्थ्य संगठन (WHO) ले जोखिममा रहेका समूहहरुको लागि र महामारी नियन्त्रणका लागि टाइफोइडको खोप प्रयोग गर्नेबारे देशहरूले योजना गर्नुपर्दछ भनेको छ । टाइफोइड खोपको माग र आपूर्तिको बीचको असन्तुलन लाई सम्बोधन गर्नका लागि टाइफोइड किटाणुको Vi polysaccharide अणु र diptheria toxoid अणु बीच संयोजन गरी एउटा संयोजित-खोप (Vi-DT conjugate vaccine) बनाउने पहल गरिएको थियो जसले विश्व स्वास्थ्य संगठनको गुणस्तर सम्बन्धी मापदण्ड र पूर्व योग्यता प्राप्त गरोस र टाइफोइडको संयोजित-खोप को विश्वव्यापी आपूर्तिमा टेवा पुर्याओस । यस अध्ययनको एउटा महत्वपूर्ण उद्देश्य भनेको Vi-DT conjugate vaccine लगाएपछि विभिन्न उमेर समूहका व्यक्तिहरुमा विकास हुने प्रतिरक्षात्मक प्रतिक्रिया (Immunogenicity) विश्व स्वास्थ्य संगठन बाट स्वीकृत त्यस्तै टाइफाइडको अर्को संयोजित-खोप Vi-TT (Vi polysaccharide-tetanus toxoid) conjugate vaccine (Typbar TCV; भारत बायोटेक इन्डिया, हैदराबाद, भारत) को तुलनामा कम हुँदैन (non-inferior) भनी प्रमाणित गर्नु रहेको थियो ।

विधि:

हामीले नेपालका काठमाडौँ, धुलिखेल, धरान र नेपालगञ्जका चारवटा अस्पतालहरुमा अवलोकनकर्ता अनभिज्ञ हुने गरी र सहभागिहरूको नियमितता नहुने गरी सक्रिय खोप सँग तुलना गरेर कम प्रभावकारी नभएको पुष्टि गर्न तेस्रो चरणको क्लिनिकल परीक्षण गर्यौं । अनुसन्धानका प्रक्रियाहरु पालना गर्न मन्जुर र अनुसन्धानको अवधिभर उपलब्ध हुन सक्ने छ महिना देखि पैतालिस वर्ष उमेर सम्मका स्वस्थ व्यक्तिहरु अनुसन्धानमा सहभागी हुन योग्य थिए र जसको लागि सूचित सहमति प्राप्त गरिएको थियो । अनुसन्धानको अन्तिम नतिजाको विश्लेषणमा असर पार्नसक्ने तात्कालिक वा दीर्घ प्रकृतिका बिमारी भएका व्यक्तिहरु र अन्य क्लिनिकल परीक्षणमा सहभागी भइरहेका व्यक्तिहरूलाई यस अनुसन्धानमा सहभागी गराइएको थिएन । सहभागीहरुलाई छ महिनादेखि दुई वर्ष, दुई देखि अठार वर्ष, र अठार देखि पैतालिस वर्ष को समूहमा वर्गीकरण गरी हरेक उमेर समूहका सहभागीलाई नियमितता नहुने गरी block randomization विधिबाट (ब्लक आकार ४ र ८) १: १: १: १ को अनुपातमा चार वटा समूह (A - D) मध्य कुनै एकमा राखियो । समूह A, समुह B, र समूह C का सहभागीहरुलाई असल उत्पादन प्रक्रिया बमोजिम उत्पादित Vi-DT परीक्षण खोपको तीन वटा लट हरु मध्ये क्रमशः लट १, लट २, र लट ३ को एक मात्रा खोप (२५ माइक्रोग्राम, ०.५ मिलि) मांशपेशीमा लगाइयो, भने समूह D का सहभागीहरुलाई Vi-TT खोपको को एक मात्रा (२५ माइक्रोग्राम, ०.५ मिलि) मांशपेशीमा लगाइयो । सबै सहभागीहरु, खोप लगाइदिने बाहेक अनुसन्धान स्थलका

सबै टोली सदस्यहरु, र अन्तिम नतिजा विश्लेषण गर्ने अनुसन्धान टोलीका सदस्यहरुलाई कुन समूहको खोप लगाइयो भन्ने बारेमा अनभिज्ञ राखियो । अनुसन्धानका अन्तिम विन्दु हरु निम्न थिएः (१) Vi-DT बाट उत्सर्जन हुने प्रतिरक्षात्मक प्रतिक्रिया (समूह A, समूह B, र समूह C को समस्टिगत) Vi-TT (समुह D) बाट उत्सर्जन हुने भन्दा कम नभएको कुरा खोप लगाएको ४ हप्तामा anti-Vi IgG एन्टिबडी को seroconversion (एन्टिबडीको मात्रा कम्तीमा चार गुणा वृद्धि हुने) को दरबाट मापन गर्ने; र (२) Vi-DT खोपका विभिन्न लटहरूको एकरूपता खोप लगाएको ४ हप्तामा anti-Vi IgG एन्टिबडी को भिन्नभिन्न लटहरूको जियोमेट्रिक औसत टाइटर (GMT) लाई एकआपसमा (लट १ सँग लट २, लट १ सँग लट ३, र लट २ सँग लट ३) दाँजेर प्रतिरक्षात्मक प्रतिक्रियाको समानता मार्फत पुष्टि गर्ने । Vi-DT खोप (समूह A, समूह B, र समूह C को समस्टिगत) र Vi-TT (समुह D) को seroconversion को दरको फरकको ९७.५% सुनिश्चितता अन्तराल (CI) को तल्लो विन्दु seroconversion कम नभएको देखाउने -१०% को पूर्वनिर्धारित सिमाना भन्दा माथि रहेमा Vi-DT खोप Vi-TT खोप भन्दा कमजोर नभएको कुरा पुष्टि हुने मानियो । प्रत्येक लट लाई अर्को लट संग दाँज्ने क्रममा आउने GMT को अनुपातको दुईतर्फी ९९.१७% सुनिश्चितता अन्तराल को माथिल्लो र तल्लो विन्दु विश्व स्वास्थ्य संगठन बाट सिफारिस गरिएको पूर्वनिर्धारित समानता को सिमाना का विन्दुहरू ०.६७ र १.५ को बिचमा रहेमा लटहरु बिच प्रतिरक्षात्मक प्रतिक्रियाको समानता भएको हुने मानियो । नियमितता नहुने गरी समुह विभाजन गरेर निर्धारित खोप लगाएका र आधारभूत मूल्याङ्कन पश्चात कमसेकम एकपटक प्रतिरक्षात्मक प्रतिक्रियाको मूल्यांकन भएका सबै सहभागीहरु को प्रतिरक्षात्मक प्रतिक्रियाको अन्तिम विन्दुहरूको विश्लेषण गरियो । कमसेकम एक मात्रा खोप लगाएका सबै सहभागीहरुमा खोपको प्रयोगको सुरक्षितता को मुल्यांकन गरि उमेर समूह र खोपको समूहको आधारमा व्याख्यात्मक संक्षेपीकरण गरियो । यो अनुसन्धान ClinicalTrials.gov मा क्रम संख्या NCT03933098 मा दर्ता रहेको छ ।

नतिजाहरुः

सन् २०१९ नोभेम्वर २० र २०२० मार्च १० को बिचमा १८५४ व्यक्तिहरूलाई जाँच गरी १८०० लाई नियमितता नहुनेगरी समुह A देखी समुह D (प्रत्येक समुहमा ४५० सहभागी) मा विभाजन गरियो । १७८६ (९९.२%; समुह A मा ४४३, समुह B मा ४५०, समुह C मा ४४७ र समुह D मा ४४६) सहभागीहरुलाई खोप लगाएको चार हप्तापछिको प्रतिरक्षात्मक प्रतिक्रिया को मुल्याङ्कनमा सामेल गरियो, सबै १८०० सहभागीहरुलाई खोपको सुरक्षितता सम्बन्धी मूल्याङ्कनमा सामेल गरियो । प्रतिरक्षात्मक प्रतिक्रियाको मूल्याङ्कनमा सबै उमेर समूहको समष्टिगत एन्टि-Vi-IgG seroconversion दर Vi-DT खोप समूह (समुह A-C) मा ९९.३३% (९७.५% CI ९८.६१ देखि ९९.६८; १३४० मध्य १३३१ सहभागी) थियो र Vi-TT खोप समूह (समुह D) मा

९८.८८% (९७.१० देखि ९९.५७; ४४६ मध्ये ४४१ सहभागी) थियो । Vi-DT खोप समूह (समुह A-C समस्टिगत) र Vi-TT खोप समूह (समुह D) बिच seroconversion दरको फरक ०.४७% (९७.५% CI -०.६८ देखि १.६१) थियो जसले Vi-DT खोप कमजोर नभएको कुरा पुष्टि गर्दछ । खोप लगाएको चार हप्तापछि anti-Vi IgG एन्टिबडी को जियोमेट्रिक औसत टाइटर (GMT) को अनुपात लट १ : लट २ को लागि १.०२ (९९.१७% CI ०.८५ देखि १.२२), लट १ : लट ३ को लागि १.०२ (०.८५ देखि १.२३), र लट २ : लट ३ को लागि १.०१ (०.८४ देखि १.२१) थियो जसले विश्व स्वास्थ्य संगठन बाट सिफारिस गरिएको पूर्वनिर्धारित समानता को सिमाना को आधारमा विभिन्न लट हरुमा समानता रहेको जनाउँदछ । दुष्प्रभाव (adverse effects) को जनाउ दिने सहभागीहरूको अनुपात Vi-DT खोप समूह (समुह A-C) र Vi-TT खोप समूह (समुह D) मा समान थियो; Vi-DT खोप समूह (समुह A-C) का १३५० मध्य २६० (१९.३%) तथा Vi-TT खोप समूह (समुह D) का ४५० मध्य ११५ (२५.६%) सहभागीहरुले खोप लगाएको ७ दिन भित्रमा अपेक्षित (solicited) दुष्प्रभावको जानकारी दिए, र खोप लगाए पछि ४ हप्ता भित्र Vi-DT खोप समूह (समुह A-C) का २०८ (१५.४%) तथा Vi-TT खोप समूह (समुह D) का ७६ (१६.९%) सहभागीहरुले अनपेक्षित (unsolicited) दुष्प्रभावको जानकारी दिए । २४ हप्तासम्मको फलोअपमा Vi-TT खोपमा एक मृत्यु सहित सातवटा गम्भीर दुष्प्रभावहरू (४ वटा (०.३%) Vi-DT खोप समूह A-C मा र ३ वटा (०.७%) Vi-TT खोप समूह D मा), जानकारीमा आएका थिए, ती मध्ये कुनैपनि दुष्प्रभाव परीक्षणमा प्रयोग भएका खोप हरुसँग सम्बन्धित भएको पाइएन ।

विश्लेषणः

एक मात्रा खोप दिएपछि Vi-DT परीक्षण खोप सुरक्षित रहेको पाइयो, खोप लगाएको चार हप्तामा उपयुक्त मात्रामा प्रतिरक्षात्मक प्रतिक्रिया उत्सर्जन गर्ने र त्यो Vi-TT खोप भन्दा कमजोर नरहेको पाइयो । Vi-DT खोपको तिन लटहरूको प्रतिरक्षात्मक प्रतिक्रियामा समानता समेत पुष्टि भयो जसले खोपको उत्पादन प्रक्रिया सही भएको जनाउँदछ । विश्व स्वास्थ्य संगठन बाट गुणस्तर सम्बन्धी पूर्व योग्यता स्थापित भएपश्चात यो खोप संयुक्त राष्ट्रसङ्घका विभिन्न निकायहरुलाई खरिदको लागि एक विकल्प हुनसक्छ ।

अनुवादक (Translator): डा. विष्णु रथ गिरी
