# Supplementary material for: Safety and immunogenicity of the Vi-DT typhoid conjugate vaccine in healthy volunteers in Nepal: an observer-blind, active-controlled, randomised, non-inferiority, phase 3 trial
Source: Lancet Infect Dis. 2022 Apr;22(4):529–40. doi: 10.1016/S1473-3099(21)00455-2 (PMC8942857; doi:10.1016/S1473-3099(21)00455-2)
Supplement: Supplementary appendix 2 [file mmc2.pdf]

THE LANCET
Infectious Diseases

Supplementary appendix 2

This appendix formed part of the original submission and has been peer reviewed. We post it as supplied by the authors.

Supplement to: Rai GK, Saluja T, Chaudhary S, et al. Safety and immunogenicity of the Vi-DT typhoid conjugate vaccine in healthy volunteers in Nepal: an observer-blind, active-controlled, randomised, non-inferiority, phase 3 trial. *Lancet Infect Dis* 2021; published online Dec 20. https://doi.org/10.1016/S1473-3099(21)00455-2.

Supplementary Appendix

Supplementary Table S1: Inclusion & Exclusion Criteria Page 3

Supplementary Table S2: Schedule of Events Page 5

Supplementary Table S3: Secondary outcomes Page 7

Supplementary Method: In-house anti-Vi IgG ELISA Page 8

Supplementary Table S4: Non-inferiority on Seroconversion of Anti-Vi IgG ELISA Response at Week 4 – PP set Page 9

Supplementary Table S5: Lot to lot consistency on GMT of Anti-Vi IgG ELISA Response at Week 4 – PP set Page 10

Supplementary Table S6: Non-inferiority on GMT of Anti-Vi IgG ELISA response at week 4 – Immunogenicity set Page 11

Supplementary Figure S1: Non-inferiority on GMT of Anti-Vi IgG ELISA response at week 4 – Immunogenicity set Page 12

Supplementary Table S7: Non-inferiority on GMT of Anti-Vi IgG ELISA Response at Week 4 – PP set Page 13

Supplementary Figure S2: Failure rate (%) to seroconversion of Anti-Vi IgG ELISA response at week 4 – Immunogenicity set Page 14

Supplementary Table S8: Lot to lot consistency on Seroconversion of Anti-Vi IgG ELISA Response at Week 4 – PP set Page 15

Supplementary Table S9: Non-interference between Vi-DT/Vi-TT and MR vaccine at Week 4 – Immunogenicity set Page 17

Supplementary Table S10: Seroconversion rate of Anti-Vi IgG ELISA Response at Week 24 Weeks -Immunogenicity set Page 18

Supplementary Table S11: Seroconversion rate of Anti-Vi IgG ELISA Response at Week 24 – PP set Page 19

Supplementary Table S12: GMT of Anti-Vi IgG ELISA Response at Week 24 Immunogenicity set Page 20

Supplementary Table S13: GMT of Anti-Vi IgG ELISA Response at Week 24 – PP set Page 21

Supplementary Table S14: Summary of Adverse Events Page 22

Supplementary Table S16: Distribution of immediate reactions Page 24

Supplementary Table S17: Distribution of solicited AEs Page 26

Supplementary Table S18: Distribution of unsolicited AEs within 4 weeks after vaccination Page 28

Supplementary Table S15: Summary of Major Protocol Deviations Page 31

Supplementary Table S1: Inclusion & Exclusion Criteria

**Inclusion Criteria**
Healthy participants 6 months to 45 years of age at enrolment,
Healthy participants 9 to 15 months of age at enrolment (additional group only),
Participants/parents/LAR who have voluntarily given written informed consent/assent,
Participants/parents/LAR willing to follow the study procedures of the study and available for the entire duration of the study.
**Exclusion Criteria**
Child with a congenital abnormality,
Subject concomitantly enrolled or scheduled to be enrolled in another trial,
Known history of immune function disorders including immunodeficiency diseases (Known HIV infection or other immune function disorders),
Chronic use of systemic steroids (>2 mg/kg/day or >20 mg/day prednisone equivalent for periods exceeding 10 days), cytotoxic or other immunosuppressive drugs,
Receipt of blood or blood-derived products in the past 3 months,
Subject with a previously ascertained or suspected disease caused by *S*. Typhi,
Subject who has had household contact with/and or intimate exposure to an individual with laboratory-confirmed *S*. Typhi,
Individual who has previously received a typhoid vaccine,
Subject who has received or has been expected to receive other vaccines from 1 month prior to IP vaccination to Visit 4 (approx. 1 month post-IP) except PCV booster as per EPI schedule or any vaccine during National Immunization catch-up campaign of Nepal,
Known history or allergy to vaccines or other medications,
History of uncontrolled coagulopathy or blood disorders,

Any abnormality or chronic disease which in the opinion of the investigator might be detrimental for the safety of the subject and interfere with the assessment of the study objectives,
Any female participant who is lactating, pregnant or planning for pregnancy during the study period*,
Participants/parents/LAR planning to move from the study area before the end of study period,
As per Investigator’s medical judgement, despite meeting all inclusion/exclusion criteria mentioned above.
**Temporary Contra-indication**
Acute illness, in particular infectious disease, or fever (axillary temperature ≥37.5°C), within three days prior to enrollment and vaccination.These individuals could be rescreened upon resolution of the above said conditions.
*Urine pregnancy test (UPT) was performed in all married females prior to vaccine administration.

Supplementary Table S2: Schedule of Events

Visit Number	V1	V2	V3	V4	V5	V6
Visit Day	D-7 - 0	D0	D7	D28	D84	D168
Visit Week	-1	0	1	4	12	24
Visit Window[#]		+1D	±1D	±3D	±7D	±7D
Informed Consent / assent	X					
Screening	X					
Eligibility Criteria	X					
Informed Consent / assent	X					
Inclusion & Exclusion Criteria	X	X				
Medical History	X	X				
Vital Signs	X	X	X	X	X	X
Physical Examination	X	X	X	X	X	X
Enrollment & Randomization		X				
Vi-DT or Typbar TCV® Vaccination		X				
Post Vaccination 30 min F/U		X				
Solicited AE		X	X			
Unsolicited AE		X	X	X		
SAE		X	X	X	X	X
Diary Card Provided		X (DC1)	X(DC2)	X(DC3)		
Diary Card Collected			X(DC1)	X(DC2)		X(DC3)
Concomitant Medications	X	X	X	X	X*	X*

**For < 2years Immunogenicity Blood Volume (approx.)**		**3mL**		**3mL**		**3mL**
**For < 2years Cumulative Blood Volume (approx.)**		**3 mL**		**6 mL**		**9 mL**
**For ≥ 2years Immunogenicity Blood Volume (approx.)**		**5mL**		**5mL**		**5mL**
**For ≥ 2years Cumulative Blood Volume(approx.)**		**5 mL**		**10 mL**		**15 mL**

Supplementary Table S3: Secondary Endpoint/s Comparisons

- Geometric Mean Titers (GMT) of anti-Vi IgG at D28 (4 weeks post vaccination) of Vi-DT (Groups A+B+C) is non-inferior to Typbar TCV® (Group D) using non-inferiority margin of GMT ratio > 0.67,
- Anti-Vi IgG seroconversion rate at D28 post each lot of Vi-DT vaccine is equivalent to each other (A vs. B, B vs. C, and C vs. A) using equivalence margin of difference of [-10%, 10%],
- Sero-positive rate of IgG antibody titers for Measles (M), Mumps (M) and Rubella (R) following single dose of MMR at 4 weeks in participants who received MMR + Vi-DT is non-inferior to sero-positive rate in participants who received MMR alone with non-inferiority margin of -10% (additional group only),

The following secondary endpoints to be described by vaccine groups as well as by age strata:

- Geometric Mean Titers (GMT) of anti-Vi IgG at D168 (24 weeks post vaccination) of Vi-DT (Groups A+B+C) and Typbar TCV® (Group D),
- Anti-Vi IgG seroconversion rate at D168 post Vi-DT vaccination (25 µg) (Groups A+B+C) and Typbar TCV® (Group D),
- Anti-Vi IgG seroconversion rate and GMT at 4 weeks (day 28) post Vi-DT vaccine (25 µg) (pooled Groups A+B+C) and Typbar TCV® (Group D) in participants who received concomitantly MR vaccine,
- Anti-Vi IgG seroconversion rate and GMT at 4 weeks (day 28) post Vi-DT vaccine (25 µg) (pooled Groups A+B+C) in participants 9-15 months who concomitantly received and did not receive MR vaccine,
- Sero-positive rate of IgG antibody titers for Measles (M) and Rubella (R) at 4 weeks (day 28) post vaccination of Vi-DT (Groups A+B+C combined) and Typbar TCV® (Group D) in participants who received concomitant MR vaccine,
- Frequency of local and systemic solicited adverse events during the 7 days after vaccination
    - Solicited local reactions at the site of injection: Pain/ tenderness, erythema/redness, swelling/ induration, pruritus pruritus associated with injection
    - Solicited Systemic reactions (adapted to each age group): Fever, headache,fatigue, myalgia,lethargy, irritability, nausea, vomiting, arthralgia, diarrhea, drowsiness, loss of appetite, chills, rash,nasopharyngitis and persistent crying
- Frequency of unsolicited adverse events during 4 weeks after vaccination
- Frequency of Serious Adverse Events during the entire study period

Supplementary Method: In-house anti-Vi IgG ELISA

The assay was used to assess anti-Vi IgG antibodies in human sera. Briefly, 96-well plates (Nunc-Immunoplates, MaxisorpTM, USA) were precoated with 10 µg/ml poly-L-lysine (Sigma-Aldrich, USA) for 2h. After washing the plates, 2 µg/ml Vi antigen (SK Bioscience, South Korea) was coated overnight at 37˚C. The plates were blocked with 1% bovine serum albumin in PBS for 1h and then, diluted human sera and reference serum were added to the plates accordingly and 2-fold serially diluted. After incubation of the plates for 1 h at 37˚C and washing, alkaline phosphatase (AP)-conjugated mouse anti-human IgG (Abcam, UK) was added and incubated for 1 h. After washing, 4-nitrophenyl phosphate substrate (Sigma-Aldrich) was added for 1 h. The plate was read at 405 nm and corrected at 490 nm. Anti-Vi IgG titer was determined as international unit per ml based on NIBSC international standard serum (NIBSC 16/138).

Supplementary Table S4: Non-inferiority on Seroconversion of Anti-Vi IgG ELISA Response at Week 4 – PP set

Week 4 Post Vaccination	Vi-DT Group (Pooled Groups A+B+C)		Vi-TT Group		Vi-DT - Vi-TT	P-value‡
	n/N	Seroconversion rate * (97.5% CI)	n/N	Seroconversion rate* (97.5% CI)	Difference (97.5% CI)†	
**All Ages**	1316/1325	99.32 (98.60, 99.67)	437/442	98.87 (97.07, 99.57)	0.47 (-0.69, 1.62)	0.3633
**Age Strata1**	424/429	98.83 (97.30, 99.50)	144/146	98.63 (95.14, 99.62)	0.20 (-2.83, 3.24)	1.0000
**Age Strata2**	447/448	99.78 (98.75, 99.96)	147/148	99.32 (96.27, 99.88)	0.45 (-2.42, 3.32)	0.4353
**Age Strata3**	445/448	99.33 (98.05, 99.77)	146/148	98.65 (95.21, 99.63)	0.68 (-2.28, 3.65)	0.6023

[Note] N: number of total participants; n: number of participants seroconverted.

* Proportion of participants who had at least 4-fold rise anti-Vi antibody titers compared to baseline.

† The non-inferiority of Vi-DT was confirmed as the lower limit of two tailed 95% confidence interval of the difference of seroconversion rate of Vi-DT and Typbar TCV was greater than the non-inferiority margin of -10%.

‡ The p-value was derived from Fisher's exact test.

Supplementary Table S5: Lot to lot consistency on GMT of Anti-Vi IgG ELISA Response at Week 4 – PP set

All Ages	Comparison Group1		Comparison Group 2		Group 1 / Group 2	P-value‡
	N	GMT* (99.17% CI)	N	GMT* (99.17% CI)	Ratio (99.17% CI) †	
Lot 1 vs Lot 2	441	453.22 (397.90, 516.23)	445	445.42 (391.51, 506.74)	1.018 (0.849, 1.221)	0.7951
Lot 1 vs Lot 3	441	453.22 (397.90, 516.23)	439	445.20 (390.70, 507.31)	1.017 (0.847, 1.222)	0.8032
Lot 2 vs Lot 3	445	445.42 (391.51, 506.74)	439	445.20 (390.70, 507.31)	1.000 (0.833, 1.200)	0.9961
**Age Strata1**	**Comparison Group1**		**Comparison Group 2**		**Group 1 / Group 2**	**P-value‡**
	**N**	**GMT* (95% CI)**	**N**	**GMT* (95% CI)**	**Ratio (95% CI)†**	
Lot 1 vs Lot 2	143	552.44 (482.77, 632.16)	145	538.36 (482.28, 600.97)	1.03 (0.86, 1.22)	0.7696
Lot 1 vs Lot 3	143	552.44 (482.77, 632.16)	141	466.96 (400.28, 544.75)	1.18 (0.97, 1.45)	0.1057
Lot 2 vs Lot 3	145	538.36 (482.28, 600.97)	141	466.96 (400.28, 544.75)	1.15 (0.95, 1.39)	0.1386
**Age Strata2**	**Comparison Group1**		**Comparison Group 2**		**Group 1 / Group 2**	**P-value‡**
	**N**	**GMT* (95% CI)**	**N**	**GMT* (95% CI)**	**Ratio (95% CI)†**	
Lot 1 vs Lot 2	149	410.48 (352.60, 477.87)	150	402.84 (350.68, 462.77)	1.02 (0.83, 1.25)	0.8570
Lot 1 vs Lot 3	149	410.48 (352.60, 477.87)	149	445.14 (380.55, 520.70)	0.92 (0.74, 1.15)	0.4638
Lot 2 vs Lot 3	150	402.84 (350.68, 462.77)	149	445.14 (380.55, 520.70)	0.90 (0.73, 1.11)	0.3467
**Age Strata3**	**Comparison Group1**		**Comparison Group 2**		**Group 1 / Group 2**	**P-value‡**
	**N**	**GMT* (95% CI)**	**N**	**GMT* (95% CI)**	**Ratio (95% CI)†**	
Lot 1 vs Lot 2	149	413.82 (337.54, 507.34)	150	410.05 (328.03, 512.57)	1.01 (0.75, 1.36)	0.9522
Lot 1 vs Lot 3	149	413.82 (337.54, 507.34)	149	425.60 (351.43, 515.43)	0.97 (0.74, 1.28)	0.8429
Lot 2 vs Lot 3	150	410.05 (328.03, 512.57)	149	425.60 (351.43, 515.43)	0.96 (0.72, 1.29)	0.8026

[Note] N: number of total participants; * Geometric Mean Titers (unit: IU/ml)
† The equivalence of two lots was confirmed as the both limits of two-tailed 95% confidence interval of the fold difference of GMT between two lots of Vi-DT was within the equivalence margin of [0.67, 1.5].
‡ The p-value was derived from two sample t-test.

Supplementary Table S6: Non-inferiority on GMT of Anti-Vi IgG ELISA response at week 4 – Immunogenicity set

Week 4 Post Vaccination	Vi-DT Group (Pooled Groups A+B+C)		Vi-TT Group		Vi-DT Group / Vi-TT Group	
	N	GMT* (95% CI)	N	GMT* (95% CI)	Ratio (95% CI) †	
**All Ages**	1340	444.98 (421.12, 470.20)	446	381.19 (348.23, 417.27)	1.169 (1.049, 1.302)	
**Age Strata1**	443	505.39 (468.07, 545.68)	150	421.74 (354.69, 501.45)	1.20 (0.99, 1.45)	
**Age Strata2**	448	419.06 (384.66, 456.52)	148	450.74 (396.56, 512.33)	0.93 (0.80, 1.08)	
**Age Strata3**	449	416.70 (370.27, 468.94)	148	290.98 (248.21, 341.11)	1.43 (1.18, 1.74)	

* Geometric Mean Titers (unit: IU/ml)

† The non-inferiority of Vi-DT will be confirmed if the lower limit of two-tailed 95% confidence interval of the ratio of GMT of Vi-DT to Vi-TT is greater than the non-inferiority margin of 0.67.

Supplementary Figure S1: **Box-whisker plot of Anti-Vi IgG ELISA response at week 4 – Immunogenicity set**

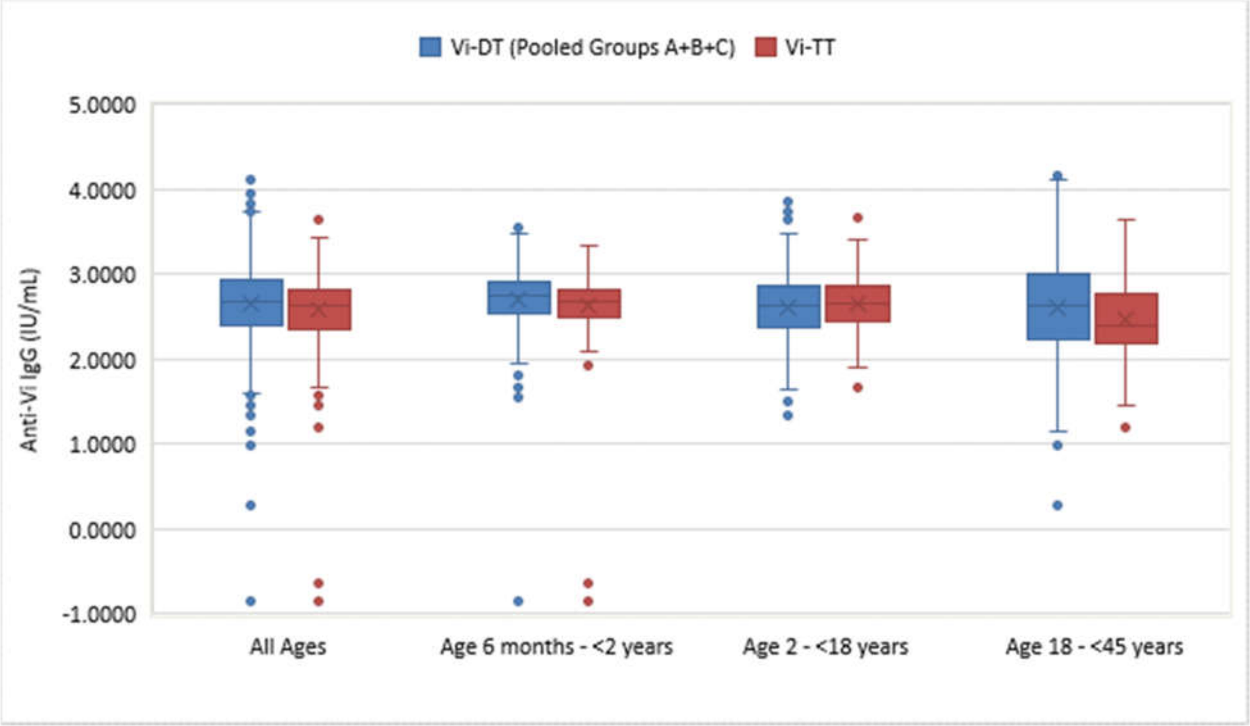

Supplementary Table S7: Non-inferiority on GMT of Anti-Vi IgG ELISA Response at Week 4 – PP set

Week 4 Post Vaccination	Vi-DT Group (Pooled Groups A+B+C)		Vi-TT Group		Vi-DT Group / Vi-TT Group	P-value‡
	N	GMT* (95% CI)	N	GMT* (95% CI)	Ratio (95% CI) †	
**All Ages**	1325	447.93 (423.76, 473.47)	442	384.22 (350.85, 420.76)	1.167 (1.047, 1.302)	0.0053
**Age Strata1**	429	518.20 (479.83, 559.65)	146	433.16 (363.39, 516.32)	1.20 (0.99, 1.45)	0.0663
**Age Strata2**	448	419.06 (384.66, 456.52)	148	450.74 (396.56, 512.33)	0.93 (0.80, 1.08)	0.3514
**Age Strata3**	448	416.42 (369.93, 468.76)	148	290.98 (248.21, 341.11)	1.43 (1.17, 1.74)	0.0004

* Geometric Mean Titers (unit: IU/ml)

† The non-inferiority of Vi-DT was confirmed as the lower limit of two-tailed 95% confidence interval of the ratio of GMT of Vi-DT to Vi-TT was greater than the non-inferiority margin of 0.67.

‡ The p-value was derived from two sample t-test.

Supplementary Figure S2: Failure rate (%) to seroconversion of Anti-Vi IgG ELISA response at week 4 – Immunogenicity set

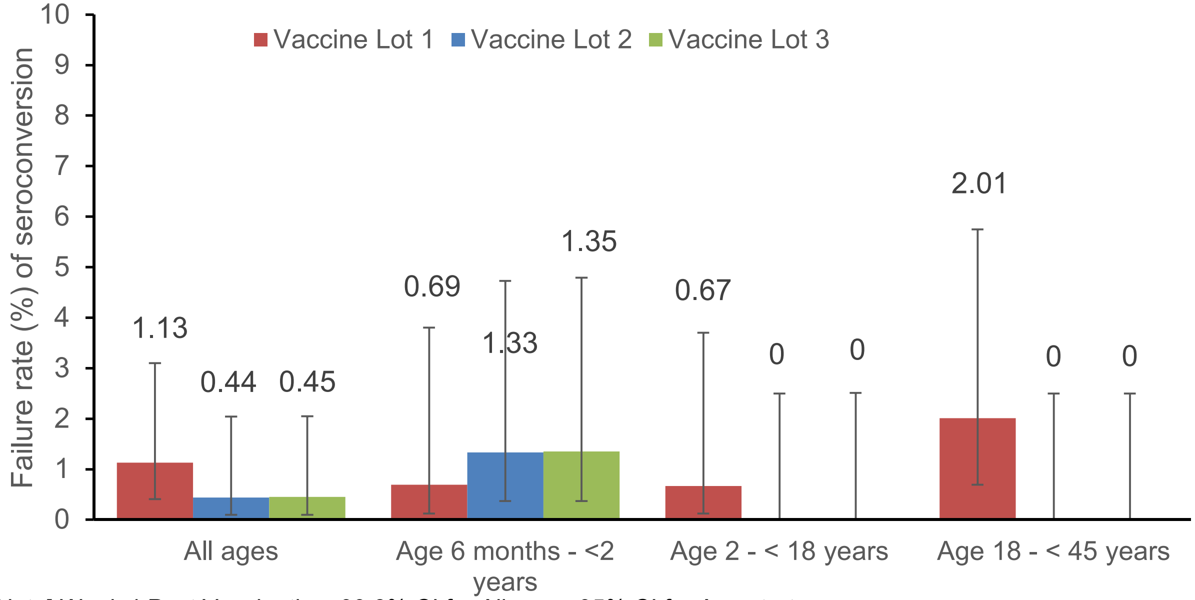

[Note] Week 4 Post Vaccination. 99.8% CI for All ages. 95% CI for Age strata.

Supplementary Table S8: Lot to lot consistency on Seroconversion of Anti-Vi IgG ELISA Response at Week 4 – PP set

All Ages	Comparison Group 1		Comparison Group 2		Group 1 - Group 2	P-value‡
	n/N	Seroconversion rate* (98.33% CI)	n/N	Seroconversion rate* (98.33% CI)	Difference (98.33% CI)†	
Lot 1 vs Lot 2	436/441	98.87 (96.89, 99.59)	443/445	99.55 (97.94, 99.90)	-0.68 (-2.11, 0.74)	0.2501
Lot 1 vs Lot 3	436/441	98.87 (96.89, 99.59)	437/439	99.54 (97.91, 99.90)	-0.68 (-2.11, 0.76)	0.2589
Lot 2 vs Lot 3	443/445	99.55 (97.94, 99.90)	437/439	99.54 (97.91, 99.90)	0.01 (-1.07, 1.10)	0.9775
**Age Strata1**	**Comparison Group 1**		**Comparison Group 2**		**Group 1 - Group 2**	**P-value‡**
	**n/N**	**Seroconversion rate* (95% CI)**	**n/N**	**Seroconversion rate* (95% CI)**	**Difference (95% CI)†**	
Lot 1 vs Lot 2	142/143	99.30 (96.15, 99.88)	143/145	98.62 (95.11, 99.62)	0.68 (-4.56, 5.92)	1.0000
Lot 1 vs Lot 3	142/143	99.30 (96.15, 99.88)	139/141	98.58 (94.98, 99.61)	0.72 (-4.53, 5.96)	0.6210
Lot 2 vs Lot 3	143/145	98.62 (95.11, 99.62)	139/141	98.58 (94.98, 99.61)	0.04 (-5.25, 5.32)	1.0000
**Age Strata2**	**Comparison Group 1**		**Comparison Group 2**		**Group 1 - Group 2**	**P-value‡**
	**n/N**	**Seroconversion rate* (95% CI)**	**n/N**	**Seroconversion rate* (95% CI)**	**Difference (95% CI)†**	
Lot 1 vs Lot 2	148/149	99.33 (96.30, 99.88)	150/150	100.00 (97.50, 100.00)	-0.67 (-5.62, 4.28)	0.4983
Lot 1 vs Lot 3	148/149	99.33 (96.30, 99.88)	149/149	100.00 (97.49, 100.00)	-0.67 (-5.64, 4.30)	1.0000
Lot 2 vs Lot 3	150/150	100.00 (97.50, 100.00)	149/149	100.00 (97.49, 100.00)	0.00 (0.00, 0.00)	NA
**Age Strata**	**Comparison Group 1**		**Comparison Group 2**		**Group 1 - Group 2**	**P-value‡**
	**n/N**	**Seroconversion rate* (95% CI)**	**n/N**	**Seroconversion rate* (95% CI)**	**Difference (95% CI)†**	
Lot 1 vs Lot 2	146/149	97.99 (94.25, 99.31)	150/150	100.00 (97.50, 100.00)	-2.01 (-7.25, 3.22)	0.1225
Lot 1 vs Lot 3	146/149	97.99 (94.25, 99.31)	149/149	100.00 (97.49, 100.00)	-2.01 (-7.27, 3.24)	0.2475
Lot 2 vs Lot 3	150/150	100.00 (97.50, 100.00)	149/149	100.00 (97.49, 100.00)	0.00 (0.00, 0.00)	NA

[Note] N: number of total participants; n: number of participants seroconverted. NA: Not Applicable.
* Proportion of participants who had at least 4-fold rise anti-Vi antibody titers compared to baseline.

† The equivalence of two lots will be confirmed if the both limits of two-tailed 95% confidence interval of the difference of seroconversion rate between two lots of Vi-DT is within the equivalence margin of [-10%, 10%].
‡ The p-value was derived from Fisher's exact test.

Supplementary Table S9: Non-interference between Vi-DT/Vi-TT and MR vaccine at Week 4 – Immunogenicity set

9-15 months old	Vi-DT + MR Group (Pooled Groups A+B+C) (N=77)		Vi-TT + MR Group (N=30)		P-value‡
	n	Estimate (95% CI)	n	Estimate (95% CI)	
**Anti-Vi Seroconversion rate***	77	100.00 (95.25, 100.00)	30	100.00 (88.65, 100.00)	NA
**Anti-Vi GMT†**	-	501.50 (423.11, 594.41)	-	464.38 (366.80, 587.92)	0.5939

[Note] N: number of total participants; n: number of particiants seroconverted. NA: Not Applicable.

* Proportion of participants who had at least 4-fold rise anti-Vi antibody titers compared to baseline

† Anti Vi IgG Geometric Mean Titers (unit: IU/ml)

‡ The p-value for GMT comparison was derived from two sample t-test.

Supplementary Table S10: Seroconversion rate of Anti-Vi IgG ELISA Response at Week 24 Weeks - Immunogenicity set

Week 24 Post Vaccination	Vi-DT Group (Pooled Groups A+B+C)		Vi-TT Group		P-value†
	n/N	Seroconversion rate* (95% CI)	n/N	Seroconversion rate* (95% CI)	
**All Ages**	1213/1231	98.54 (97.70, 99.07)	391/402	97.26 (95.17, 98.47)	0.2338
**Age Strata1**	367/372	98.66 (96.89, 99.42)	126/126	100.00 (97.04, 100.00)	0.3366
**Age Strata2**	435/438	99.32 (98.01, 99.77)	140/144	97.22 (93.08, 98.91)	0.0670
**Age Strata3**	411/421	97.62 (95.68, 98.70)	125/132	94.70 (89.46, 97.41)	0.1425

[Note] N: number of total participants; n: number of participants seroconverted.

* Proportion of participants who had at least 4-fold rise of anti-Vi IgG antibody titers compared to baseline.

† The p-value for all ages was derived using the generalized linear model for binomial distribution with group and strata as covariates. The p-value for each age strata was derived from Fisher's exact test.

Supplementary Table S11: Seroconversion rate of Anti-Vi IgG ELISA Response at Week 24 – PP set

Week 24 Post Vaccination	Vi-DT Group (Pooled Groups A+B+C)		Vi-TT Group		P-value†
	n/N	Seroconversion rate* (95% CI)	n/N	Seroconversion rate* (95% CI)	
**All Ages**	1136/1152	98.61 (97.76, 99.14)	370/380	97.37 (95.22, 98.56)	0.3132
**Age Strata1**	345/350	98.57 (96.70, 99.39)	119/119	100.00 (96.87, 100.00)	0.3361
**Age Strata2**	407/410	99.27 (97.87, 99.75)	133/136	97.79 (93.72, 99.25)	0.1669
**Age Strata3**	384/392	97.96 (96.03, 98.96)	118/125	94.40 (88.89, 97.26)	0.0603

[Note] N: number of total participants; n: number of participants seroconverted.

* Proportion of participants who had at least 4-fold rise of anti-Vi IgG antibody titers compared to baseline.

† The p-value for all ages was derived using the generalized linear model for binomial distribution with group and strata as covariates. The p-value for each age strata was derived from Fisher’s exact test

Supplementary Table S12: GMT of Anti-Vi IgG ELISA Response at Week 24 Immunogenicity set

Week 24 Post Vaccination	Vi-DT Group (Pooled Groups A+B+C)		Vi-TT Group		P-value†
	N	GMT* (95% CI)	N	GMT* (95% CI)	
**All Ages**	1231	88.07 (83.04, 93.41)	402	99.31 (91.07, 108.29)	0.0168
**Age Strata1**	372	50.17 (47.07, 53.48)	126	73.41 (65.52, 82.26)	<0.0001
**Age Strata2**	438	92.87 (85.01, 101.44)	144	110.03 (94.30, 128.39)	0.0609
**Age Strata3**	421	137.03 (121.87, 154.07)	132	118.47 (100.61, 139.51)	0.1544

* Geometric Mean Titers (unit: IU/ml)

† The p-value for all ages was derived using an analysis of covariance model with group and age strata as covariates after log transformation. The p-value for each age strata was derived from two sample t-test after log transformation.

Supplementary Table S13: GMT of Anti-Vi IgG ELISA Response at Week 24 – PP set

Week 24 Post Vaccination	Vi-DT Group (Pooled Groups A+B+C)		Vi-TT Group		P-value†
	N	GMT* (95% CI)	N	GMT* (95% CI)	
All Ages	1152	90.28 (84.95, 95.94)	380	100.87 (92.40, 110.12)	0.0320
Age Stratum 1	350	51.03 (47.81, 54.46)	119	75.09 (66.96, 84.21)	<0.0001
Age Stratum 2	410	94.80 (86.59, 103.78)	136	112.03 (96.11, 130.59)	0.0651
Age Stratum 3	392	142.77 (126.42, 161.23)	125	119.18 (100.59, 141.21)	0.0887

* Geometric Mean Titers (unit: IU/ml)

† The p-value for all ages was derived using an analysis of covariance model with group and age strata as covariates after log transformation. The p-value for each age strata was derived from two sample t-test after log transformation.

Supplementary Table S14: Summary of Adverse Events

[All ages] Entire study period (day 0 to day 28)		Vi-DT Group (N=1350)		Lot 1 (N=450)		Lot 2 (N=450)		Lot 3 (N=450)		Vi-TT Group (N=450)		P-value
		m	n (%)	m	n (%)	m	n (%)	m	n (%)	m	n (%)	
**Immediate Reaction (for 30 minutes after vaccination)**		**16**	**16 (1.19%)**	**3**	**3 (0.67%)**	**5**	**5 (1.11%)**	**8**	**8 (1.78%)**	**4**	**4 (0.89%)**	**0.6036[1] 0.4183[2]**
Severity:	Mild	14	14 (1.04%)	3	3 (0.67%)	4	4 (0.89%)	7	7 (1.56%)	3	3 (0.67%)	0.5859[3] 0.5026[4]
	Moderate	2	2 (0.15%)	0	0 (0.00%)	1	1 (0.22%)	1	1 (0.22%)	1	1 (0.22%)	1.0000[3] 1.0000[4]
	Severe	0	0 (0.00%)	0	0 (0.00%)	0	0 (0.00%)	0	0 (0.00%)	0	0 (0.00%)	NA
	Potentially life threatening	0	0 (0.00%)	0	0 (0.00%)	0	0 (0.00%)	0	0 (0.00%)	0	0 (0.00%)	NA
Relatedness:	Definitely Related	10	10 (0.74%)	3	3 (0.67%)	2	2 (0.44%)	5	5 (1.11%)	3	3 (0.67%)	1.0000[3] 0.7653[4]
	Probably Related	3	3 (0.22%)	0	0 (0.00%)	2	2 (0.44%)	1	1 (0.22%)	0	0 (0.00%)	0.5777[3] 0.6244[4]
	Possibly related	3	3 (0.22%)	0	0 (0.00%)	1	1 (0.22%)	2	2 (0.44%)	1	1 (0.22%)	1.0000[3] 0.9059[4]
	Unlikely related	0	0 (0.00%)	0	0 (0.00%)	0	0 (0.00%)	0	0 (0.00%)	0	0 (0.00%)	NA
	Not Related	0	0 (0.00%)	0	0 (0.00%)	0	0 (0.00%)	0	0 (0.00%)	0	0 (0.00%)	NA
**Solicited AE (during 7 days after vaccination)**		**453**	**260 (19.26%)**	**137**	**81 (18.00%)**	**141**	**91 (20.22%)**	**175**	**88 (19.56%)**	**191**	**115 (25.56%)**	**0.0044[1] 0.0318[2]**
Severity:	Mild	386	247 (18.30%)	118	75 (16.67%)	131	89 (19.78%)	137	83 (18.44%)	166	107 (23.78%)	0.0113[1] 0.0502[2]
	Moderate	59	34 (2.52%)	17	11 (2.44%)	8	7 (1.56%)	34	16 (3.56%)	22	15 (3.33%)	0.3576[1] 0.2348[2]
	Severe	7	5 (0.37%)	2	2 (0.44%)	1	1 (0.22%)	4	2 (0.44%)	3	2 (0.44%)	1.0000[3] 1.0000[4]
	Potentially life threatening	1	1 (0.07%)	0	0 (0.00%)	1	1 (0.22%)	0	0 (0.00%)	0	0 (0.00%)	1.0000[3] 1.0000[4]
Relatedness:	Definitely Related	190	153 (11.33%)	50	44 (9.78%)	63	55 (12.22%)	77	54 (12.00%)	90	79 (17.56%)	0.0006[1] 0.0044[2]
	Probably Related	70	50 (3.70%)	22	15 (3.33%)	23	17 (3.78%)	25	18 (4.00%)	29	14 (3.11%)	0.5566[1] 0.8854[2]
	Possibly related	166	96 (7.11%)	53	33 (7.33%)	51	30 (6.67%)	62	33 (7.33%)	66	36 (8.00%)	0.5310[1] 0.8990[2]
	Unlikely related	21	8 (0.59%)	8	4 (0.89%)	4	3 (0.67%)	9	1 (0.22%)	5	2 (0.44%)	1.0000[3] 0.7179[4]
	Not Related	6	4 (0.30%)	4	2 (0.44%)	0	0 (0.00%)	2	2 (0.44%)	1	1 (0.22%)	1.0000[3] 0.7648[4]

**Unsolicited AE (during 4 weeks after vaccination)**		**361**	**208 (15.41%)**	**110**	**63 (14.00%)**	**135**	**74 (16.44%)**	**116**	**71 (15.78%)**	**143**	**76 (16.89%)**	**0.4553[1] 0.6506[2]**
Severity:	Mild	300	183 (13.56%)	84	54 (12.00%)	117	68 (15.11%)	99	61 (13.56%)	121	70 (15.56%)	0.2904[1] 0.4041[2]
	Moderate	56	34 (2.52%)	21	11 (2.44%)	18	10 (2.22%)	17	13 (2.89%)	22	13 (2.89%)	0.6696[1] 0.8987[2]
	Severe	5	2 (0.15%)	5	2 (0.44%)	0	0 (0.00%)	0	0 (0.00%)	0	0 (0.00%)	1.0000[3] 0.2496[4]
	Potentially life threatening	0	0 (0.00%)	0	0 (0.00%)	0	0 (0.00%)	0	0 (0.00%)	0	0 (0.00%)	NA
Relatedness:	Definitely Related	0	0 (0.00%)	0	0 (0.00%)	0	0 (0.00%)	0	0 (0.00%)	0	0 (0.00%)	NA
	Probably Related	0	0 (0.00%)	0	0 (0.00%)	0	0 (0.00%)	0	0 (0.00%)	0	0 (0.00%)	NA
	Possibly related	0	0 (0.00%)	0	0 (0.00%)	0	0 (0.00%)	0	0 (0.00%)	0	0 (0.00%)	NA
	Unlikely related	152	93 (6.89%)	44	28 (6.22%)	58	32 (7.11%)	50	33 (7.33%)	79	41 (9.11%)	0.1199[1] 0.4120[2]
	Not Related	209	116 (8.59%)	66	35 (7.78%)	77	43 (9.56%)	66	38 (8.44%)	64	37 (8.22%)	0.8072[1] 0.8030[2]
**SAE**		**4**	**4 (0.30%)**	**2**	**2 (0.44%)**	**1**	**1 (0.22%)**	**1**	**1 (0.22%)**	**1**	**1 (0.22%)**	**1.0000[3] 1.0000[4]**

[Note] N: number of total participants; n: number of participants who reported events; m: number of events; %: percentages (100*n/N). NA: Not Applicable
The p-value was derived from [1] Chi-square test or [3] Fisher’s exact test compared between Vi-DT (Pooled Lot1+Lot2_Lot3) and Vi-TT.
The p-value was derived from [2] Chi-square test or [4] Fisher’s exact test compared among Vi-DT Lot1, Lot2, Lot3, and Vi-TT.

Supplementary Table S16: Distribution of immediate reactions

[All ages] Immediate reactions	Vi-DT Group (N=1350)		Lot 1 (N=450)		Lot 2 (N=450)		Lot 3 (N=450)		Vi-TT Group (N=450)		P-value
	m	n (%)	m	n (%)	m	n (%)	m	n (%)	m	n (%)	
**All ages**	**16**	**16 (1.19%)**	**3**	**3 (0.67%)**	**5**	**5 (1.11%)**	**8**	**8 (1.78%)**	**4**	**4 (0.89%)**	**0.6036[1] 0.4183[2]**
**Local AE**	**14**	**14 (1.04%)**	**3**	**3 (0.67%)**	**4**	**4 (0.89%)**	**7**	**7 (1.56%)**	**4**	**4 (0.89%)**	**1.0000[3] 0.6760[4]**
Pain/Tenderness	11	11 (0.81%)	2	2 (0.44%)	3	3 (0.67%)	6	6 (1.33%)	3	3 (0.67%)	1.0000[3] 0.5802[4]
Erythema/Redness	0	0 (0.00%)	0	0 (0.00%)	0	0 (0.00%)	0	0 (0.00%)	1	1 (0.22%)	0.2500[3] 1.0000[4]
Swelling/Induration	0	0 (0.00%)	0	0 (0.00%)	0	0 (0.00%)	0	0 (0.00%)	0	0 (0.00%)	NA
Pruritus	0	0 (0.00%)	0	0 (0.00%)	0	0 (0.00%)	0	0 (0.00%)	0	0 (0.00%)	NA
Pain/Tenderness (MR) *	0	0 (0.00%)	0	0 (0.00%)	0	0 (0.00%)	0	0 (0.00%)	0	0 (0.00%)	NA
Erythema/Redness (MR) *	3	3 (3.85%)	1	1 (4.17%)	1	1 (3.57%)	1	1 (3.85%)	0	0 (0.00%)	0.5587[3] 0.7026[4]
Swelling/Induration (MR) *	0	0 (0.00%)	0	0 (0.00%)	0	0 (0.00%)	0	0 (0.00%)	0	0 (0.00%)	NA
Pruritus (MR)	0	0 (0.00%)	0	0 (0.00%)	0	0 (0.00%)	0	0 (0.00%)	0	0 (0.00%)	NA
**Systemic AE**	**2**	**2 (0.15%)**	**0**	**0 (0.00%)**	**1**	**1 (0.22%)**	**1**	**1 (0.22%)**	**0**	**0 (0.00%)**	**1.0000[3] 1.0000[4]**
Fever	0	0 (0.00%)	0	0 (0.00%)	0	0 (0.00%)	0	0 (0.00%)	0	0 (0.00%)	NA
Headache †	1	1 (0.11%)	0	0 (0.00%)	1	1 (0.33%)	0	0 (0.00%)	0	0 (0.00%)	1.0000[3] 1.0000[4]
Fatigue †	0	0 (0.00%)	0	0 (0.00%)	0	0 (0.00%)	0	0 (0.00%)	0	0 (0.00%)	NA
Myalgia †	0	0 (0.00%)	0	0 (0.00%)	0	0 (0.00%)	0	0 (0.00%)	0	0 (0.00%)	NA
Lethargy	0	0 (0.00%)	0	0 (0.00%)	0	0 (0.00%)	0	0 (0.00%)	0	0 (0.00%)	NA
Irritability ‡	0	0 (0.00%)	0	0 (0.00%)	0	0 (0.00%)	0	0 (0.00%)	0	0 (0.00%)	NA
Nausea	0	0 (0.00%)	0	0 (0.00%)	0	0 (0.00%)	0	0 (0.00%)	0	0 (0.00%)	NA
Vomiting	1	1 (0.07%)	0	0 (0.00%)	0	0 (0.00%)	1	1 (0.22%)	0	0 (0.00%)	1.0000[3] 1.0000[4]
Arthralgia	0	0 (0.00%)	0	0 (0.00%)	0	0 (0.00%)	0	0 (0.00%)	0	0 (0.00%)	NA
Diarrhea	0	0 (0.00%)	0	0 (0.00%)	0	0 (0.00%)	0	0 (0.00%)	0	0 (0.00%)	NA
Drowsiness	0	0 (0.00%)	0	0 (0.00%)	0	0 (0.00%)	0	0 (0.00%)	0	0 (0.00%)	NA
Loss of appetite	0	0 (0.00%)	0	0 (0.00%)	0	0 (0.00%)	0	0 (0.00%)	0	0 (0.00%)	NA
Chills	0	0 (0.00%)	0	0 (0.00%)	0	0 (0.00%)	0	0 (0.00%)	0	0 (0.00%)	NA
Persistent crying ‡	0	0 (0.00%)	0	0 (0.00%)	0	0 (0.00%)	0	0 (0.00%)	0	0 (0.00%)	NA
Rash *	0	0 (0.00%)	0	0 (0.00%)	0	0 (0.00%)	0	0 (0.00%)	0	0 (0.00%)	NA
Nasopharyngitis *	0	0 (0.00%)	0	0 (0.00%)	0	0 (0.00%)	0	0 (0.00%)	0	0 (0.00%)	NA

[Note] N: number of total participants; n: number of participants who reported events; m: number of events; %: percentages (100*n/N). NA: Not Applicable.
* Only for MR vaccine recipients among the 9-15 months old (Total (N=108) and each Lot 1 (N=24), Lot 2 (N=28), Lot 3 (N=26), and Control (N=30))
† Only for Age Strata 2 and 3 those who are 2 years old and above (Total (N=1200) and each Lot 1 (N=300), Lot 2 (N=300), Lot 3 (N=300), and Control (N=30))
‡ Only for Age Strata 1 those who are less than 2 years (Total (N=600) and each Lot 1 (N=150), Lot 2 (N=150), Lot 3 (N=150), and Control (N=150))
The p-value was derived from [1] Chi-square test or [3] Fisher's exact test compared between Vi-DT (Pooled Lot1+Lot2_Lot3) and Vi-TT.
The p-value was derived from [2] Chi-square test or [4] Fisher's exact test compared among Vi-DT Lot1, Lot2, Lot3, and Vi-TT.

Supplementary Table S17: Distribution of solicited AEs

Within 7 days after vaccination	Vi-DT Group (N=1350)		Lot 1 (N=450)		Lot 2 (N=450)		Lot 3 (N=450)		Vi-TT Group (N=450)		P-value
	m	n (%)	m	n (%)	m	n (%)	m	n (%)	m	n (%)	
**All ages**	**453**	**260 (19.26%)**	**137**	**81 (18.00%)**	**141**	**91 (20.22%)**	**175**	**88 (19.56%)**	**191**	**115 (25.56%)**	**0.0044[1] 0.0318[2]**
**Local AE**	**193**	**170 (12.59%)**	**58**	**50 (11.11%)**	**72**	**65 (14.44%)**	**63**	**55 (12.22%)**	**94**	**86 (19.11%)**	**0.0006[1] 0.0031[2]**
Pain/Tenderness	159	158 (11.70%)	45	45 (10.00%)	60	60 (13.33%)	54	53 (11.78%)	81	81 (18.00%)	0.0007[1] 0.0032[2]
Erythema/Redness	11	11 (0.81%)	6	6 (1.33%)	4	4 (0.89%)	1	1 (0.22%)	6	6 (1.33%)	0.3963[3] 0.2187[4]
Swelling/Induration	7	7 (0.52%)	2	2 (0.44%)	3	3 (0.67%)	2	2 (0.44%)	3	3 (0.67%)	0.7179[3] 1.0000[4]
Pruritus	9	9 (0.67%)	4	4 (0.89%)	3	3 (0.67%)	2	2 (0.44%)	2	2 (0.44%)	0.7411[3] 0.9111[4]
Pain/Tenderness (MR) *	4	4 (5.13%)	0	0 (0.00%)	1	1 (3.57%)	3	3 (11.54%)	2	2 (6.67%)	0.6690[3] 0.4002[4]
Erythema/Redness (MR) *	3	3 (3.85%)	1	1 (4.17%)	1	1 (3.57%)	1	1 (3.85%)	0	0 (0.00%)	0.5587[3] 0.7026[4]
Swelling/Induration (MR) *	0	0 (0.00%)	0	0 (0.00%)	0	0 (0.00%)	0	0 (0.00%)	0	0 (0.00%)	NA
Pruritus (MR)	0	0 (0.00%)	0	0 (0.00%)	0	0 (0.00%)	0	0 (0.00%)	0	0 (0.00%)	NA
**Systemic AE**	**260**	**140 (10.37%)**	**79**	**44 (9.78%)**	**69**	**42 (9.33%)**	**112**	**54 (12.00%)**	**97**	**49 (10.89%)**	**0.7560[1] 0.5618[2]**
Fever	49	45 (3.33%)	10	9 (2.00%)	18	17 (3.78%)	21	19 (4.22%)	21	18 (4.00%)	0.5051[1] 0.2479[2]
Headache †	37	33 (3.67%)	9	9 (3.00%)	13	10 (3.33%)	15	14 (4.67%)	13	11 (3.67%)	1.0000[1] 0.7241[2]
Fatigue †	10	9 (1.00%)	4	4 (1.33%)	1	1 (0.33%)	5	4 (1.33%)	5	4 (1.33%)	0.7471[3] 0.5372[4]
Myalgia †	6	5 (0.56%)	2	2 (0.67%)	1	1 (0.33%)	3	2 (0.67%)	1	1 (0.33%)	1.0000[3] 1.0000[4]
Lethargy	22	21 (1.56%)	6	6 (1.33%)	4	4 (0.89%)	12	11 (2.44%)	4	4 (0.89%)	0.2953[1] 0.1502[2]
Irritability ‡	6	6 (1.33%)	3	3 (2.00%)	0	0 (0.00%)	3	3 (2.00%)	8	8 (5.33%)	0.0095[3] 0.0174[4]
Nausea	14	14 (1.04%)	4	4 (0.89%)	4	4 (0.89%)	6	6 (1.33%)	6	5 (1.11%)	1.0000[3] 0.9567[4]
Vomiting	33	31 (2.30%)	13	12 (2.67%)	8	7 (1.56%)	12	12 (2.67%)	11	10 (2.22%)	0.9273[1] 0.6431[2]
Arthralgia	9	9 (0.67%)	4	4 (0.89%)	3	3 (0.67%)	2	2 (0.44%)	1	1 (0.22%)	0.4669[3] 0.7179[4]
Diarrhea	37	35 (2.59%)	14	14 (3.11%)	6	6 (1.33%)	17	15 (3.33%)	10	10 (2.22%)	0.6630[1] 0.2013[2]
Drowsiness	13	11 (0.81%)	6	5 (1.11%)	2	2 (0.44%)	5	4 (0.89%)	4	4 (0.89%)	1.0000[3] 0.7983[4]
Loss of appetite	10	10 (0.74%)	2	2 (0.44%)	4	4 (0.89%)	4	4 (0.89%)	4	4 (0.89%)	0.7590[3] 0.8545[4]
Chills	4	4 (0.30%)	1	1 (0.22%)	2	2 (0.44%)	1	1 (0.22%)	3	3 (0.67%)	0.3763[3] 0.8454[4]
Persistent crying ‡	6	6 (1.33%)	0	0 (0.00%)	1	1 (0.67%)	5	5 (3.33%)	2	2 (1.33%)	1.0000[3] 0.0918[4]
Rash*	0	0 (0.00%)	0	0 (0.00%)	0	0 (0.00%)	0	0 (0.00%)	0	0 (0.00%)	NA
Nasopharyngitis*	4	4 (5.13%)	1	1 (4.17%)	2	2 (7.14%)	1	1 (3.85%)	4	4 (13.33%)	0.2140[3] 0.5888[4]

[Note] N: number of total participants; n: number of participants who reported events; m: number of events; %: percentages (100*n/N). NA: Not Applicable.

* Only for MR vaccine recipients among the 9-15 months old (Total (N=108) and each Lot 1 (N=29), Lot 2 (N=28), Lot 3 (N=27), and Control (N=24)).

† Only for Age Strata 2 and 3 those who are 2 years old and above (Total (N=900) and each Lot 1 (N=300), Lot 2 (N=300), Lot 3 (N=300), and Control (N=300))

‡ Only for Age Strata 1 those who are less than 2 years (Total (N=600) and each Lot 1 (N=150), Lot 2 (N=150), Lot 3 (N=150), and Control (N=150))
The p-value was derived from [1] Chi-square test or [3] Fisher’s exact test compared between Vi-DT (Pooled Lot1+Lot2_Lot3) and Vi-TT.
The p-value was derived from [2] Chi-square test or [4] Fisher’s exact test compared among Vi-DT Lot1, Lot2, Lot3, and Vi-TT.

Supplementary Table S18: Distribution of unsolicited AEs within 4 weeks after vaccination

SOC\PT	Vi-DT Group (N=1350)		Lot 1 (N=450)		Lot 2 (N=450)		Lot 3 (N=450)		Vi-TT Group (N=450)		P-value
**Within 4 weeks after the dose**	**m**	**n (%)**	**m**	**n (%)**	**m**	**n (%)**	**m**	**n (%)**	**m**	**n (%)**	
**All ages**	**361**	**208 (15.41%)**	**110**	**63 (14.00%)**	**135**	**74 (16.44%)**	**116**	**71 (15.78%)**	**143**	**76 (16.89%)**	**0.4553[1] 0.6506[2]**
Ear and labyrinth disorders	3	3 (0.22%)	2	2 (0.44%)	0	0 (0.00%)	1	1 (0.22%)	1	1 (0.22%)	1.0000[3] 0.9059[4]
Ear pain	2	2 (0.15%)	1	1 (0.22%)	0	0 (0.00%)	1	1 (0.22%)	1	1 (0.22%)	1.0000[3] 1.0000[4]
Ear pruritus	1	1 (0.07%)	1	1 (0.22%)	0	0 (0.00%)	0	0 (0.00%)	0	0 (0.00%)	1.0000[3] 1.0000[4]
Eye disorders	2	2 (0.15%)	2	2 (0.44%)	0	0 (0.00%)	0	0 (0.00%)	0	0 (0.00%)	1.0000[3] 0.2496[4]
Eye discharge	1	1 (0.07%)	1	1 (0.22%)	0	0 (0.00%)	0	0 (0.00%)	0	0 (0.00%)	1.0000[3] 1.0000[4]
Eye pain	1	1 (0.07%)	1	1 (0.22%)	0	0 (0.00%)	0	0 (0.00%)	0	0 (0.00%)	1.0000[3] 1.0000[4]
Gastrointestinal disorders	54	44 (3.26%)	16	13 (2.89%)	23	18 (4.00%)	15	13 (2.89%)	19	11 (2.44%)	0.3844[1] 0.5710[2]
Abdominal distension	1	1 (0.07%)	0	0 (0.00%)	0	0 (0.00%)	1	1 (0.22%)	0	0 (0.00%)	1.0000[3] 1.0000[4]
Abdominal pain	7	7 (0.52%)	2	2 (0.44%)	5	5 (1.11%)	0	0 (0.00%)	0	0 (0.00%)	0.1259[1] 0.0204[4]
Abdominal pain upper	1	1 (0.07%)	0	0 (0.00%)	0	0 (0.00%)	1	1 (0.22%)	0	0 (0.00%)	1.0000[3] 1.0000[4]
Constipation	2	2 (0.15%)	1	1 (0.22%)	1	1 (0.22%)	0	0 (0.00%)	0	0 (0.00%)	1.0000[3] 1.0000[4]
Diarrhoea	21	21 (1.56%)	5	5 (1.11%)	8	8 (1.78%)	8	8 (1.78%)	8	8 (1.78%)	0.7457[1] 0.8142[2]
Diarrhoea haemorrhagic	1	1 (0.07%)	1	1 (0.22%)	0	0 (0.00%)	0	0 (0.00%)	0	0 (0.00%)	1.0000[3] 1.0000[4]
Gastritis	1	1 (0.07%)	0	0 (0.00%)	0	0 (0.00%)	1	1 (0.22%)	0	0 (0.00%)	1.0000[3] 1.0000[4]
Gingival swelling	0	0 (0.00%)	0	0 (0.00%)	0	0 (0.00%)	0	0 (0.00%)	1	1 (0.22%)	0.2500[3] 1.0000[4]
Mouth ulceration	1	1 (0.07%)	1	1 (0.22%)	0	0 (0.00%)	0	0 (0.00%)	1	1 (0.22%)	0.4376[3] 1.0000[4]
Nausea	1	1 (0.07%)	0	0 (0.00%)	1	1 (0.22%)	0	0 (0.00%)	0	0 (0.00%)	1.0000[3] 1.0000[4]
Vomiting	17	17 (1.26%)	6	6 (1.33%)	8	8 (1.78%)	3	3 (0.67%)	9	8 (1.78%)	0.4157[1] 0.4372[2]
Dental discomfort	1	1 (0.07%)	0	0 (0.00%)	0	0 (0.00%)	1	1 (0.22%)	0	0 (0.00%)	1.0000[3] 1.0000[4]
General disorders and administration site conditions	64	62 (4.59%)	21	19 (4.22%)	24	24 (5.33%)	19	19 (4.22%)	25	24 (5.33%)	0.5235[1] 0.7479[2]
Asthenia	1	1 (0.07%)	1	1 (0.22%)	0	0 (0.00%)	0	0 (0.00%)	0	0 (0.00%)	1.0000[3] 1.0000[4]
Chest pain	1	1 (0.07%)	0	0 (0.00%)	0	0 (0.00%)	1	1 (0.22%)	0	0 (0.00%)	1.0000[3] 1.0000[4]
Pyrexia	60	60 (4.44%)	19	19 (4.22%)	24	24 (5.33%)	17	17 (3.78%)	25	24 (5.33%)	0.4388[1] 0.5938[2]
Swelling face	1	1 (0.07%)	1	1 (0.22%)	0	0 (0.00%)	0	0 (0.00%)	0	0 (0.00%)	1.0000[3] 1.0000[4]
Peripheral swelling	1	1 (0.07%)	0	0 (0.00%)	0	0 (0.00%)	1	1 (0.22%)	0	0 (0.00%)	1.0000[3] 1.0000[4]
Hepatobiliary disorders	0	0 (0.00%)	0	0 (0.00%)	0	0 (0.00%)	0	0 (0.00%)	1	1 (0.22%)	0.2500[3] 1.0000[4]
Jaundice	0	0 (0.00%)	0	0 (0.00%)	0	0 (0.00%)	0	0 (0.00%)	1	1 (0.22%)	0.2500[3] 1.0000[4]
Infections and infestations	76	73 (5.41%)	20	19 (4.22%)	26	24 (5.33%)	30	30 (6.67%)	34	32 (7.11%)	0.1817[1] 0.2369[2]
Bronchiolitis	0	0 (0.00%)	0	0 (0.00%)	0	0 (0.00%)	0	0 (0.00%)	1	1 (0.22%)	0.2500[3] 1.0000[4]

Conjunctivitis	3	3 (0.22%)	0	0 (0.00%)	2	2 (0.44%)	1	1 (0.22%)	0	0 (0.00%)	0.5777[3] 0.6244[4]
Gastroenteritis	2	2 (0.15%)	0	0 (0.00%)	1	1 (0.22%)	1	1 (0.22%)	1	1 (0.22%)	1.0000[3] 1.0000[4]
Hand-foot-and-mouth disease	1	1 (0.07%)	0	0 (0.00%)	0	0 (0.00%)	1	1 (0.22%)	0	0 (0.00%)	1.0000[3] 1.0000[4]
Lower respiratory tract infection	1	1 (0.07%)	0	0 (0.00%)	0	0 (0.00%)	1	1 (0.22%)	1	1 (0.22%)	0.4376[3] 1.0000[4]
Mumps	1	1 (0.07%)	1	1 (0.22%)	0	0 (0.00%)	0	0 (0.00%)	0	0 (0.00%)	1.0000[3] 1.0000[4]
Nasopharyngitis	45	45 (3.33%)	12	12 (2.67%)	17	17 (3.78%)	16	16 (3.56%)	21	20 (4.44%)	0.2739[1] 0.5538[2]
Otitis media chronic	1	1 (0.07%)	1	1 (0.22%)	0	0 (0.00%)	0	0 (0.00%)	0	0 (0.00%)	1.0000[3] 1.0000[4]
Pharyngitis	0	0 (0.00%)	0	0 (0.00%)	0	0 (0.00%)	0	0 (0.00%)	2	2 (0.44%)	0.0624[3] 0.2496[4]
Pneumonia	1	1 (0.07%)	0	0 (0.00%)	1	1 (0.22%)	0	0 (0.00%)	0	0 (0.00%)	1.0000[3] 1.0000[4]
Rhinitis	1	1 (0.07%)	0	0 (0.00%)	0	0 (0.00%)	1	1 (0.22%)	1	1 (0.22%)	0.4376[3] 1.0000[4]
Subcutaneous abscess	0	0 (0.00%)	0	0 (0.00%)	0	0 (0.00%)	0	0 (0.00%)	1	1 (0.22%)	0.2500[3] 1.0000[4]
Tonsillitis	1	1 (0.07%)	1	1 (0.22%)	0	0 (0.00%)	0	0 (0.00%)	0	0 (0.00%)	1.0000[3] 1.0000[4]
Upper respiratory tract infection	13	13 (0.96%)	4	4 (0.89%)	3	3 (0.67%)	6	6 (1.33%)	4	4 (0.89%)	1.0000[3] 0.8282[4]
Urinary tract infection	0	0 (0.00%)	0	0 (0.00%)	0	0 (0.00%)	0	0 (0.00%)	1	1 (0.22%)	0.2500[3] 1.0000[4]
Viral rash	1	1 (0.07%)	0	0 (0.00%)	1	1 (0.22%)	0	0 (0.00%)	0	0 (0.00%)	1.0000[3] 1.0000[4]
Tinea infection	1	1 (0.07%)	0	0 (0.00%)	0	0 (0.00%)	1	1 (0.22%)	0	0 (0.00%)	1.0000[3] 1.0000[4]
Respiratory tract infection	2	2 (0.15%)	0	0 (0.00%)	1	1 (0.22%)	1	1 (0.22%)	0	0 (0.00%)	1.0000[3] 1.0000[4]
Acarodermatitis	2	2 (0.15%)	1	1 (0.22%)	0	0 (0.00%)	1	1 (0.22%)	1	1 (0.22%)	1.0000[3] 1.0000[4]
Injury, poisoning and procedural complications	3	3 (0.22%)	1	1 (0.22%)	2	2 (0.44%)	0	0 (0.00%)	2	2 (0.44%)	0.6041[3] 0.7648[4]
Thermal burn	1	1 (0.07%)	1	1 (0.22%)	0	0 (0.00%)	0	0 (0.00%)	1	1 (0.22%)	0.4376[3] 1.0000[4]
Tongue injury	1	1 (0.07%)	0	0 (0.00%)	1	1 (0.22%)	0	0 (0.00%)	0	0 (0.00%)	1.0000[3] 1.0000[4]
Limb injury	1	1 (0.07%)	0	0 (0.00%)	1	1 (0.22%)	0	0 (0.00%)	0	0 (0.00%)	1.0000[3] 1.0000[4]
Procedural pain	0	0 (0.00%)	0	0 (0.00%)	0	0 (0.00%)	0	0 (0.00%)	1	1 (0.22%)	0.2500[3] 1.0000[4]
Metabolism and nutrition disorders	1	1 (0.07%)	1	1 (0.22%)	0	0 (0.00%)	0	0 (0.00%)	0	0 (0.00%)	1.0000[3] 1.0000[4]
Vitamin D deficiency	1	1 (0.07%)	1	1 (0.22%)	0	0 (0.00%)	0	0 (0.00%)	0	0 (0.00%)	1.0000[3] 1.0000[4]
Musculoskeletal and connective tissue disorders	3	3 (0.22%)	2	2 (0.44%)	0	0 (0.00%)	1	1 (0.22%)	1	1 (0.22%)	1.0000[3] 0.9059[4]
Back pain	2	2 (0.15%)	1	1 (0.22%)	0	0 (0.00%)	1	1 (0.22%)	0	0 (0.00%)	1.0000[3] 1.0000[4]
Pain in extremity	1	1 (0.07%)	1	1 (0.22%)	0	0 (0.00%)	0	0 (0.00%)	1	1 (0.22%)	0.4376[3] 1.0000[4]
Nervous system disorders	9	8 (0.59%)	3	2 (0.44%)	4	4 (0.89%)	2	2 (0.44%)	2	2 (0.44%)	1.0000[3] 0.8548[4]
Dizziness	2	1 (0.07%)	2	1 (0.22%)	0	0 (0.00%)	0	0 (0.00%)	0	0 (0.00%)	1.0000[3] 1.0000[4]
Headache	5	5 (0.37%)	1	1 (0.22%)	2	2 (0.44%)	2	2 (0.44%)	2	2 (0.44%)	1.0000[3] 1.0000[4]
Lethargy	1	1 (0.07%)	0	0 (0.00%)	1	1 (0.22%)	0	0 (0.00%)	0	0 (0.00%)	1.0000[3] 1.0000[4]
Seizure	1	1 (0.07%)	0	0 (0.00%)	1	1 (0.22%)	0	0 (0.00%)	0	0 (0.00%)	1.0000[3] 1.0000[4]
Psychiatric disorders	0	0 (0.00%)	0	0 (0.00%)	0	0 (0.00%)	0	0 (0.00%)	1	1 (0.22%)	0.2500[3] 1.0000[4]
Breath holding	0	0 (0.00%)	0	0 (0.00%)	0	0 (0.00%)	0	0 (0.00%)	1	1 (0.22%)	0.2500[3] 1.0000[4]

Renal and urinary disorders	1	1 (0.07%)	0	0 (0.00%)	1	1 (0.22%)	0	0 (0.00%)	0	0 (0.00%)	1.0000[3] 1.0000[4]
Dysuria	1	1 (0.07%)	0	0 (0.00%)	1	1 (0.22%)	0	0 (0.00%)	0	0 (0.00%)	1.0000[3] 1.0000[4]
Reproductive system and breast disorders	2	2 (0.15%)	1	1 (0.22%)	1	1 (0.22%)	0	0 (0.00%)	0	0 (0.00%)	1.0000[3] 1.0000[4]
Dysmenorrhoea	2	2 (0.15%)	1	1 (0.22%)	1	1 (0.22%)	0	0 (0.00%)	0	0 (0.00%)	1.0000[3] 1.0000[4]
Respiratory, thoracic and mediastinal disorders	128	112 (8.30%)	38	31 (6.89%)	48	42 (9.33%)	42	39 (8.67%)	53	47 (10.44%)	0.1643[1] 0.2935[2]
Cough	109	106 (7.85%)	32	31 (6.89%)	41	39 (8.67%)	36	36 (8.00%)	48	45 (10.00%)	0.1546[1] 0.3961[2]
Nasal congestion	1	1 (0.07%)	1	1 (0.22%)	0	0 (0.00%)	0	0 (0.00%)	0	0 (0.00%)	1.0000[3] 1.0000[4]
Respiratory disorder	1	1 (0.07%)	0	0 (0.00%)	0	0 (0.00%)	1	1 (0.22%)	0	0 (0.00%)	1.0000[3] 1.0000[4]
Rhinorrhoea	12	12 (0.89%)	4	4 (0.89%)	5	5 (1.11%)	3	3 (0.67%)	3	3 (0.67%)	0.7738[3] 0.9406[4]
Throat irritation	0	0 (0.00%)	0	0 (0.00%)	0	0 (0.00%)	0	0 (0.00%)	1	1 (0.22%)	0.2500[3] 1.0000[4]
Allergic cough	1	1 (0.07%)	0	0 (0.00%)	0	0 (0.00%)	1	1 (0.22%)	0	0 (0.00%)	1.0000[3] 1.0000[4]
Oropharyngeal pain	3	3 (0.22%)	0	0 (0.00%)	2	2 (0.44%)	1	1 (0.22%)	1	1 (0.22%)	1.0000[3] 0.9059[4]
Sinonasal obstruction	1	1 (0.07%)	1	1 (0.22%)	0	0 (0.00%)	0	0 (0.00%)	0	0 (0.00%)	1.0000[3] 1.0000[4]
Skin and subcutaneous tissue disorders	15	15 (1.11%)	3	3 (0.67%)	6	6 (1.33%)	6	6 (1.33%)	4	4 (0.89%)	0.7966[3] 0.7367[4]
Dermatitis allergic	1	1 (0.07%)	0	0 (0.00%)	1	1 (0.22%)	0	0 (0.00%)	0	0 (0.00%)	1.0000[3] 1.0000[4]
Dermatitis contact	1	1 (0.07%)	1	1 (0.22%)	0	0 (0.00%)	0	0 (0.00%)	1	1 (0.22%)	0.4376[3] 1.0000[4]
Eczema	5	5 (0.37%)	0	0 (0.00%)	2	2 (0.44%)	3	3 (0.67%)	0	0 (0.00%)	0.3401[3] 0.1788[4]
Panniculitis	1	1 (0.07%)	0	0 (0.00%)	0	0 (0.00%)	1	1 (0.22%)	0	0 (0.00%)	1.0000[3] 1.0000[4]
Pruritus	1	1 (0.07%)	0	0 (0.00%)	0	0 (0.00%)	1	1 (0.22%)	0	0 (0.00%)	1.0000[3] 1.0000[4]
Rash	4	4 (0.30%)	1	1 (0.22%)	2	2 (0.44%)	1	1 (0.22%)	3	3 (0.67%)	0.3763[3] 0.8454[4]
Skin lesion	1	1 (0.07%)	0	0 (0.00%)	1	1 (0.22%)	0	0 (0.00%)	0	0 (0.00%)	1.0000[3] 1.0000[4]
Urticaria papular	1	1 (0.07%)	1	1 (0.22%)	0	0 (0.00%)	0	0 (0.00%)	0	0 (0.00%)	1.0000[3] 1.0000[4]

[Note] N: number of total participants; n: number of participants who reported events; m: number of events; %: percentages (100*n/N). NA: Not Applicable.
The p-value was derived from [1] Chi-square test or [3] Fisher’s exact test compared between Vi-DT (Pooled Lot1+Lot2_Lot3) and Vi-TT.
The p-value was derived from [2] Chi-square test or [4] Fisher’s exact test compared among Vi-DT Lot1, Lot2, Lot3, and Vi-TT.

Supplementary Table S15: Summary of Major Protocol Deviations

	Vi-DT Group				Vi-TT Group	Total
		Lot 1	Lot 2	Lot 3		
Reasons (%)	N=1,350	N=450	N=450	N=450	N=450	N=1,800
2nd dose MR vaccination (over 15-month child)	1 (0.07)	0 (0.00)	0 (0.00)	1 (0.22)	1 (0.22)	2 (0.11)
2nd dose MR vaccination (post vaccination)	1 (0.07)	0 (0.00)	1 (0.22)	0 (0.00)	0 (0.00)	1 (0.06)
Missing Visits (Visit 4)	4 (0.30)	3 (0.67)	0 (0.00)	1 (0.22)	0 (0.00)	4 (0.22)
Rejection of blood collection on Visit 4	1 (0.07)	1 (0.22)	0 (0.00)	0 (0.00)	0 (0.00)	1 (0.06)
Visit outside window (Visit 4)	13 (0.96)	2 (0.44)	4 (0.89)	7 (1.56)	3 (0.67)	16 (0.89)
Visit outside window (Onsite on V6)	69 (5.11)	20 (4.44)	26 (5.78)	23 (5.11)	19 (4.22)	88 (4.89)
Visit outside window (Telephonic on V6)	92 (6.81)	35 (7.78)	28 (6.22)	29 (6.44)	36 (8.00)	128 (7.11)
Total	181 (13.41)	61 (13.56)	59 (13.11)	61 (13.56)	59 (13.11)	240 (13.33)
