# Supplementary material for: Safety and immunogenicity of the Vi-DT typhoid conjugate vaccine in healthy volunteers in Nepal: an observer-blind, active-controlled, randomised, non-inferiority, phase 3 trial
Source: Lancet Infect Dis. 2022 Apr;22(4):529–40. doi: 10.1016/S1473-3099(21)00455-2 (PMC8942857; doi:10.1016/S1473-3099(21)00455-2)
Supplement: Supplementary appendix 3 [file mmc3.pdf]

THE LANCET
Infectious Diseases

Supplementary appendix 3

This appendix formed part of the original submission and has been peer reviewed. We post it as supplied by the authors.

Supplement to: Rai GK, Saluja T, Chaudhary S, et al. Safety and immunogenicity of the Vi-DT typhoid conjugate vaccine in healthy volunteers in Nepal: an observer-blind, active-controlled, randomised, non-inferiority, phase 3 trial. *Lancet Infect Dis* 2021; published online Dec 20. https://doi.org/10.1016/S1473-3099(21)00455-2.

A Phase III Multicenter, Observer-Blinded, Randomized, Active Controlled, Immune Non-inferiority and Safety Study of Diphtheria Toxoid Conjugated Vi-Polysaccharide Typhoid Vaccine compared to Typbar TCV® in healthy 6 months-45 years aged Nepalese participants.

**Protocol Number:**	**IVI T003**
**Principal Investigator:**	Dr.Ganesh Rai<br><br>Kanti Children's Hospital,<br>KMPC-03, Maharajgunj, Kathmandu,Nepal<br><br>Phone: +977 985 1044163<br>drganeshrai@gmail.com
**Sponsor:**	International Vaccine Institute<br>SNU Research Park, 1 Gwanak-ro,<br>Gwanak-gu, Seoul, 151-742<br>Republic of Korea
**Co-sponsor**	SK bioscience<br>310 Pangyo-ro, Bundang-gu,<br>Seongnam-si, Gyeonggi-do, 13494,<br>Seoul, Republic of Korea
**Funding Agency:**	Bill and Melinda Gates Foundation<br>Seattle, WA, USA
**Version Number, Date:**	Version 9.0 17NOV2020

FOR OFFICIAL USE ONLY
Information and data included in this document contain privileged and/or proprietary information, which is the property of the International Vaccine Institute, SK bioscience, and participating clinical trial sites in Nepal, and may not be reproduced, published or disclosed to others without written authorization. These restrictions on disclosure will apply equally to all future information, which is indicated as privileged or proprietary.

KEY ROLES

1. Sites Principal Investigators

- Dr.Ganesh Rai

  Study Principal Investigator
  Kanti Children's Hospital,
  KMPC-03, Maharajgunj, Kathmandu,Nepal
  Phone: +977 985 1044163
  drganeshrai@gmail.com

- Dr. Shipra Chaudhary

  Assosiate Professor
  Department of Pediatrics and Adolescent Medicine
  B P Koirala Institute of Health Sciences (BPKIHS)
  Dharan, Nepal
  Phone: +977 984 2054313
  nowshipra@gmail.com

- Dr. Dipesh Tamrakar

  Assistant Professor
  Department of community medicine,
  School of Medical Sciences, Kathmandu University, Dhulikhel
  Phone: +977 980 1002404
  bob.dipesh@gmail.com

- Dr. Piush Kanodia

  Assosiate Professor
  Department.of Pediatrics
  Nepalgunj Medical College, Nepalgunj
  Phone: +977 985 8024990
  piushkanodia@yahoo.com

2. Contract Research Organization
    - Zifo RnD Solutions
      21A Anna Salai, Little Mount,
      Saidapet, Chennai, Tamil Nadu, India

    - Croissance Clinical Research Pvt. Ltd.,
      402, Plot No. 91, 92, 93, Vasu Heights, Lumbini Avenue, OPP.
      Care Hospital, Gachibowli, Hyderabad, 500 032, Telangana
      India

3. IVI Personnel

    i. Study Medical Monitor
    Dr. Tarun Saluja
    Phone: +82-2-881-1236
    tarun.saluja@ivi.int

    ii. Investigator & Head of Clinical Development & Regulatory
    Dr. Anh Wartel
    Phone: +82-2-881-1274
    anh.wartel@ivi.int

    iii. Head of Biostatistics and Data Management
    Dr. Deok Ryun Kim
    Phone: +82-2-881-1140,
    drkim@ivi.int

    iv. Clinical Operations
    BoMi Kim
    Phone: +82-2-881-1279
    bomi.kim@ivi.int

    v. Endpoint Laboratory
    Dr. Jae Seung Yang
    Phone: +82-2-881-1198
    jsyang@ivi.int

    vi. Head of Quality Management
    Tobin Guarnacci
    Phone: +82 2 881 1131
    Tobin.guarnacci@ivi.int

TABLE OF CONTENTS

Key Roles 2
List of Abbreviations 7
1 SYNOPSIS 9
2 INTRODUCTION 19
2.1 Background 19
3 Vi-DT CONJUGATE VACCINE 24
3.1 Preclinical Data 24
3.2 Clinical Data 25
3.3 Study Rationale 26
3.4 Potential Risks and Benefits 28
3.4.1 Known Potential Risks 28
3.4.2 Known Potential Benefits 29
4 OBJECTIVES 29
5 STUDY DESIGN 30
5.1 Study Endpoints 30
5.2 Overall Design 32
5.3 Measures to Minimize Bias 36
5.3.1 Randomization/Masking Procedures 36
5.3.2 Unblinding of Participants 37
6 STUDY AGENT 37
6.1 Study Agent(s) and Control Description 37
6.1.1 Acquisition 37
6.1.2 Formulation, Appearance, Packaging, and Labeling 37
6.1.3 Preparation 39
6.1.4 Dosing and Route of Administration 39
6.1.5 Dose Adjustments/Modifications/Delays 40
6.1.6 Tracking of Dose 40
6.2 Study Agent Accountability Procedures 40
6.3 Standard of Care 40
6.4 Concomitant Medications, Treatments, and Procedures 41
7 STUDY POPULATION 41
7.1 Strategies for Recruitment and Retention 41
7.2 Consent and Assent Procedures and Documentation 41
7.3 Compensation for Participation 42
7.4 Participant Inclusion Criteria 43
7.5 Participant Exclusion Criteria 43
7.6 Study Procedures 44
7.6.1 Screening 44
7.6.2 Enrollment 45
7.6.3 Follow-up Procedures and Visits 46
7.7 Participant Withdrawal or Termination 48
7.7.1 Reasons for Withdrawal or Termination 48
7.7.2 Handling of Participant Discontinuation or Termination 48
7.8 Lost to Follow-Up 49

7.9 Protocol Deviations 50
7.10 Protocol Amendments 50
7.11 Premature Termination or Suspension of Study 51
7.12 End of Study 51
8 LABORATORY PROCEDURES/EVALUATIONS 51
8.1 Specimen Processing, Handling, and Storage 51
8.2 Specimen Shipment 52
8.3 Assessment of Immunogenicity 52
9 ASSESSMENT OF SAFETY 52
9.1 Safety Assessment 52
9.1.1 Definition of Adverse Events (AE) 53
9.1.2 Definition of Serious Adverse Events (SAE) 53
9.1.3 Suspected Unexpected Serious Adverse Reactions (SUSAR) 54
9.2 Classification of an Adverse Event 54
9.2.1 Severity of Event 54
9.2.2 Relationship to Investigational Product 58
9.2.3 Expectedness 59
9.3 Time Period and Frequency for Event Assessment and Follow-Up 59
9.4 Reporting Procedures 60
9.4.1 Adverse Event Recording and Reporting 60
9.4.2 Serious Adverse Event Reporting 61
9.4.3 Safety Oversight 62
10 STUDY MONITORING 62
11 STATISTICAL CONSIDERATIONS 63
11.1 Sample Size 63
11.2 Statistical Analysis Plan 64
11.3 Statistical Hypotheses 65
11.4 Analysis Datasets 66
11.5 Description of Statistical Methods 67
11.5.1 General Approach 67
11.5.2 Baseline Descriptive Statistics 67
11.5.3 Safety Analysis 68
11.5.4 Analysis of the Primary Immunogenicity Endpoint(s) 68
11.5.5 Analysis of the Secondary Immunogenicity Endpoint(s) 69
11.5.6 Adherence and Retention Analyses 70
11.5.7 Planned Interim Analysis 71
11.5.8 Additional Sub-Group Analysis 71
11.5.9 Multiple Comparison/Multiplicity 71
11.5.10 Exploratory Analyses 71
12 SOURCE DOCUMENTS AND ACCESS TO SOURCE DOCUMENTS 71
13 DATA HANDLING AND RECORD KEEPING 72
13.1 Data Collection and Management Responsibilities 72
13.2 Study Records Retention 73
13.3 Publication and Data Sharing Policy 73
14 QUALITY ASSURANCE AND QUALITY CONTROL 74
15 ETHICS/PROTECTION OF HUMAN PARTICIPANTS 74

15.1 Regulatory and Ethical Compliance 75
15.2 Participant and Data Confidentiality 75
15.3. Research Use of Stored Human Samples 76
15.4 Future Use of Stored Specimens 76
16 REFERENCES 77
17 APPENDICES 79

a. Statement of Compliance
b. Vi-DT Phase II result summary

LIST OF ABBREVIATIONS

AE	Adverse Event
BMGF	Bill and Melinda Gates Foundation
BSA	Bovine Serum Albumin
°C	Degree Celsius
CI	Confidence Interval
CIOMS	Council for International Organizations of Medical Sciences
CSR	Clinical Study Report
DDA	Department of drug Administration
DOB	Date of Birth
DSMB	Data Safety Monitoring Board
DT	Diphtheria Toxoid
EC	Ethics Committee
eCRF	Electronic Case Report Form
EDTA	Ethylenediaminetetraacetic Acid
ELISA	Enzyme Linked Immunosorbent Assay
EPI	Expanded Program on Immunization
FAS	Full Analysis Set
FDA	Food and Drug Administration
GCP	Good Clinical Practices
GCLP	Good Clinical Laboratory Practices
GMP	Good Manufacturing Practice
GMR	Geometric Mean Ratio
GMT	Geometric Mean Titer
HIV	Human Immunodeficiency Virus
IB	Investigator's Brochure
ICF	Informed Consent Form
IAF	Informed Assent Form
ICH	International Council for Harmonization
IgG	Immunoglobulin G
IL	Interleukin
IRB	Institutional Review Board
IVI	International Vaccine Institute
LAR	Legally Acceptable Representative
M-ITT	Modified Intention-To-Treat
MOP	Manual of Procedures
MR	Measles-Rubella
MMR	Measles-Mumps-Rubella
NHRC	Nepal Health Research Council
µg	Microgram
N	Number
nm	Nanometer

PBS	Phosphate Buffered Saline
PI	Principal Investigator
PP	Per Protocol
QA	Quality Assurance
QC	Quality Control
SAE	Serious Adverse Event
SAP	Statistical Analysis Plan
SBA	Serum Bactericidal Antibody
SI	Study investigator
SOE	Schedule of Events
SUSAR	Suspected Unexpected Serious Adverse Reaction
Vi	*Salmonella* Typhi Capsular Polysaccharide
Vi-DT	Diphtheria Toxoid Conjugated Vi-Polysaccharide Vaccine
Vi-PS	*Salmonella* Typhi Capsular Polysaccharide Vaccine
Vi-rEPA	*Pseudomonas aeruginosa* exotoxin A Conjugated Vi-Polysaccharide Vaccine
Vi-TT	Tetanus Toxoid Conjugated Vi-Polysaccharide Vaccine
WHO	World Health Organization

1 SYNOPSIS

**Name of the Sponsor:** International Vaccine Institute (IVI)	
**Name of Investigational Product:** Vi-DT	
**Name of Active Ingredients:** Diphtheria Toxoid Conjugated Vi-Polysaccharide Typhoid Vaccine	
**Title of Study:**<br><br>A Phase III Multicenter, Observer-Blinded, Randomized, Active Controlled, Immune Non-inferiority and Safety Study of Diphtheria Toxoid Conjugated Vi-Polysaccharide Typhoid Vaccine compared to Typbar TCV® in healthy 6 months-45 years aged Nepalese participants	
**Protocol Number:** IVI T003	
**Study Sites:** Kanti Children's Hospital<br>Nepalgunj Medical College<br>Dhulikhel Hospital<br>B.P.Koirala Institute of Health Sciences	
**Principal Investigator:** Dr. Ganesh Rai<br><br>**Site Investigators:** Dr. Shipra Chaudhary<br>Dr. Dipesh Tamrakar<br>Dr. Piush Kanodia<br><br>**Data Management and Biostatistics :** Dr. Deok Ryun Kim<br><br>**Research Laboratory :** Dr. Jae Seung Yang<br><br>**Study Medical Monitor**: Dr. Tarun Saluja	
**Study Period (years/months)**<br><br>Estimated date first participant enrolled: Nov 2019<br>Estimated date last participant enrolled: APR 2020<br>Estimated duration of the trial: 18 months<br>**Additonal group (years/months)**	**Phase of development:** 3

Estimated date first participant enrolled: SEP 2020<br>Estimated date last participant enrolled: NOV 2020<br>Estimated duration of the trial: 6 months	

Study Hypothesis

To establish immune non-inferiority of a single dose of Vi-DT compared to locally licensed typhoid conjugate vaccine (Typbar TCV®, Bharat Biotech) in participants of 6 months to 45 years of age and to assess immune non-inferiority for all age groups combined at 4 weeks and persistence of antibodies at 24 weeks post vaccination. The sample size calculation is based on the non-inferiority hypothesis of the immunogenicity endpoint and on lot-to-lot consistency. The safety of Vi-DT in comparison to Typbar TCV® will also be assessed by analyzing safety data descriptively for 4 and 24 weeks.

Objectives

Primary

- Demonstrate non-inferiority of Vi-DT compared to Typbar TCV® as measured by seroconversion rates of anti-Vi IgG ELISA antibody titers, 4 weeks after single dose (pooled immunogenicity of three lots of Vi-DT)
- Demonstrate the equivalence of immunogenicity as measured by anti-Vi IgG GMT of three lots of Vi-DT vaccine 4 weeks after single dose.

Secondary

- Demonstrate non-inferiority of Vi-DT compared to Typbar TCV® as measured by that Geometric Mean Titers (GMT) of anti-Vi IgG at 4 weeks after single dose (pooled immunogenicity of three lots of Vi-DT)
- Describe seroconversion rates of anti-Vi IgG ELISA antibody titers, 24 weeks after single dose (pooled immunogenicity of three lots of Vi-DT) for Vi-DT and Typbar TCV®
- Describe that Geometric Mean Titers (GMT) of anti-Vi IgG at 24 weeks after single dose (pooled immunogenicity of three lots of Vi-DT) for Vi-DT and Typbar TCV®
- Demonstrate the equivalence of immunogenicity as measured by seroconversion rates of anti-Vi IgG ELISA antibody titers of three lots of Vi-DT vaccine 4 weeks after single dose.
- Demonstrate non-inferiority of Vi-DT compared to Typbar TCV® as measured by seroconversion rates of anti-Vi IgG ELISA antibody titers, 4 weeks after single dose (pooled immunogenicity of three lots of Vi-DT) in each age strata
- Demonstrate non-inferiority of Vi-DT compared to Typbar TCV® as measured by that Geometric Mean Titers (GMT) of anti-Vi IgG at 4 weeks after single dose (pooled immunogenicity of three lots of Vi-DT) in each age strata
- Demonstrate the immunological non-interference of Vi-DT with MMR (primary) vaccine compared to MMR (primary) vaccine alone in children of 9-15 months of age (additional group only)

- Assess and describe the immunological non-interference of Vi-DT/ Typbar TCV® with MR (primary) vaccine in children of 9-15 months of age
- Assess and describe the immunological non-interference of Vi-DT with and without MR/MMR (primary) vaccine in children of 9-15 months of age
- Assess and describe safety profile in all age strata combined and in each age strata, 4 weeks after single dose
- Assess and describe safety profile in all age strata combined and in each age strata, 24 weeks after single dose

Exploratory

- Assess the number of typhoid cases in both vaccine groups

Methodology

A multicenter, randomized, observer-blinded, controlled, non-inferiority study of a single dose of SK bioscience Vi-DT (Test) compared to Vi-TT (Typbar TCV®, Bharat Biotech)(Comparator). The vaccines will be administered to 1,800 healthy participants of 6 months to 45 years of age. The participants will be followed up for safety and immunogenicity for 4 and 24 weeks post single dose of the Test and Comparator vaccines. Three lots of Vi-DT will be tested to assess lot-to-lot consistency (equivalence). The primary objective is to demonstrate the non-inferiority of pooled Test vaccine to Comparator vaccine in terms of seroconversion rates as measured by anti-Vi IgG ELISA antibody titers, 4 weeks after a single dose of Test/Comparator vaccine and to assess the equivalence of 3 lots of Vi-DT 4 weeks after single dose of Test vaccine in terms of immunogenicity as measured by anti-Vi IgG GMT for anti-Vi IgG titer. A descriptive evaluation of safety and immunogenicity at 24 weeks post single dose, will also be performed.

The Vi-DT vaccine will be administered at a single dose of 25 µg.
Eligible participants enrolled into the study will be randomized into one of the four study groups within each age strata of 6 months to less than 2 years, 2 to less than 18 years and 18 to 45 years. Participants will be observed at the study site for 30 minutes after vaccination for safety assessment. Solicited adverse events will be recorded on a diary card during 7 days after vaccination. Unsolicited adverse events will be recorded during the 4 weeks after vaccination. Serious adverse events will be recorded during the entire study period. With the exception of designated study site personnel responsible for vaccine administration, study investigators, study nurse, and those assessing clinical outcomes, and data analysts will be blinded to vaccine allocation until data base lock for the final analysis.
A primary analysis will be performed after all participants complete week 4 visit post Test/Comparator vaccine dose in order to initiate the test vaccine licensure process. Immunogenicity and safety data up to week 4 will be included in this analysis and this will be done in a way so that the study and study personnel remain blinded to the allocation of test/comparator vaccine until the end of the study. The final analysis will be performed when all participants complete week 24 visit. Immunogenicity and safety data up to week 24 will be included in the final analysis.
Blood samples will be collected at baseline prior to vaccination and at 4 and 24 weeks post vaccination for immunogenicity assessment.

Additional group:

In addition to the 1800 participants, sites will recruit additional 360 participants in the age group of 9-15 months. Study design for additional group will be open label in which MMR recepient with or without test vaccine (Vi-DT) will be analysed for immunogenicity.<br>Eligible participants enrolled into the additional group will be randomized into one of the two study groups (Vi-DT+MMR or MMR alone). Participants will be observed at the study site for 30 minutes after vaccination for safety assessment. Solicited adverse events will be recorded on a diary card during 7 days after vaccination. Unsolicited adverse events and serious adverse events will be recorded during the 4 weeks after vaccination. This additional group of participants will be assessed for immune non-interference of Vi-DT with Measles, Mumps and Rubella at 4 weeks post vaccination.Participants in the MMR alone arm will be offered locally licensed Typbar TCV® as a benefit vaccine 4 weeks post MMR vaccination.
**Estimated Number of participants to Enroll**<br><br>A total of 1,800 participants aged 6 months to 45 years will be enrolled in this study. Participants will be randomized equally into 4 groups of 450 participants each, within each age strata of 6 months to less than 2 years, 2 to less than 18 years and 18 to 45 years. Participants in the first three groups will receive a single dose of Test vaccine from any of the 3 lots, while the fourth group will receive the Comparator vaccine. Participants will be given one dose of MR (primary) at the age of 9 to 15 months.<br>**Additional group:**<br>In addition to the 1800 participants, sites will recruit additional 360 participants in the age group of 9-15 months and will be given one dose of locally licensed MMR (primary) vaccine with or without test vaccine
**Criteria for Inclusion/Exclusion**<br><br>**Inclusion Criteria**<br>In order to be eligible to participate in this study, any individual must meet the following criteria:<br>• Healthy participants 6 months to 45 years of age at enrollment<br>• Healthy participants 9 to 15 months of age at enrollment (additional group only)<br>• Participants/Parents/LAR who have voluntarily given written informed consent/assent<br>• Participants/Parents/LAR willing to follow the study procedures of the study and available for the entire duration of the study<br><br>**Exclusion Criteria**<br>An individual who meets any of the following criteria will be excluded from participation in this study:<br>• Child with a congenital abnormality<br>• Subject concomitantly enrolled or scheduled to be enrolled in another trial

- Known history of immune function disorders including immunodeficiency diseases (Known HIV infection or other immune function disorders)
- Chronic use of systemic steroids (>2 mg/kg/day or >20 mg/day prednisone equivalent for periods exceeding 10 days), cytotoxic or other immunosuppressive drugs
- Receipt of blood or blood-derived products in the past 3 months
- Subject with a previously ascertained or suspected disease caused by *S. Typhi*
- Subject who have had household contact with/and or intimate exposure to an individual with laboratory-confirmed *S. Typhi*
- Individual who has previously received a typhoid vaccine
- Subject who has received or is expected to receive other vaccines from 1 month prior to IP vaccination to Visit 4 (approx.1 month post IP) except PCV booster as per EPI schedule or any vaccine during National Immunization catch-up campaign of Nepal
- Known history or allergy to vaccines or other medications
- History of uncontrolled coagulopathy or blood disorders
- Any abnormality or chronic disease which in the opinion of the investigator might be detrimental for the safety of the subject and interfere with the assessment of the study objectives
- Any female participant who is lactating, pregnant* or planning for pregnancy during the course of study period
- Participants/Parents/LAR planning to move from the study area before the end of study period
- As per Investigator's medical judgement individuals could be excluded from the study in spite of meeting all inclusion/exclusion criteria mentioned above

Temporary Contraindication

- Acute illness, in particular infectious disease or fever (axillary temperature ≥37.5°C), within three days prior to enrollment and vaccination.
  These individual could be rescreened upon resolution of the above said conditions.

*Urine pregnancy test (UPT) will be performed in all married females

Investigational Product, Dosage and Mode of Administration:

Test Vaccine

Vi polysaccharide typhoid vaccine conjugated with Diphtheria toxoid protein (Vi-DT), manufactured by SK bioscience (Republic of Korea).

- Dose formulation: 25 µg Vi polysaccharide /0.5 mL, presented in multiple dose glass vial
- Mode of Administration: 0.5 mL by intramuscular injection in the left anterolateral thigh or left arm deltoid region in participants below 2 years of age, left arm deltoid region in age group 2-45 years
- Storage Conditions: +2-8°C

Comparator Vaccine

Vi polysaccharide typhoid vaccine conjugated with Tetanus toxoid protein (Vi-TT),

Typbar TCV®, manufactured by Bharat Biotech (India)

- Dose formulation: 25 µg Vi polysaccharide /0.5 mL, presented in single dose glass vials
- Mode of Administration: 0.5 mL by intramuscular injection in the left anterolateral thigh or left arm deltoid region in participants below 2 years of age, left arm deltoid region in age group 2-45 years
- Storage Conditions: +2-8°C

Group Allocation

	Age group	N	D0
**Group A**<br>(6 mo - 45 yrs)<br>450 participants	6 mo-<2 yrs	150	(25 µg 0.5 mL)<br>(Vi-DT)<br>Test Lot 1
	2- <18 yrs	150	
	18-45 yrs	150	
**Group B**<br>(6 mo - 45 yrs)<br>450 participants	6 mo-<2 yrs	150	(25 µg 0.5 mL)<br>(Vi-DT)<br>Test Lot 2
	2- <18 yrs	150	
	18-45 yrs	150	
**Group C**<br>(6 mo - 45 yrs)<br>450 participants	6 mo-<2 yrs	150	(25 µg 0.5 mL)<br>(Vi-DT)<br>Test Lot 3
	2- <18 yrs	150	
	18-45 yrs	150	
**Group D**<br>(6 mo - 45 yrs)<br>450 participants	6 mo-<2 yrs	150	25 µg 0.5 mL<br>(Typbar TCV®)
	2- <18 yrs	150	
	18-45 yrs	150	
			MR for age eligible (9-15 months) participants

Additional group:

	Age group	N	D0
Group E	9-15 months	180	(25 µg 0.5 mL) (Vi-DT) + MMR
Group F	9-15 months	180	MMR

Criteria for Evaluation

Primary Endpoints

- Seroconversion rate (seroconversion is defined as a 4-fold increase of serum anti-Vi IgG antibody titer) at 4 weeks (28 days) after vaccination of Vi-DT(pooled)/ Typbar TCV® compared to baseline (D0).
- Geometric Mean Titers (GMT) of anti-Vi IgG at 4 weeks (28 days) after vaccination of three lots of Vi-DT.

Secondary Endpoints

- Geometric Mean Titers (GMT) at 4 weeks (28 days) and 24 weeks(168 days) after single dose of Vi-DT (pooled)/ Typbar TCV®
- Seroconversion rates at 24 weeks (168 days) after single dose of Vi-DT (pooled)/ Typbar TCV®
- Seroconversion rates of anti-Vi IgG ELISA antibody titers at 4 weeks (28 days) after vaccination of three lots of Vi-DT.
- Seroconversion rates of anti-Vi IgG ELISA antibody titers at 4 weeks (28 days) after vaccination of three lots of Vi-DT in each age strata
- IgG ELISA antibody titers for Measles (M), Mumps (M) and Rubella (R) following single dose of MMR vaccine at baseline D0 and 4 weeks (28 days after single dose of MMR). The derived endpoints are seropositive rates:
    - Sero-positive rate for anti-Measles: ≥ 275 IU/L
    - Sero-positive rate for anti-Mumps: ≥ 22 RU/mL
    - Sero-positive rate for anti-Rubella: ≥ 11 IU/mL

(For addition group, only 1 lot of Vi-DT will be used & Mumps component is applicable to additional group only))

- Local and systemic solicited adverse events during the 7 days after vaccination
    - Solicited local reactions at the site of injection: Pain/ tenderness, erythema/redness, swelling/ induration, pruritus associated with injection
    - Solicited Systemic reactions (adapted to each age group): Fever,

      headache,fatigue, myalgia,lethargy, irritability, nausea, vomiting,arthralgia,

diarrhea, drowsiness, loss of appetite, chills, rash,nasopharyngitis and persistent crying

- Unsolicited adverse events during 4 weeks after vaccination
- Serious Adverse Events during the entire study period

Exploratory endpoints

- Number of typhoid cases in both the groups (fever ≥38˚C persisting for ≥72 hours and other diagnosis for fever cause ruled out, followed by positive blood culture for *S*. Typhi.)

Statistical Considerations

The statistical analysis will focus on comparisons of immunogenicity of Vi-DT (Groups A, B, C combined) and Typbar TCV® (Group D) and to test lot-to-lot consistency of Vi-DT (Comparison between Groups A, B and C).

The sample size of the study is calculated to provide about 90% power to show primary objectives of the study, and increased to satisfy total safety population of about N=1,350. The given sample size of N=450 participants per vaccine group would provide 99% power to detect the non-inferiority of seroconversion rate of Vi-DT (combined Groups A+B+C) compared to Typbar TCV® (Group D). This sample size will also provide 97% power for three equivalence tests of GMT ratio of immunogenicity among three lots of Vi-DT (A vs. B, B vs. C and C vs. A) with overall two-sided significance level of 0.025. Each two-sided equivalence test has 97% power to show the equivalence of GMT of immunogenicity of Vi-DT with two-sided significance level of 0.0083 each. The equivalence margin of ratio is assumed as [0.67, 1.5] (WHO TRS 924), the true GMT ratio is assumed as 1 and the coefficient of variation on titer of immunogenicity is assumed as 2.0. In both sample size calculations, a 15% drop out rate is assumed conservatively considering the potential large variation of experience between sites.

Additional group:

The sample size of N=360 (a 1:1 ratio for groups) is calculated to provide 80% power to detect the non-inferiority of seropositive of IgG antibody titers for Measles (M), Mumps (M) and Rubella (R) following single dose of MMR with Vi-DT (Group E) compared to MMR vaccine alone (Group F) at 2.5% one-sided significance level. The non-inferiority margin is assumed as 10%, the true seropositive rate of anti-Measles, anti-Mumps and anti-Rubella antibody titers is assumed as 90%, and a 10% drop out rate is considered.

Primary Comparison

To demonstrate Non-inferiority of Vi-DT vaccine compared to Typbar TCV®

- Anti-Vi IgG seroconversion rate at 4 weeks (day 28) post Vi-DT vaccine (25 μg) (pooled Groups A+B+C) is non-inferior to seroconversion rate at 4 weeks post Typbar TCV®

(Group D) using non-inferiority margin of 10%

To demonstrate the lot-to-lot consistency of Vi-DT vaccine

- Anti-Vi IgG GMT at 4 weeks(day 28) post each lot of Vi-DT vaccine is equivalent to each other (A vs. B, B vs. C, and C vs. A) using equivalence margin of GMT ratio of [0.67, 1.5]

To preserve the study-wise type 1 error rate 0.05, Bonferroni methods will be utilized to control the multiple testing procedure. The non-inferiority test of Vi-DT vs. Typbar TCV® will be performed as 0.0125 significance level using one-sided test and three equivalent tests will be performed as 0.025 overall significant level using two-sided test (each of two sided equivalent test will be tested with 0.0083 significant level)

Secondary Comparisons

- Geometric Mean Titers (GMT) of anti-Vi IgG at 4 weeks(day 28) post vaccination of Vi-DT (Groups A+B+C) is non-inferior to Typbar TCV® (Group D) using non-inferiority margin of GMT ratio ≥ 0.67

- Anti-Vi seroconversion rate at 4 weeks (day 28) post each lot of Vi-DT vaccine is equivalent to each other (A vs. B, B vs. C, and C vs. A) using equivalence margin of difference of [-10%, 10%]

- Sero-positive rate of IgG antibody titers for Measles (M),Mumps (M) and Rubella (R) following single dose of MMR at 4 weeks in participants who received MR + Vi-DT is non-inferior to sero-positive rate in participants who received MMR alone with non-inferioirty margin of 10% (additional group only)

The following secondary immunogenecity endpoints will be descriptively summarized by each group and each age strata.

- Anti-Vi seroconversion rate at 24 weeks(day 168) post Vi-DT vaccine (25 µg) (Groups A+B+C) and Typbar TCV® (Group D)

- Geometric Mean Titers (GMT) of anti-Vi IgG at 24 weeks (day 168) post vaccination of Vi-DT (Groups A+B+C combined) and Typbar TCV® (Group D)

- Anti-Vi IgG seroconversion rate and GMT at 4 weeks (day 28) post Vi-DT vaccine (25 µg) (pooled Groups A+B+C) and Typbar TCV® (Group D) in participants who received concomitantly MR vaccine

- Anti-Vi IgG seroconversion rate and GMT at 4 weeks (day 28) post Vi-DT vaccine (25 µg) (pooled Groups A+B+C) in participants 9-15 months who concomitantly received

and did not receive MR vaccine

- Sero-positive rate of IgG antibody titers for Measles (M) and Rubella (R at 4 weeks (day 28) post vaccination of Vi-DT (Groups A+B+C combined) and Typbar TCV® (Group D) in participants who received concomitant MR vaccine

- Anti-Vi IgG seroconversion rate and GMT at 4 weeks (day 28) post Vi-DT vaccine (25 µg) (pooled Groups A+B+C) in participants 9-15 months who concomitantly did not receive MR vaccine and Group E who received MMR

The following safety endpoints will be descriptively summarized by each group and each age strata.

- Frequency of local and systemic solicited adverse events during the 7 days after each dose
    - Solicited local reactions at the site of injection: Pain / tenderness, erythema/redness, swelling/ induration, pruritus associated with injection
    - Solicited Systemic reactions: Fever, headache,fatigue, myalgia,lethargy, irritability, nausea, vomiting,arthralgia, diarrhea, drowsiness, loss of appetite, chills, rash, nasopharyngitis and persistent crying

- Frequency of unsolicited adverse events during 4 weeks after vaccination

- Frequency of Serious Adverse Events during the entire study period

2 INTRODUCTION

2.1 BACKGROUND

Typhoid fever is one of the most common causes of bacteremia in several low- and middle-income countries (LMIC) and has been estimated to cause 11- 21 million cases and 145,000-161,000 deaths per year [1]. Typhoid fever is more common in children and young adults than in older people [2]. Worldwide, typhoid fever is most prevalent in impoverished areas that are overcrowded with poor access to sanitation. Incidence estimates suggest that south-central Asia, Southeast Asia, and southern Africa are regions with high incidence of *S*. Typhi infection (more than 100 cases per 100,000 person years) [3,4,5]. Other regions of Asia and Africa, Latin America, the Caribbean, and Oceania have a medium incidence of 10 to 100 cases per 100,000 person years. These estimates, however, are limited by lack of consistent reporting from all areas of the world and are based on extrapolation of data across regions and age groups. Recent data from Africa have revealed that several countries in Eastern and West Africa have rates >100 per 100,000 [6].

In Nepal, typhoid fever is also known as "bisham joauro" meaning fever with poison and people in Nepal are at high risk of Typhoid [7]. The Typhoid is prevalent all over the Nepal from east to west including Terai, Hill and Mountain region, however, Kathmandu valleys and southern belts are considered as endemic areas with peak incidence in May to August and Kathmandu, the capital city of Nepal, has previously been coined the typhoid fever capital of theworld [7]. Enteric fever is endemic in Nepal and *S*. Typhi and *S*. Paratyphi A are the most commonly isolated organisms from the blood of febrile patients in Kathmandu-based healthcare setting [8]. A retrospective analysis highlighted a substantial burden of enteric fever within the local population, particularly in school-age children and males aged 15 to 25 years [9]. A total of 9124 Typhoid cases were confirmed by blood culture from Kathmandu during 1993-2003 [10].

Etiological agent

Typhoid fever is caused by *Salmonella enterica* serovar Typhi (*S*. Typhi). It is a rod-shaped gram-negative facultative anaerobe bacterium belonging to the Enterobacteriaceae family. Among more than 2,300 closely-related Salmonella serovars recognized, *Salmonella enteritica* serotype Typhi and *Salmonella enterica* serotype Paratyphi A, B & C are pathogenic exclusively for humans. Infection therefore implies contact with infected person or use of contaminated food

or water. Non typhoidal salmonella (such as *Salmonella enteritidis* and *Salmonella typhimurium* may also cause sever illness consistent with typhoid fever [10]. *Salmonella* possesses a flagellar antigen (H), somatic (O) and a surface antigen (Vi). The Vi capsular antgen is a superficial overlying antigen. It is present in a few serovars, the most important of which is *Salmonella enteritica* serotype Typhi but is also present in *Salmonella enteritica* serotype Paratyphi C and *Salmonella dublin*.

Clinical presentation

Typhoid fever is one of the most common causes of bacteremia in many developing countries [11]. The clinical feature of typhoid fever is that of a sub-acute systemic infection. Classic reports describe the characteristic stages of typhoid fever in untreated individuals with rising fever and bacteremia. However, presentation is variable ranging from mild fever to more severe forms such as toxic shock. General symptoms include high grade fever (40°C) lasting for more than 3 days, profuse sweating, chills, abdominal pain, altered bowel functions, malaise, myalgia, anorexia, intestinal bleeding and perforation [12]. In a small percentage of cases, the bacteria may also colonize the gall bladder, leading to a chronic carrier state. Although the disease is known widely, typhoid fever is still often confused with other acute febrile illnesses such as malaria, typhus and dengue fever, even with the use of laboratory diagnosis.

Pathogenesis

Susceptible human hosts are infected upon consumption of food or water contaminated with *S.* Typhi. Inside the small intestine, *S.* Typhi attach to epithelial cells, penetrates the mucosal epithelium to reach the lamina propria through enterocytes and M cells, the domelike epithelial cells that cover Peyer's patches. In the lamina propria, *S*. Typhi triggers an influx of macrophages and dendritic cells that ingest the bacilli but do not generally kill them. Some remain within macrophages of the small intestinal lymphoid tissue. Other typhoid bacilli are drained into mesenteric lymph nodes where there is further multiplication and ingestion by the macrophages. Eventually, there is a release of tumor necrosis factor-alpha, interleukin-2 (IL-2), IL-6, and other inflammatory cytokines by the mononuclear cells. After reaching the blood circulation via the thoracic duct, the bacteria are filtered from the circulation and sequestered inside the phagocytic cells of the liver, spleen, and bone marrow [13]. Replication within the endothelial system is the hallmark of typhoid fever and is responsible for the clinical finding of prostration, generalized sepsis and hepato-splenomegaly. Some individuals will contain the

organmnism within the gastrointestinal system and do not become systematically ill but have persistent *S.* Typhi carriage [14].

Typhoid control and prevention

Most of the typhoid cases are effectively treated with antibiotics, although the case fatality rate remains at about 1%. Improvement in sanitary infrastructures and implementation of hygienic practices can reduce the typhoid disease burden as seen in most developed countries. However, the development of adequate infrastructures for improved water and sanitation requires large and long-term investments, and is therefore a distant goal for impoverished populations. Instead, increased population and limited opportunities in rural areas has resulted in urbanization and increased population density, the major risk factor for typhoid transmission. Basic health education such as hand washing and proper food handling is also known to be effective in reducing typhoid fever. Although typhoid fever can be effectively treated with antibiotics, growing rates of antibiotic resistance in many countries are making this treatment option increasingly more difficult and costly.

Though a vaccine against typhoid fever was developed and used in the early 20th century, typhoid vaccine development received attention in the early 1960s, when *S.* Typhi strains resistant to chloramphenicol were isolated. As a result, among many candidates, two vaccines, one oral, and one injectable, were licensed in 1990. Today, there is enough evidence that typhoid fever vaccines are efficacious, effective under public health conditions, and have an impact on the incidence for the benefit of larger population. WHO has recommended that countries consider the use of typhoid vaccines for high-risk groups and populations, and for outbreak control [1]. It is therefore essential to consider a comprehensive approach that combines targeted vaccination of high-risk populations as a short-to medium-term prevention measure, along with longer term solutions of water and sanitation improvements and improved living standards [15]. In endemic countries, control of typhoid would require implementing immunization for young children and incorporating typhoid vaccine in the EPI.

There are currently three WHO-recommended vaccines for protection agaiinst *S.* Typhi: live oral vaccine strain Ty21a,parentral Vi polysaccharide vaccines and typhoid conjugate vaccine, Typbar TCV® [1].

Live, Oral Ty21a Vaccine

The Ty21a vaccine consists of a mutant strain of Salmonella Typhi Ty2 that was isolated after chemical mutagenesis and has a galE- and Vi-negative phenotype. It is supplied in enteric -coated capsules, or Liquid suspension (lyophilized vaccine + buffer mixed with water upon use). Immune response to the vaccine starts 14 days after vaccination, which is mediated by mucosal (IgA), serum (IgG), and cell-mediated antibodies. The vaccine has showed no booster effect. It has shelf life of 14 days at +25°C.

The overall protective efficacy for a three-dose regimen ranged between 67% and 80% in large-scale efficacy trials, conducted in 1980s in Chile [16]. The most common adverse events reported with Ty21a were mild and transient gastrointestinal disturbances, followed by general symptoms such as fever. This vaccine is licensed for use in persons 2 years and above for the liquid formulation and 5 years and older for the capsule formulation.

Parenteral Vi Polysaccharide Vaccine

The parenteral subunit Vi polysaccharide vaccine (ViPS) was developed from wild type *S.* Typhi strain Ty2 on the basis of non-denatured purification of the Vi polysaccharide at the National Institute of Health (US). The ViPS vaccine is given as a single dose and was found to confer, overall, 64–72% protection for 17–21 months and 55% over 3 years [17]. The ViPS vaccine is well tolerated and safe. The most common side effects are pain, redness and induration at injection site, and fever. The Vi capsular polysaccharide synthesized by *S*. Typhi is an important virulence determinant and the ability to produce antibodies against Vi is a critical component in the host's defense against infection by *S*. Typhi. ViPS vaccine was found to be poorly immunogenic in children 2-5 years and not immunogenic in children < 2 years of age. This vaccine continues to be the most common vaccine in use in high endemic countries and was systematically used in routine public health programs in China, Vietnam and Nepal, The vaccine is widely available in the private market in China, India, Pakistan and many other endemic countries. Few counties such as Sri Lanka use this vaccine in their public health program through targeted approach; otherwise, no other country adopted the vaccine in their immunization program.

Typhoid conjugate vaccines

The scientists at the US National Institute of Child Health and Disease (NICHD) have developed the method that used the heterobifunctional cross-linking reagent, N-succinimidyl-3-(2-pyridyldithio)-propionate (SPDP) or adipic acid dihydrazide (ADH) as linkers to bind Vi to proteins. Using a nontoxic recombinant protein that is antigenically identical to *Pseudomonas aeruginosa* exotoxin A as a carrier protein, the resultant conjugates (Vi-rEPA) were more immunogenic in mice and juvenile Rhesus monkeys than the Vi alone. In contrast to the T-independent properties of the Vi alone, conjugates of this polysaccharide with several medically relevant proteins induced booster responses in mice and in juvenile Rhesus monkeys. This synthetic scheme was reproducible, provided high yields of Vi-protein conjugates, and was applicable to several medically relevant proteins such as diphtheria and tetanus toxoids [18]. In a randomized, vaccine-controlled study of infants in Vietnam, Vi-rEPA was safe, elicited protective levels of IgG anti-Vi, and was compatible with EPI vaccines. These data show that Vi-rEPA can be added to the routine immunization of infants in countries where typhoid fever is prevalent [19].

The Novartis Vaccines Institute for Global Health, Siena, Italy, is developing a typhoid conjugate vaccine (Vi-CRM197) using Vi from *Citrobacter freundii* WR7011 conjugated to the, CRM197, a non-toxic mutant of the diphtheria toxin [20]. Phase I and II clinical trials were conducted in European adults. In the phase I trial, single dose of Vi-CRM197 was compared with Typherix® in 50 European volunteers between 18 to 40 years of age. Phase II trial was a dose-ranging design (12.5, 5.0 or 1.25 µg) with 88 European participants between 18 to 40 years of age in which all Vi-CRM197 doses were at least as immunogenic as unconjugated Vi [21]. Recently, phase II studies have been completed in India, Pakistan, and The Philippines in four different age-groups: 18 to 45 years; 24 to 59 months; 9 to 12 months; and infants aged 6 weeks with each group having 40 participants. Novartis Vaccines Institute for Global Health (NVGH, now Scalvo Behring Vaccines Institute for Global Health, GSK company) since then has transferred the technology to Biological E, Hyderabad, India.

With the technology initially transferred from US NIH, Biomed Pvt Ltd. in India developed a conjugate vaccine using Tetanus Toxoid as the carrier protein. This product was tested in a clinical trial in 169 participants > 12 weeks with a comparison group (Vi) of 37 children > 2 years[22] . The results from this study were compared with the NIH study in Vietnam and it was reported that there was four fold or greater rise in antibody titer of each group on ELISA which

was statistically equivalent to Vi-rEPA. Based on the results of this study, this product was submitted for licensure and was licensed for more than 3 months of age in 2008 in India.

Similarly, Bharat Biotech in Hyderabad, India also developed typhoid conjugate vaccine using Tetanus Toxoid as the carrier protein with Vi polysaccharide. This vaccine was tested in children (2 to 17 years) for safety, immunogenicity and dose ranging (15 µg versus 25 µg/0.5 mL). There was no significant difference between two doses of 25 µg/ 0.5 mL and two doses of 15 µg/0.5 mL. In the next clinical trial, comparative assessment of the immunogenicity of Vi-TT versus the polysaccharide vaccine was done in 981 participants (6 months to 45 years old). The investigators found 4-fold seroconversion rates in each treatment arm at 6 weeks post vaccination [23]. After 2 years of follow-up, the anti-Vi titers were in the study arm as compared to comparator arm. Based on these results, Bharat Biotech received marketing authorization for Typbar TCV in India in 2013 as a single dose indication for all aged 6 months and above. WHO pre-qualification was awarded to Bharat Biotech for Typbar TCV® in January 2018.

3 VI-DT CONJUGATE VACCINE

The Vi-DT Vaccine is a conjugate typhoid vaccine in which purified Vi polysaccharide derived from Salmonella Typhi C6524 is conjugated to Diphtheria Toxoid (DT) as the carrier protein. The Vi-DT vaccine to be used in this trial contains 25 µg/0.5mL of Typhoid antigen (Vi) in the form of Vi-Diphtheria Toxoid conjugate, presented in multiple dose glass vial .

3.1 PRECLINICAL DATA

Pre-clinical immunogenicity and toxicity studies were conducted to assess immunogenicity and ensure safety of Vi-DT in animal models. A first immunogenicity study of Vi-DT was conducted in mice. Typhoid Kovax (Vi-polysaccharide typhoid vaccine) marketed by Korea Vaccine Co., Ltd. was used as a comparator. All animals that received Vi-DT showed higher immune responses than those elicited by the comparator. Serum antibody titers at 6 weeks were higher than at 2 weeks, demonstrating a booster effect. The antibody titers were maintained up to 10 weeks. SK bioscience also used guinea pigs and rabbits for immunogenicity testing. The result showed that Vi-DT elicited higher immune responses than those elicited by the comparator in

both animal species.

Toxicology studies for Vi-DT with single and repeat dose in mice, respiratory system toxicity in mice and cardiovascular toxicity in female beagle dogs were done at MPI Research (Mattawan, MI, USA). Study results indicated that both single and four repeat intramuscular doses (days 1, 15, 29 and 43) of Vi-DT to mice did not result in mortality, clinical or macroscopic observations, or elicit any changes in body weight, food consumption, neurobehavioral measures, or respiratory function. Also respiratory system toxicity and cardiovascular toxicity studies in mice and beagle dogs did not indicate any toxicity with Vi-DT.

3.2 CLINICAL DATA

A first-in-human Phase 1 trial was conducted in the Philippines to assess the safety and immunogenicity of Vi-DT Conjugate Vaccine compared to Vi-Polysaccharide (Typhim Vi®, Sanofi Pasteur) Typhoid Vaccine in healthy Filipino adults and children. The protocol was approved by the Research Institute of Tropical Medicine (RITM) and IVI IRBs and by the Philippines FDA. Informed consent was obtained from all participants.

A total of 144 participants were recruited in Manila, The Philippines, and randomized equally (N=72 in each group) to Test (Vi-DT) and Comparator (Typhim Vi®) group within each stratum of adults, adolescents and young children (N=48 in each strata). There was no significant difference in age and gender among test and comparator group. Male and female participants were 66% and 34%, respectively. The median age was 26 years (18-45) in adults, 11 years (6-16) in adolescents and 4 years (2-5) in children [24].

No SAE was reported in either group. No participant was discontinued from the study due to AE. All solicited and unsolicited AEs were mild or moderate in both arms with the exception of a 4-year old girl, in Test group with grade 3 fever that resolved without sequel. The proportions of participants with solicited AEs in Test and Comparator groups were respectively 54.17% and 50% in adults, 37.5% and 45.8% in adolescents and 25% and 25% in children. The proportions of participants with unsolicited AEs in Test and Comparator groups were respectively 50% and 45.8% in adults, 37.5% and 45.8% in adolescents, and 79.17% and 70.83% in children. The majority of solicited AEs in adults were pain, tenderness and headache; in adolescents pain and tenderness; and in children pain and fever [24].

All participants in Test group showed seroconversion (defined as 4-fold increase of anti-Vi IgG titer) after the first and second doses while 97% of participants in Comparator group. Test group showed about 4-fold higher GMT than in the Comparator group. SBA seroconversion rates were significantly higher in the Test group than in the Comparator group post first and second doses (71% vs. 52.17% and 70.4% vs. 51.39%, respectively). SBA GMT were also significantly higher in the Test group than in the Comparator group post first and second doses (526.56 vs. 271.26 and 586.5 vs. 222.97, respectively). Anti-DT responses were significantly higher in the Test group than in the Comparator group with a 26-fold rise post first dose compared to baseline values in the Test group while a 0.93-rise was observed in the Comparator group [24].

A Phase 2 clinical trial is being conducted in the Philippines to assess the safety and immunogenicity of Vi-DT Conjugate in healthy Filipino infants and toddlers 6-23 months of age at the time of the first vaccine dose. The protocol was approved by the RITM and IVI IRBs and by the Philippines FDA. Informed consent was obtained from all participants.Results from the trial are available as Interim report in the the attached appendix b.

3.3 STUDY RATIONALE

The Vi capsular polysaccharide synthesized by *S*. Typhi is an important virulence determinant and the ability to produce antibodies against Vi is a critical component in the host's defense against infection by *S.* Typhi. Vaccination with Vi polysaccharide has been shown to protect individuals from typhoid fever but Vi vaccine has a number of limitations. Vi is poorly immunogenic and revaccination does not elicit an anamnestic response [25]. There is increasing evidence of S. typhi infection in younger children supporting the need for vaccinating children against typhoid in the first year of life [26]. However, the response to Vi polysaccharide in children under two years of age is poor and consequently Vi vaccines are not licensed for use in this at risk age group. The limitations of Vi vaccines can be overcome by conjugation of the Vi to a carrier protein. Immune responses to bacterial capsular polysaccharides are generally T-cell independent and lack affinity maturation, poor antibody subclass switching and the inability to generate memory. Conjugation of the polysaccharide to a protein carrier converts the immune response to T-cell dependent, which is characterized by affinity maturation, subclass switching and induction of memory.

Since the major burden of typhoid fever is borne by pre-school, school and young children [5,27] and increasing evidence of significant burden under the age of 2 years [28] suggests the urgent need for improved typhoid vaccines in terms of better efficacy and long term immune sustainability. Availability of the locally licensed TCV vaccines in few endemic countries and recent WHO pre-qualification of Typbar TCV® is a major step forward in this direction. Recent availability of Gavi funds may help in overcoming funds problems and ensuring incorporation of TCV in vaccination programs for Gavi eligible countries. To meet the global demand of TCV and to make it more affordable there is thus a need for more TCV vaccine candidates in market. IVI and SK bioscience with funding support from BMGF are developing a typhoid conjugate vaccine composed of Vi polysaccharide conjugated to diphtheria toxoid (Vi-DT) has been developed.

Most of typhoid conjugate vaccines tested in humans were administered at the dose of 25 µg. Vi-DT typhoid conjugate vaccine developed by SK bioscience (SK) was also tested at the dose of 25 µg in a Phase I clinical trial of 144 participants enrolled in the Philippines. As described in section 3.2, results from the same study were favorable and paved the way for a Phase II clinical trial.

Phase II trial in Filipino infants and toddlers 6-23 months of age is going on and post dose 1 results establishes the safety and immunogenicity of the Vi-DT in below two age group (6-23 months). For results, refer to Appendix b.

Phase 1 and 2 studies have demonstrated the safety and immunogenicity of Vi-DT test typhoid conjugate vaccine in limited number of participants aged 6 months to 45 years, and same need to be established in large statistically powered phase 3 study. This study will satisfy two criteria, immune non-inferiority of Vi-DT to locally licensed typhoid conjugate vaccine (Vi-TT or Typbar TCV®, Bharat Biotech) and equivalence of immunogenicity of three lots of Vi-DT. For age eligible participants, possible interference with EPI vaccines (MR) will be assessed.

Additional Group: Due to the government organized MR catch-up campaign from February 2020 and COVID 19 travel restrictions from March 2020, required number of MR recepients could not be secured in the main study group. To meet the secondary objective of Immune Non-interference of Vi-DT with MR, additional group has been proposed.MMR has been proposed for this additional group instead of MR beause of unavailability of locally licensed MR vaccine with long expiry date(atleast 6 month)

3.4 POTENTIAL RISKS AND BENEFITS

3.4.1 KNOWN POTENTIAL RISKS

Vi conjugate vaccines already licensed in India are found to be safe [23]. Also the Vi-DT vaccine components, Vi Polysaccharide, DT and preservative 2-PE, are licensed and in use for a long time with established safety profile. In the course of the Phase 1 and 2 trial conducted in the Philippines at RITM, Vi-DT was not associated with any severe or serious adverse event . Any vaccine could cause an anaphylactic reaction, though such reactions are rare. Expected local and systemic reactions include injection site Pain/ tenderness, erythema/redness, swelling/ induration, pruritus associated with injection and systemic reactions such as Fever, headache,fatigue, myalgia,lethargy, irritability, nausea, vomiting,arthralgia, diarrhea, drowsiness, loss of appetite, chills, , rash, nasopharyngitis and persistent crying. These side effects are expected to be mild or moderate and transient and resolving spontaneously without sequelae.

3.4.2 KNOWN POTENTIAL BENEFITS

Study participants may receive no direct benefit from study participation. They will however have access to their medical records. Findings of medical concern will be referred for appropriate care and treatment. Compared with polysaccharide vaccines, conjugate vaccines are usually more immunogenic and better at inducing long term memory responses, especially in young children < 2 years of age. The potential benefits to vaccinated participants are substantial, since typhoid is endemic in several parts of the world. Typhoid affects infants, young children and adults which can lead to death if not treated promptly with appropriate medical care including antibiotics. The use of an effective vaccine in all age groups will contribute to the prevention and control of typhoid and prevent death from typhoid fever.

4 OBJECTIVES

Primary

- Demonstrate non-inferiority of Vi-DT compared to Typbar TCV® as measured by seroconversion rates of anti-Vi IgG ELISA antibody titers, 4 weeks after single dose (pooled immunogenicity of three lots of Vi-DT)
- Demonstrate the equivalence of immunogenicity as measured by anti-Vi IgG GMT of three lots of Vi-DT vaccine 4 weeks after single dose.

Secondary

- Demonstrate non-inferiority of Vi-DT compared to Typbar TCV® as measured by that Geometric Mean Titers (GMT) of anti-Vi IgG at 4 weeks after single dose (pooled immunogenicity of three lots of Vi-DT)
- Describe seroconversion rates of anti-Vi IgG ELISA antibody titers, 24 weeks after single dose (pooled immunogenicity of three lots of Vi-DT) for Vi-DT and Typbar TCV®
- Describe that Geometric Mean Titers (GMT) of anti-Vi IgG at 24 weeks after single dose (pooled immunogenicity of three lots of Vi-DT) for Vi-DT and Typbar TCV®
- Demonstrate the equivalence of immunogenicity as measured by seroconversion rates of anti-Vi IgG ELISA antibody titers of three lots of Vi-DT vaccine 4 weeks after single dose.

- Demonstrate non-inferiority of Vi-DT compared to Typbar TCV® as measured by seroconversion rates of anti-Vi IgG ELISA antibody titers, 4 weeks after single dose (pooled immunogenicity of three lots of Vi-DT) in each age strata
- Demonstrate non-inferiority of Vi-DT compared to Typbar TCV® as measured by that Geometric Mean Titers (GMT) of anti-Vi IgG at 4 weeks after single dose (pooled immunogenicity of three lots of Vi-DT) in each age strata
- Demonstrate the immunological non-interference of Vi-DT with MMR (primary) vaccine compared to MMR (primary) alone in children of 9-15 months of age (additional group only)
- Assess and describe the immunological non-interference of Vi-DT/ Typbar TCV® with MR (primary) vaccine in children of 9-15 months of age
- Assess and describe the immunological non-interference of Vi-DT with and without MR/MMR (primary) vaccine in use in children of 9-15 months of age
- Assess and compare safety profile in all age strata combined and in each age strata, 4 weeks after single dose
- Assess and compare safety profile in all age strata combined and in each age strata, 24 weeks after single dose

Exploratory

- Assess the number of typhoid cases in both vaccine groups

5 STUDY DESIGN

5.1 STUDY ENDPOINTS

Primary Endpoints

- Seroconversion rate (seroconversion is defined as a 4-fold increase of serum anti-Vi IgG antibody titer at 4 weeks (28 days) after vaccination of Vi-DT(pooled)/ Typbar TCV® compared to baseline (D0).
- Geometric Mean Titers (GMT) of anti-Vi IgG at 4 weeks (28 days) after vaccination of three lots of Vi-DT.

Secondary Endpoints

- Seroconversion rates at 24 weeks (168 days) after single dose of Vi-DT(pooled)/ Typbar TCV®
- Geometric Mean Titers (GMT) and Ratio of Geometic Mean titer (GMR) of Anti-Vi IgG at baseline D0 and 24 weeks (168 days) after single dose of Vi-DT(pooled)/ Typbar TCV®
- Seroconversion rates of anti-Vi IgG ELISA antibody titers at 4 weeks (28 days) after vaccination of three lots of Vi-DT
- IgG ELISA antibody titers for Measles (M), Mumps (M) and Rubella (R) following single dose of MMR vaccine at baseline D0 and 4 weeks (28 days after single dose of MMR). The derived endpoints are seropositive rates:
    - Sero-positive rate for anti-Measles: ≥ 275 IU/L
    - Sero-positive rate for anti-Mumps: ≥ 22 RU/mL
    - Sero-positive rate for anti-Rubella: ≥ 11 IU/mL

(For addition group, only 1 lot of Vi-DT will be used & Mumps component is applicable to additional group only))

- Frequency of local and systemic solicited adverse events during the 7 days after vaccination
    - Solicited local reactions at the site of injection: Pain/ tenderness, erythema/redness, swelling/ induration, pruritus pruritus associated with injection

    - Solicited Systemic reactions (adapted to each age group): Fever, headache,fatigue, myalgia,lethargy, irritability, nausea, vomiting, arthralgia, diarrhea, drowsiness, loss of appetite, chills, rash,nasopharyngitis and persistent crying
- Frequency of unsolicited adverse events during 4 weeks after vaccination
- Frequency of Serious Adverse Events during the entire study period

Exploratory endpoints

- Number of typhoid cases in both the groups (fever ≥38˚C persisting for ≥72 hours and other diagnosis for fever cause ruled out, followed by positive blood culture for *S*. Typhi.)

5.2 OVERALL DESIGN

This is a randomized, observer-blinded, Phase 3 study in healthy participants 6 months to 45 years of age at the time of the vaccine/test vaccine dose. The purpose of this study is to assess the safety and immunogenicity of the Vi-DT in age 6 month to 45 yrs in comparison to locally licensed typhoid conjugate vaccine (ie., immune non-inferiority and Equivalence among the three lots).

Participants will be evaluated at screening for general health, medical history, and undergo a physical examination. Eligible participants enrolled into the study will be randomized into one of the four study groups within each age strata of 6 months to less than 2 years, 2 to less than 18 years and 18 to 45 years.

The Vi-DT vaccine will be administered at 25 µg/0.5 mL, presented in multiple dose glass vial with preservative 2 PE. Children aged 9 months to 15 months will receive Vi-DT or Typbar TCV® along with MR , which is the recommended EPI vaccine in the country for this age group.

Safety and tolerability will be assessed with clinical monitoring. Participants will be observed at the study site for 30 minutes after each vaccination and safety assessment recorded. Adverse events including vaccine-related reactions will be solicited with the aid of diary cards and interview with participants/parents/ legal guardian after vaccination. The information gained from the review of the diary card and the interview of participants/parents/legal guardians will be recorded in the eCRF. In addition adverse events will be documented at each clinical encounter after the vaccination through 4 weeks. Serious adverse events will be recorded during the entire study period. Physical examination and vital sign will be recorded at intervals specified in schedule of events.

Blood samples will be collected at intervals specified in the schedule of events to assess immunogenicity and the possible Vi-DT, MR vaccine interaction in age-eligible participants.

Study investigators, study nurse, those assessing clinical outcomes and data analysts will be blinded to vaccine allocation with the exception of designated study site personnel responsible for vaccine administration and preparation until data base lock for the final analysis.

The final analysis will be performed when all participants complete 24 weeks visit. Immunogenicity and safety data up to 24 weeks will be included in this analysis.
Study design for additional group will be open label in which MMR recepient with or without test vaccine will be analysed for immunogenicity at 4 weeks post dose only.

Justification for the dose of Vi-DT

Previous studies conducted with other typhoid conjugate vaccines concur to use 25 µg/0.5 mL of Vi polysaccharide. Immune response in infants aged 6-8 weeks was less than older age groups when low dose (5 µg) of conjugated typhoid vaccine was used [29]. Single dose and two doses of 25 µg/0.5 mL, and two doses of 15 µg/0.5 mL were tested in a Phase 2b study in age group 2-17 years. Single dose of 25 µg/0.5 mL of Vi-TT conjugated vaccine showed excellent immune response (100% seroconversion) and was found to be safe in infants and young children aged 6-23 months. Based on these results Bharat Biotech carried out a Phase III clinical trial with single dose 25 µg/0.5 mL [23]. The Bharat Vi-TT vaccine is now licensed in India and several other countries and is WHO-prequalified also.

Similarly, in a dose ranging study, 25 µg, 12.5 µg and 5 µg of conjugate typhoid vaccine Vi-rEPA (Vi polysaccharide conjugated to exoprotein of *Pseuomonas aeruginosa*) conducted in children 2-5 years of age in Vietnam, 25 µg was found to be safe with higher immunogenicity [30]. Further 25 µg Vi-rEPA vaccine safety and immunogenicity study was conducted in infants aged 2, 4, 6 and 12 months along routine EPI vaccines and was found to be safe and immunogenic [19]. Dose of 25 µg was found to be safe and immunogenic in participants aged 2 years and above in a Phase 1 (IVI T001) conducted in the Philippines and same dose is being used in Phase II clinical trial (IVI T002) in the Philippines from 6 months to 2 years of age.

Table 1. Immunization Schedule

	**Age group**	**N**	**D0**
**Group A** (6 mo - 45 yrs) 450 participants	6 mo-<2 yrs	150	(25 µg 0.5 mL) (Vi-DT) Test Lot 1
	2- <18 yrs	150	
	18-45 yrs	150	
**Group B**	6 mo-<2 yrs	150	(25 µg 0.5 mL)

(6 mo - 45 yrs)<br>450 participants	2- <18 yrs	150	(Vi-DT)<br>Test Lot 2
	18-45 yrs	150	
**Group C**<br>(6 mo - 45 yrs)<br>450 participants	6 mo-<2 yrs	150	(25 µg 0.5 mL)<br>(Vi-DT)<br>Test Lot 3
	2- <18 yrs	150	
	18-45 yrs	150	
**Group D**<br>(6 mo - 45 yrs)<br>450 participants	6 mo-<2 yrs	150	25 µg 0.5 mL<br>(Typbar TCV®)
	2- <18 yrs	150	
	18-45 yrs	150	
			MR for age eligible<br>(9-15 months)<br>participants

Additional group:

	Age group	N	D0
Group E	9-15 months	180	(25 µg 0.5 mL) (Vi-DT) + MMR
Group F	9-15 months	180	MMR

Table 2. Schedule of Events

**Visit Number**	**V1**	**V2**	**V3**	**V4**	**V5**	**V6**
**Visit Day**	**D-7 - 0**	**D0**	**D7**	**D28**	**D84**	**D168**
**Visit Week**	**-1**	**0**	**1**	**4**	**12**	**24**
**Visit Window#**		**+1D**	**±1D**	**±3D**	**±7D**	**±7D**
**Informed Consent / assent**	**X**					
**Screening**	**X**					
**Eligibility Criteria**	**X**					
**Informed Consent / assent**	**X**					
**Inclusion & Exclusion**	**X**	**X**				

Criteria						
Medical History	X	X				
Vital Signs	X	X	X	X	X	X
Physical Examination	X	X	X	X	X	X
Enrollment & Randomization		X				
Vi-DT or Typbar TCV® Vaccination		X				
Post Vaccination 30 min F/U		X				
Solicited AE		X	X			
Unsolicited AE		X	X	X		
SAE		X	X	X	X	X
Diary Card Provided		X (DC1)	X(DC2)	X(DC3)		
Diary Card Collected			X(DC1)	X(DC2)		X(DC3)
Concomitant Medications	X	X	X	X	X*	X*
For < 2years Immunogenicity Blood Volume (approx.)		3mL		3mL		3mL
For < 2years Cumulative Blood Volume (approx.)		3 mL		6 mL		9 mL
For ≥ 2years Immunogenicity Blood Volume (approx.)		5mL		5mL		5mL
For ≥ 2years Cumulative Blood Volume(approx.)		5 mL		10 mL		15 mL

* After 4 weeks concomittant medication history will be recorded related to any SAE only

Visit window were relaxed due to COVID 19 travel restrictions (refer section 7.0)

Note: Additional group will be followed-up for 4 week post IP dose, hence only DC1 & DC2 will apply for this group.

5.3 MEASURES TO MINIMIZE BIAS

5.3.1 RANDOMIZATION/MASKING PROCEDURES

The randomization list will be generated by an independent statistician who is not directly involved in the study conduct at IVI. The randomization will be stratified by three age strata and four study sites. Eligible participants will be assigned to receive single dose of one of the three lots of Vi-DT test vaccine or comparator vaccine in a 1:1:1:1 ratio in each strata. The randomization list will contain sequential numbers unique to each participant and treatment group (A to D) and the block randomization process with random block size of 4 and 8 will be employed to ensure an effective balance between the interventions.

For additional group, eligible participants will be assigned to receive single dose of Vi-DT test+MMR vaccine or MMR alone in a 1:1 ratio in 9-15 months age stratum. The randomization list will contain sequential numbers unique to each participant and treatment group (E & F) and the block randomization process with random block size of 4 and 8 will be employed to ensure an effective balance between the interventions.

Two types of randomization list, one with randomization number only, second with randomization number and vaccine allocation will be prepared. Participants in the study will be randomized into four treatment groups within each age strata: 6 to less than 24 months, 2 to less than 18 years, and 18 to 45 years. Randomization list “without the treatment allocation” with only numbers will be given to the blinded trial staff, for enrolling the trial participants and assigning them the randomization number. The randomization list “with the vaccine allocation” will be given to the unblinded vaccine administrator (nurse/pharmacist). If feasible, a web based randomization system will be provided to each site.

Upon enrollment, in order to receive the study vaccine, participants will be sent to the vaccine administrator with their randomization number. The unblinded study nurse/pharmacist located in a different room will administer vaccine(s) to the participant according to the randomization list. The randomization number of the participant receiving the study vaccine will be written on the empty vaccine vial and on the vaccine accountability log for record and reconciliation.

Trial staff other than the unblinded study staff will remain blinded to vaccine administration. The unblinded study nurse will not be involved in the evaluation of vaccine safety and will not discuss with the investigator and clinical staff about vaccines administered.

5.3.2 UNBLINDING OF PARTICIPANTS

Unblinding will be considered on a case-by-case basis and only in the case of a life-threatening condition or serious medical emergency when the vaccine allocation is judged relevant for the safety of the participants. The site investigator will be provided with an option to receive the randomization individual information with treatment allocation of Test or Comparator vaccine. If the site Investigator considers necessary to unblind a participant for safety reasons, he/she can break the blind code, confirm the relevant vaccine from the randomization individual treatment allocation information provided, and take actions accordingly. If an accidental unblinding occurs, the unblinding should be documented and recorded. The investigator will inform the sponsor by email or fax.

Study participants will be unblinded after completion of final analysis. The database will be locked for the final analysis after safety and immunogenicity is cleaned at 24 weeks. For additional group the database will be locked for the final analysis after safety and immunogenicity is cleaned at 4 weeks or along with 1800 participants database lock time.

6 STUDY AGENT

6.1 STUDY AGENT(S) AND CONTROL DESCRIPTION

6.1.1 ACQUISITION

Test vaccine lots manufactured by SK bioscience in the Republic of Korea will be shipped to study sites in Nepal. Comparator vaccine, Typbar TCV® manufacture by Bharat Biotech, India will be purchased through local distributor. Site will procure locally licensed MR vaccine for the eligible participants. Other EPI vaccines recommended for the age of the child will also be administered at the study site. The pharmacist will receive the study agents and will be responsible for accounting, storage, handling and administration of the vaccines.

6.1.2 FORMULATION, APPEARANCE, PACKAGING, AND LABELING

Test Vaccine

1) Code name: NBP618
2) Manufacturer: SK bioscience Co., Ltd.
3) Ingredient: Purified Vi-polysaccharide conjugated to diphtheria toxoid

4) Appearance: Clear, colorless liquid
5) Dose: 25 µg of Vi polysaccharide/0.5 mL, presented in 3 mL multi-dose glass vial

Table 4. Test Vaccine Content

Function	Component	Reference	Quantity/0.5 mL
Active ingredient	Vi polysaccharide conjugated to diphtheria toxoid	In-house	25 µg
Stabilizer	Di Sodium hydrogen phosphate	EP	0.620 mg
Stabilizer	Sodium Dihydrogen Phosphate dihydrate	EP	0.152 mg
Stabilizer	Sodium chloride	EP	4.25 mg
Diluent	Water for injection	EP	q.s.
Preservative	2-phenoxy ethanol	EP	5.0 mg

6) Packaging: test vaccine will be manufactured and packaged by SK bioscience and supplied to the study pharmacy nurse at the clinical site. The Test Vaccine will be labelled by SK bioscience as Test Vaccine (Vi-DT).
7) Labeling: will be done as per health science authority guidelines section 3.3[31.]Investigational Medicinal Product Dossier, the template of vial and carton label are as follows:
8) Storage condition: hermetic container (3 mL multi-dose glass vial), protected from light, +2- 8°C. DO NOT FREEZE
9) Retest period: 18 months from date of manufacturing

Test Vaccine vial label:

“For Clinical Trial Use Only”
Protocol No. : IVI T003
Lot No. : OOOOOO, OOOOOO or OOOOOO
Randomization No.:OO_OOO
Drug Code: OOOO
Retest Date : Up to 18 months from the date of manufacture (Mfd. Date: YYYY.MM.DD)
Storage Condition: hermetic container, protected from light, +2oC to 8oC without freezing
Manufacturer : SK bioscience Co., Ltd.,

Box Label:

“For Clinical Trial Use Only”<br>Protocol No. : IVI T003<br>Lot No. : OOOOOO, OOOOOO, or OOOOOO<br>Retest Date: Up to 18 months from the date of manufacture (Mfd. Date: YYYY.MM.DD)<br>Storage Condition: hermetic container, protected from light, +2oC to 8oC without freezing<br>Manufacturer and Address: SK bioscience Co., Ltd., 150, Saneopdanji-gil, Pungsan-eup, Andong-si, Gyeongsangbuk-do, Republic of Korea<br>Dosage and administration: 0.5mL/dose with intramuscular injection in the left anterolateral thigh or left arm deltoid region in participants below 2 years of age, left arm deltoid region in age group 2-45 years

Comparator vaccine

Typbar TCV ®

1) Product Name: Typbar TCV®
2) Manufacturer: Bharat Biotech
3) Ingredient: Purified Vi capsular polysaccharide of Salmonella Ty2 conjugated to tetanus toxoid protein
4) Appearance: Clear, colorless appearance
5) Dose: 0.5mL, intramuscular injection
6) Packaging: Liquid, single dose vial
7) Labeling: Keep original label as registered in the Nepal
8) Storage condition: protected from light, stored at +2°C to 8°C

6.1.3 PREPARATION

The preparation and administration of the vaccines to participants enrolled into the study will only be done by the unblinded study nurse according to the procedures stipulated in this study protocol. The unblinded study nurse responsible for vaccine administration is qualified to perform this task and same will be documented by site investigator.

The licensed vaccines will be prepared before use according to the package insert. For further details please refer to the MOP.

6.1.4 DOSING AND ROUTE OF ADMINISTRATION

Vi-DT manufactured by SK bioscience, Republic of Korea, is formulated to contain purified Vi-polysaccharide 25 μg in 0.5 mL per dose. 0.5 mL by intramuscular injection in the left anterolateral thigh or left arm deltoid region in participants below 2 years of age, left arm deltoid region in age group 2-45 years.

In case the left thigh cannot be used for vaccination due to infection, eczema or injury, the right thigh will be used for vaccination instead. If the left deltoid cannot be used for vaccine administration, the right deltoid will be used instead.

MR vaccine will be given concomitantly with Vi-DT or Typbar TCV® for eligible participants aged 9 to 15 months subcutaneously in thigh or deltoid region different from the site of test/comparator vaccine injection in all study groups. If a participant in age group 9 to 15 months presents to the study site after receiving MR vaccine elsewhere, the participant will not be enrolled in the study in MR group.

6.1.5 DOSE ADJUSTMENTS/MODIFICATIONS/DELAYS

There is no plan of Vi-DT dose adjustment and modification during the course of study.

6.1.6 TRACKING OF DOSE

All study agents and MR and other EPI vaccines administered will be registered in the vaccine administration log and eCRF by the Study Pharmacist/Nurse/Coordinator. The unblinded study monitor will ensure timeline adherence for administration of study agent.

6.2 STUDY AGENT ACCOUNTABILITY PROCEDURES

Used and unused Vi-DT will be disposed at site or returned as per the guidance from the manufacturer. All expired vaccines will be disposed as per the site SOP for disposal (See details in the MOP).

6.3 STANDARD OF CARE

- Appropriate medical care and treatment to participants in need during the trial will be provided as per standard of care practice in the Nepal.

- The site investigator will conduct appropriate medical investigations deemed necessary to evaluate any medical conditions that might arise during the course of the study and ensure that participants receive appropriate care and are referred to appropriate health services as needed.

6.4 CONCOMITANT MEDICATIONS, TREATMENTS, AND PROCEDURES

A prescription medication is defined as a medication that can be prescribed only by an authorized/licensed physician. Medications to be reported in the CRF are concomitant prescription medications, over-the-counter medications and supplements.

7 STUDY POPULATION

7.1 STRATEGIES FOR RECRUITMENT AND RETENTION

The targeted sample size is 1800, 450 each of the four groups. Target sample size for additional group is 360 participants aged 9-15 months. Study staffs will approach participants/parents/LAR between Days -7 to 0 at the health facility. The enrollment venue is the clinical trial sites in Nepal. Participants/Parents/LAR with aged 6 months to 45 years visiting health centers for regular immunizations or medical check-up who may be interested in participating in the study, will be asked to go to clinical trial site during the recruitment period. Beside that healthy volunteers from the community will be mobilized to trial sites for recruitment with the help of field health workers. Participants in the age group 18-45 years will be asked for consent while assents in addition to parents/LAR consent will be required for participants in the age group 7 - <18 years. Parents/LAR of participants in age group 6 months to 7 years will be asked for consent. After vaccination, field health worker/designee will contact participant/parents/LAR every day till Day 7 by physical visit or by phone call. The study will require long-term participation therefore, telephone follow-up reminder calls will be done very frequently as per discretion of study staff until 24 weeks for all study groups.

7.2 CONSENT AND ASSENT PROCEDURES AND DOCUMENTATION

Informed consent is a process that is initiated prior to the individual's agreeing to participate in the study and continues throughout the individual's study participation. Assent is a process that

is initiated prior to the individual's aged 7 -<18 years, agreeing to participate in the study and continues throughout the individual's study participation. Assent will be taken from participant (7 -<18 year age) in addition to the consent from parents/LAR. Extensive discussion of risks and possible benefits of participation will be provided to the participants/parents/LAR. Consent/Assent forms will be EC/IRB-approved prior to their use and the participant/parents/LAR will be asked to read and review the document.

The investigator or designated study team member will explain the study to the participant/parents/LAR and answer any questions that may arise. All participants will receive a verbal explanation in terms suited to their comprehension of the purposes, procedures, and potential risks of the study and of their rights as research participants. The participant/parents/LAR will have the opportunity to carefully review the written consent/assent form and ask questions prior to signing. Participant/parents/LAR may wish to discuss the study with family or friends before making any decision as to whether or not to participate in the study and come back later to inform the site Investigator or designee of his/her decision. For those individuals who express interest in continuing with the consent/assent process, the site investigator or designee will review the consent/assent form privately in detail with the participant/parents/LAR and answer any questions.

Before signing the consent/assent, participants/parents/LAR will be asked to undergo an informed consent process validation to ensure that they fully understand the purpose of the study, procedures, potential risks and their rights in this study.

The participants/parents/LAR will sign the informed consent/assent documents prior to any procedures being done specifically for the study. The participants/parents/LAR may withdraw consent/assent at any time throughout the course of the trial. A copy of the informed consent/assent document will be given to the participants/parents/LAR for their records. The rights,safety and welfare of the participants will be protected by emphasizing that the quality of their medical care will not be adversely affected even if they decline to participate in this study.

Although ICF will be amended based on this version of protocol, participants recruited in the main study group will not be reconsented on the ICF intended for additional group.

7.3 COMPENSATION FOR PARTICIPATION

Participants will not be compensated for participation in the study. However a prorated and reasonable reimbursement to the participants/parents/LAR for travel expenses, meals, and time

lost from work / time spent at the study site will be given as approved by the local IRB.

7.4 PARTICIPANT INCLUSION CRITERIA

In order to be eligible to participate in this study, any individual must meet the following criteria:

- Healthy participants 6 months to 45 years of age at enrollment
- Healthy participants 9 to 15 months of age at enrollment (additional group only)
- Participants/parents/LAR who have voluntarily given written informed consent/assent
- Participants/parents/LAR willing to follow the study procedures and available for the entire duration of the study

7.5 PARTICIPANT EXCLUSION CRITERIA

An individual who meets any of the following criteria will be excluded from participation in this study:

- Child with a congenital abnormality
- Subject concomitantly enrolled or scheduled to be enrolled in another trial
- Known history of immune function disorders including immunodeficiency diseases (Known HIV infection or other immune function disorders)
- Chronic use of systemic steroids (>2 mg/kg/day or >20 mg/day prednisone equivalent for periods exceeding 10 days), cytotoxic or other immunosuppressive drugs
- Receipt of blood or blood-derived products in the past 3 months
- Subject with a previously ascertained or suspected disease caused by *S*. Typhi
- Subject who have had household contact with/and or intimate exposure to an individual with laboratory-confirmed *S*. Typhi.
- Individual who has previously received a typhoid vaccine.
- Subject who has received or is expected to receive other vaccines from 1 month prior to IP vaccination to Visit 4 (approx.1 month post IP) except PCV booster as per EPI schedule or any vaccine during National Immunization catch-up campaign of Nepal.
- Known history or allergy to vaccines or other medications
- History of uncontrolled coagulopathy or blood disorders
- Any abnormality or chronic disease which in the opinion of the investigator might be detrimental for the safety of the subject and interfere with the assessment of the study objectives.
- Any female participant who is lactating, pregnant or planning for pregnancy during the course of study period.
- Participants/Parents/LAR planning to move from the study area before the end of study period

- As per Investigator's medical judgement individuals could be excluded from the study inspite of meeting all inclusion/exclusion criteria mentioned above

Temporary Contraindication

- Acute illness, in particular infectious disease or fever (axillary temperature ≥37.5°C), within three days prior to enrollment and vaccination.

These individual could be rescreened upon resolution of the above said conditions

*Urine pregnancy test (UPT) will be performed in all married females prior to injection

7.6 STUDY PROCEDURES

7.6.1 SCREENING

Informed Consent / Assent Process

The informed consent/assent process will be conducted as described above. The date of signature of the informed consent/assent will be entered on the eCRF.

The screening process will take place during the week prior to enrollment (Days -7 to 0). After written consent/assent are obtained, screening procedures will be performed as described in the MOP. The study staff will motivate the screened eligible participants/parents/LAR to visit trial site on Day 0 for vaccination. A participant will be enrolled if he/she meets all inclusion and exclusion criteria and his/her health status is deemed acceptable as determined by medical history, physical examination, and medical judgement of the site investigator. If participants/parents/LAR agrees, screening and enrollement could be done on the same day. A study ID card will be provided to each enrolled participant.

Details of visit procedures during screening are as follows:

Visit 1 - Screening visit (Days -7 to 0)

1) Explain study objectives and procedures, risk and benefits to the participants/parents/LAR
2) Perform informed consent process validation

3) Obtain written informed consent/assent from the participant/parents/LAR. Written Informed consent/assent will be obtained prior to performance of any study-specific screening or evaluations.
4) Perform screening procedures within 0 to 7 days prior to first vaccination.
5) Collect demographics, medical history, vital signs and check for inclusion and exclusion criteria
6) Only Married females volunteers will be screened for pregnancy with UPT.
7) Schedule participant for enrollment and vaccination visit (V2) at the study center within 7 days after the screening visit. If an eligible participant does not come back for V2 within the 7 days after the screening visit, screening procedures must be repeated.
8) Screening and enrollement could be done on the same day, which will waive off the need for visit 2.
9) Participants who do not meet the criteria for participation in this trial because of fever or acute illness, may be rescreened when these conditions have resolved. Rescreened participants will be assigned the same screening number as for the initial screening.

7.6.2 ENROLLMENT

Informed consent form will be verified by study staff and inclusion and exclusion criteria will be reviewed.
Participant will be randomized to test/comparator groups into three age strata of age 6 months to less than 2 years, 2 to less than 18 years, and 18 to 45 years. Participants will be attributed a enrollment number. Before randomization, medication history will be obtained, vital signs and results from physical examination, growth and development evaluations will be recorded. Blood for the immunogenicity will be obtained prior IP administration.

Details of visit procedures during enrollment are described below and in the MOP:

Visit 2 - Enrollment and First Vaccination (Day 0)

1) Collect medical history, perform clinical examination and check concomitant therapies and record in the eCRF.
2) Confirm eligibility of participant
3) Perform enrollment, randomization and attribute enrollment number to participant.

4) Perform first vaccination as per instructions of the Manual of Procedures.
5) Monitor participant for 30 min following vaccination as follows:
    - Local examination of injection site
    - Clinical examination including vital signs, general physical examination before leaving the study center after 30 min post vaccination
    - Record solicited and unsolicited adverse reactions (if any occurring)
6) Schedule next visit to the study center and remind participant/parent/LAR to bring the diary card at the next visit
7) Participant/parent/LAR will be instructed to evaluate local and systemic reactions at home for 7 days post immunization (from Day 0 to Day 6 post vaccination day). Diary card 1 will be issued to record adverse events and participant/parent/LAR will be instructed how to fill in the diary card 1. A thermometer will be given along with diary card 1 to record fever. Next clinic visit after 7 days will be scheduled. Participant/parent/LAR will also be instructed to contact investigator/ study staff if needed.

7.6.3 FOLLOW-UP PROCEDURES AND VISITS

Days 0-6

The study staff will contact the participant/parent/LAR by telephone or physical visit to remind them and provide assistance to record local and systemic reactions with the use of the diary card 1 and to remind the date of the next visit day .

Visit 3 – Safety follow-up visit (Day 7 ± 1 day)

1) Record solicited and unsolicited adverse events since last visit in eCRF
2) Verify that the participant/parent/LAR fills in the diary card 1 correctly
3) Perform clinical examination (including vital signs and general physical exam) and record in eCRF
4) Hand over the diary card 2 to be filled till day 28 post vaccination
5) Schedule next visit V4 and remind participant parent or legal guardian to contact investigator/ study staff if needed.

Note: For additional group, visit 3 could be physical or telephonic visit

Visit 4 – Follow-up visit (Week 4, Day 28 ± 3 days)

1) Check diary card 2 and confirm with participant/parent/LAR before recording observations in the eCRF
2) Record any unsolicited AE, SAE and concomitant medications in eCRF
3) Perform clinical examination including vital signs, general physical examination
4) Perform blood draw for assessment of immune responses post first vaccination

Schedule next visit V5 and handover dairy card 3 to participant/parent/LAR to capture any SAEs till 24 weeks (168 days) post vaccination.

Visit 5 – Follow-up visit (Week 12, Day 84 ± 7 days)

1) Examine diary card 3 and record any SAE and concomitant medications in eCRF
2) Perform clinical examination including vital signs, general physical examination
3) Schedule next visit to the study center

Visit 6 - Follow-up visit (Week 24, Day 168 ± 7 days)

This is the final visit of the study.

1) Collect diary card 3 and check with participant/parent/LAR before recording observations in the eCRF
2) Record any SAE and concomitant medications in eCRF
3) Perform clinical examination including vital signs, general physical examination
4) Perform blood draw for assessment of immune responses
5) Ensure source documents are duly completed before the participant/parent/LAR leave the study center
6) Participant will be continued to be monitored after study ends for unresolved AE.

Note: Due to COVID 19 Pandemic, relaxation for the visit 5 & 6 were given in the form of replacement of Physical visits by telephonic follow-up and visit window relaxation (upto 28 days) as per Note to File submitted to NRA/IRBs dated 23MAR2020 & 13MAY2020.

In case Participant/parent/LAR is illiterate, field health worker will help them in filling up the diary cards.

Beside scheduled visits participants will be instructed to visit site anytime during the study

period due to any safety concerns. For further details please refer to the MOP.

.

If any participant becomes pregnant during the conduct of the study, then the participant would be followed up till delivery and informations will be captured in Pregnancy form. Detailed information regarding Pregnancy will be mentioned in the safety management plan.

7.7 PARTICIPANT WITHDRAWAL OR TERMINATION

7.7.1 REASONS FOR WITHDRAWAL OR TERMINATION

Participant/parent/LAR are free to withdraw from participation in the study at any time upon request, without justification and without prejudice. The Site Investigator may also decide to discontinue participation of a participant from study interventions in the following cases:

1) An acute reaction (allergy, hypersensitivity reaction, etc.) to the investigational product.
2) Occurrence of an illness or serious adverse event or adverse event that in the judgment of the investigator may be detrimental for the participant's safety.
3) A study participant/parent/LAR withdrawal of informed consent.
4) A study participant's medical condition or use of medication that in the judgment of the investigator may compromise the participant's safety and/or the scientific integrity of the study.
5) Violation of the inclusion/exclusion criteria by the participant.
6) A study participant's no-show for a scheduled visit without notices, unable to contact/trace, and lost to follow-up.
7) Any other reason of study discontinuation as per the judgment of the site Investigator

7.7.2 HANDLING OF PARTICIPANT DISCONTINUATION OR TERMINATION

Discontinuation from study intervention does not mean discontinuation from the study, and remaining study procedures will be completed as indicated in the study protocol. If a clinically significant finding is identified after enrollment, the PI will determine if any change in participant management is needed. Any new clinically relevant finding will be reported as an adverse event.

Study team will encourage withdrawn or terminated participant to continue in the study for safety follow-up. If the participant/parent/LAR declines, this will end the participant's/parent's/LAR's interaction with the study team for this protocol. The study team will engage in no further communication with the volunteer except as directed by an IRB with regard to participant safety information. Protocol-specified safety follow-up procedures will be discussed with the participant/parent/LAR to capture AEs, SAEs. The reason for participant discontinuation or withdrawal from the study will be recorded on the study follow-up eCRF. Only data and samples already collected will be analyzed according to protocol. The study team will not utilize samples or data from this volunteer for any future use and will discard residual samples when the study is completed. Counseling about any issue will be provided if participant/parent/LAR decides to discontinue participation in the study. Medical advice will also be provided in the best interest of the participant.

Participants who receive the study intervention and subsequently withdraw, or are withdrawn or discontinued from the study will not be replaced.

In the event of early termination of a participant:

1) Date and Reason for early termination of the participant will be recorded in the eCRF.
2) Any unsolicited AE, SAE and concomitant medications up to that points will be recorded in eCRF.
3) Clinical examination including vital signs, general physical examination will be performed if participant/parent/LAR is willing.

7.8 LOST TO FOLLOW-UP

A participant will be considered lost to follow-up if he or she fails to return for any of scheduled visits and remains unreachable to study site staff.

The following actions will be taken if a participant fails to return to the clinic for a required study visit:

- The site staff will attempt to contact the participant/parent/LAR and counsel on the importance of maintaining the assigned visit schedule and ascertain if the participant/parent/LAR wishes to and/or should continue in the study.

- Before a participant is deemed lost to follow-up, the investigator or designee will make every effort to regain contact with the participant/parent/LAR (will make at least 3 telephone calls). These contact attempts should be documented in the participant's medical record or study file.

Should the participant continue to be unreachable, he/she will be considered to have withdrawn from the study with a primary reason of lost to follow-up.

7.9 PROTOCOL DEVIATIONS

A protocol deviation is any non-compliance with the clinical trial protocol or GCP by the participant, the investigator, or the study site staff and Sponsor. It is the responsibility of the site investigator to use continuous vigilance to identify and report all major protocol deviations to site IRB and sponsor. Minor protocol deviations could be reported to IRBs along with periodic study report submission as per local IRBs requirements. The site investigator is responsible for knowing and adhering to the site IRB requirements. The IVI Study Medical Monitor will report all protocol deviations to IVI IRB.

Major deviations are defined as those jeopardize the safety or rights of the participant or the scientific integrity of the study which may be applicable to cases listed below.

- Violation of inclusion and exclusion criteria
- Vaccination with wrong vaccine as defined in the protocol
- Visit outside window for the immunogenicity assessment after discussion with the study medical monitor
- Missed samples for immunogenicity

Major protocol deviations thought to affect the scientific integrity of the study will be reported and discussed with investigator, monitor, sponsor, and statistician for their exclusion from the per protocol analysis. For minor protocol deviations considered not to affect the scientific integrity of the study, the extent of deviation or delay as well as reason will be accurately documented.

7.10 PROTOCOL AMENDMENTS

Any amendment of the approved protocol shall be submitted to all IRBs (IVI and site IRBs) and to the National Regulatory Authority for information only before use.

7.11 PREMATURE TERMINATION OR SUSPENSION OF STUDY

This study may be temporarily suspended or prematurely terminated if there is sufficient reasonable cause as per PI recommendation after consultation with sponsor. NHRC, site IRBs and DDA may also require termination of the study. Written notification, documenting the reason for study suspension or termination, will be provided by the suspending or terminating party to investigator, the sponsor, the regulatory authorities, and IRBs.

Circumstances that may warrant termination or suspension are:

- Determination of unexpected, significant, or unacceptable risk to participant as recommended by the PI
- Poor protocol compliance

Study may resume once concerns about safety, protocol compliance, data quality are addressed and satisfy the sponsor, NHRC, Site IRBs and/or DDA.

7.12 END OF STUDY

A participant is considered to have completed the study if he or she has completed all phases of the study including the last visit or the last scheduled procedure shown in the Schedule of Events (SOE).

8 LABORATORY PROCEDURES/EVALUATIONS

8.1 SPECIMEN PROCESSING, HANDLING, AND STORAGE

Venous blood will be collected from participants for immunogenicity assessments. Whole blood will be centrifuged, and sera will be aliquoted and stored at below -20°C until shipment to IVI and use. Pre-print study labels provided in advance will be attached on each of the serum aliquots.

8.2 SPECIMEN SHIPMENT

Aliquoted blood samples for immunogenicity assessment will be shipped from clinical trial site to the International Vaccine Institute, Seoul, Republic of Korea, where they will be stored at -70°C for analysis and storage. as per informed consent for Research of Human Derivatives.

8.3 ASSESSMENT OF IMMUNOGENICITY

Anti-Vi IgG ELISA

The assay is used to measure anti-Vi specific antibodies of the IgG in human sera. Poly-L-lysine is pre-coated and purified Vi antigen at a concentration of 2 µg/mL is absorbed onto 96-well microtiter plates. Non-specific binding sites are blocked with BSA in PBS, diluted human sera is then added to the first wells in the plate then serially diluted across the plate. Antibodies specific to Vi will bind to the Vi coated to the plate. The bound IgG is detected using alkaline phosphatase labelled goat anti-human IgG. Addition of 4-nitrophenyl phosphate substrate causes a color change proportional to the amount of human anti-Vi IgG antibody present in the serum. Optical Densities of the wells are measured at 405 nm. The level of the specific anti-Vi IgG in ELISA units for each serum sample is determined by comparison to a reference serum.

Measles, Mumps and Rubella Antibody Assays

Measles, Mumps and Rubella antibody titers will be measured by ELISA using a commercial kit.

9 ASSESSMENT OF SAFETY

9.1 SAFETY ASSESSMENT

The following procedures will be performed to monitor safety as listed in the SOE:

- ***Demographic and medical history*** *(DOB, age, gender, baseline medical history of participants)*
- ***Physical examination*** *(height/length and weight, organ systems, growth and development and motor assessments for age eligible participants)*
- ***Vital signs*** *(temperature, pulse, respirations)*
- **Diary cards** will be used for participant/parents/LAR reported outcomes.

At each study visit, the investigator will inquire about the occurrence of AE/SAEs since the last visit.

9.1.1 DEFINITION OF ADVERSE EVENTS (AE)

Adverse events (AE) are defined as any untoward medical occurrence which follows immunization and which does not necessarily have a causal relationship with the administration of the vaccine. An AE may be any unfavorable or unintended sign, symptom, abnormal laboratory finding or disease.

Solicited AEs are predetermined events, identified in the Investigator's Brochure, which may reflect safety concerns related to the investigational product. AEs that will be solicited by the participant/ parents/LAR and recorded in the diary card and reviewed by a blinded observer during the 7 days after each dose for this study include:

    - Local reactions at the site of injection: Pain/tenderness, erythema/redness, induration/swelling, pruritus pruritus associated with injection

- Systemic reactions (adapted by age group): Fever, headache, fatigue, myalgia, lethargy, irritability, nausea, vomiting, arthralgia, diarrhea, drowsiness, loss of appetite, chills, rash, nasopharyngitis and persistent crying
- Rash and nasopharyngitis are described as possible MR vaccine-specific adverse events and will be collected following the vaccine administration at Day 0 for children aged 9 to less than 15 months.

Unsolicited AEs are all other adverse events (those that do not fall under the categories of solicited Adverse Reactions) that are identified by site staff, the site investigator and the Medical Research Monitors. These unsolicited AEs will be documented in the participant's study records and entered in the study eCRFs.

Results will be expressed as frequency of the AEs and individual descriptions will be tabulated according to MedDRA organ class system.

9.1.2 DEFINITION OF SERIOUS ADVERSE EVENTS (SAE)

An AE or suspected adverse reaction is considered "serious" if, in the view of either the investigator or sponsor, it results in any of the following outcomes:

- Death
- Life-threatening event
- In-patient hospitalization > 24 hours or prolongation of existing hospitalization
- Persistent or significant incapacity or substantial disruption of the ability to conduct normal life functions
- Congenital anomaly/birth defect
- Important medical events that may not result in death, be life-threatening, or require hospitalization may be considered serious when, based upon appropriate medical judgment, they may jeopardize the participant and may require medical or surgical intervention to prevent one of the outcomes listed in this definition

9.1.3 SUSPECTED UNEXPECTED SERIOUS ADVERSE REACTIONS (SUSAR)

It is defined as a serious adverse reaction whose nature or severity is not consistent with the applicable product information, VI-DT Investigator's Brochure or the summary of product characteristics of an authorized product.

9.2 CLASSIFICATION OF AN ADVERSE EVENT

9.2.1 SEVERITY OF EVENT

All solicited AEs in the study will be graded for their severity and recorded in the eCRF as described in below table. All other AEs will be assessed by the study clinician using the National Institute of Allergy and Infectious Diseases (NIAID) Division of AIDS Table for grading the severity of Adult and Pediatric Adverse events [32]. And Ministry of Food and Drug Safety (MFDS) of guideline for grading the severity of Vaccine clinical trial Adverse events [33]

Table 5. Solicited systemic adverse reaction severity grading

Systemic (General)	Mild (Grade1)	Moderate (Grade2)	Severe (Grade3)	Potentially Life Threatening (Grade 4)
Fever*	38.0 – 38.5°C	38.6 – 39.2°C	39.3 – 39.9°C	> 40°C
Irritability	Require minimal or no treatment	Results in low level of inconvenience or concern	Interrupt daily activity and require drug therapy	ER visit or hospitalization
Nausea**	No interference with routine activity	Some interference with routine activity	Significant; prevents routine daily activity	ER visit or hospitalization
Vomiting**	No interference with routine activity	Some interference with routine activity	Significant; prevents routine daily activity	ER visit or hospitalization
Diarrhea*	No Interference with routine activity 1-2 episodes/24	Some Interference with routine activity > 2 episodes/24	Prevents daily activity requires outpatient IV hydration	ER visit or hospitalization for hypotensive shock
Drowsiness	No interference with routine activity	Some interference with routine activity	Significant; prevents daily routine activity	ER visit or hospitalization
Lethargy	No interference with routine activity	Some interference with routine activity	Significant; prevents daily routine activity	ER visit or hospitalization
Headache	No interference with routine activity	Some interference with routine activity	Significant; prevents daily routine activity	ER visit or hospitalization
Fatigue	No interference with routine activity	Some interference with routine activity	Significant; prevents daily routine activity	ER visit or hospitalization
Mayalgia	No interference with routine activity	Some interference with routine activity	Significant; prevents daily routine activity	ER visit or hospitalization
Loss of appetite	Require minimal or no treatment	Results in low level of inconvenience or	Require drug therapy	ER visit or hospitalization

		concern		
Persistent crying	Require minimal or no treatment	Results in low level of inconvenience or concern	Interrupt daily activity and require drug therapy	ER visit or hospitalization
Arthralgia	No interference with routine activity	Some interference with routine activity	Significant; prevents daily routine activity	ER visit or hospitalization
Chills	No interference with routine activity	Some interference with routine activity	Significant; prevents daily routine activity	ER visit or hospitalization
Rash	Localized Rash	Diffuse rash OR Target lesions	Diffuse rash and vesicles or limited number of bullae or superficial ulceration of mucus membranes limited to one side	Extensive or generalized bullous lesion OR ulceration of mucus membrane involving two or more distinct mucosal sites OR Stevens Johnson syndrome
Nasopharyngitis	Require minimal or no treatment	Results in low level of inconvenience or concern	Interrupt daily activity and require drug therapy	ER visit or hospitalization

* Axillary temperature will be recorded.

** Adapted gradings from DIAIDS guideline

Table 6. Solicited local adverse reaction severity grading

Local Reaction to Injectable Product	Mild (Grade 1)	Moderate (Grade 2)	Severe (Grade 3)	Potentially Life Threatening (Grade 4)
Pain /Tenderness	Does not interfere with routine activity	Interferes with routine activity or repeated use of non-narcotic pain reliever	Prevents routine daily activity or repeated use of narcotic pain reliever	Emergency room (ER) visit or hospitalization
Erythema/Redness (Adolescents & adults, age ≥12yrs)	Affected area 25 - < 50mm in diameter	Affected area 50-<100mm in diameter	Affected area ≥ 100 mm in diameter	Necrosis or exfoliative dermatitis
Erythema/Redness (Children, age 2<12 yrs)	< 25 mm in diameter	25 – 50 mm in diameter	≥ 50 mm in diameter	Necrosis or exfoliative dermatitis
Erythema/Redness (Children, age < 2 yrs)	< 10 mm in diameter	10 < 25 mm in diameter	< 50 mm in diameter	≥ 50 mm in diameter
Swelling/Induration (Adolescents & adults, age ≥12yrs)	Affected area 25 - < 50mm in diameter	Affected area 50-<100mm in diameter	Affected area ≥ 100 mm in diameter	Necrosis or exfoliative dermatitis
Swelling/Induration (Children, age 2<12 yrs)	< 25 mm in diameter	25 – 50 mm in diameter	≥ 50 mm in diameter	Necrosis or exfoliative dermatitis
Swelling/Induration (Children, age < 2 yrs)	< 10 mm in diameter	10 < 25 mm in diameter	< 50 mm in diameter	≥ 50 mm in diameter

Pruritis associated with injection	Itching localized to injection site AND Relieved spontaneously or with < 48 hours treatment	Itching beyond the injection site but not generalized OR Itching localized to injection site requiring ≥ 48 hours treatment	Generalized itching causing inability to perform usual social & functional activities	Emergency room (ER) visit / hospitalization

- All local AE for MR/MMR will be graded for their severity as per solicited local adverse reaction severity grading.

- All unsolicited adverse events observed by investigator and/or reported by participants/parent/LAR after discussing with the investigator will be recorded in the eCRFs with their severity grading and relatedness to the study vaccine.

- All SAEs irrespective of their causal association will also be graded for their severity.

9.2.2 RELATIONSHIP TO INVESTIGATIONAL PRODUCT

For all collected AEs, the clinician who examines and evaluates the participant will determine the relationship of each AE with the investigational product based on plausible biologic mechanism, temporal relationship of occurrence after administration of the investigational product, identification of possible alternative etiologies including underlying disease, concurrent illness or concomitant medication, and his/her clinical judgment. The relationship of vaccination to adverse event (AE) will be determined based on the definitions below.

- **Definitely Related** – There is clear evidence to suggest a causal relationship, and other possible contributing factors can be ruled out. The clinical event, including an abnormal laboratory test result, occurs in a plausible time relationship to vaccine administration and cannot be explained by concurrent disease or other drugs or chemicals. The response to withdrawal of the Vaccine (dechallenge) should be clinically plausible.
- **Probably Related** – There is evidence to suggest a causal relationship, and the influence of other factors is unlikely. The clinical event, including an abnormal laboratory test result, occurs within a reasonable time after administration of vaccine, is unlikely to be attributed to concurrent disease or other drugs or chemicals, and follows a clinically reasonable response on withdrawal (dechallenge).

- **Possibly Related** – There is some evidence to suggest a causal relationship (e.g., the event occurred within a reasonable time after administration of vaccine). However, other factors may have contributed to the event (e.g., the participant clinical condition, other concomitant events). Although an AE may rate only as "possibly related" soon after discovery, it can be flagged as requiring more information and later be upgraded to "probably related" or "definitely related," as appropriate.
- **Unlikely to be related** – A clinical event, including an abnormal laboratory test result, whose temporal relationship to vaccine administration makes a causal relationship improbable (e.g., the event did not occur within a reasonable time after administration of vaccine) and in which other drugs or chemicals or underlying disease provides plausible explanations (e.g., the participant clinical condition, other concomitant treatments).
- **Not Related** – The AE is completely independent of vaccine administration, and/or evidence exists that the event is definitely related to another etiology. There must be an alternative, definitive etiology documented by the clinician.

9.2.3 EXPECTEDNESS

The Study Medical Monitor in consultation with site PI will be responsible for determining whether an AE is expected or unexpected. An Adverse Reaction will be considered unexpected if the nature, severity, or frequency of the event is not consistent with the risk information previously described for the study agent.

9.3 TIME PERIOD AND FREQUENCY FOR EVENT ASSESSMENT AND FOLLOW-UP

All participants will be observed for immediate local and systemic reactions for 30 minutes after each vaccination. For 7 consecutive days (Days 0-6) after each dose of study vaccine, the participant/parent/LAR will be asked to record solicited local and systemic symptoms in the diary card. The study staff will remind participant/parent/LAR of the importance of properly filling the diary cards and to return the cards at the next scheduled study visit. If they did not fill up or lost their card, the parent or legal guardian will be interviewed for recall of symptoms with trained study staff during clinic visit on Day 7 after vaccination.

The occurrence of an adverse event (AE) will come to the attention of study personnel during study visits and interviews of a study participant presenting for medical care, or upon review by a study monitor.

All AEs including local and systemic reactions not meeting the criteria for SAEs will be captured on the appropriate case report form (eCRF). Information to be collected includes event description, time of onset, symptoms and physical examination findings, clinician's assessment of severity, relationship to study product (assessed by the SI), medications given and time of resolution/stabilization of the event. All AEs occurring while on study will be documented appropriately regardless of relationship.

Changes in the severity of an AE will be documented to allow an assessment of the duration of the event at each level of severity to be performed. AEs characterized as intermittent require documentation of onset and duration of each episode.

The investigator will record all reportable events with start dates occurring any time after informed consent is obtained until the last day of study participation. At each study visit, the investigator will inquire about the occurrence of AE/SAEs since the last visit. Events will be followed for outcome information until resolution or stabilization.

9.4 REPORTING PROCEDURES

9.4.1 ADVERSE EVENT RECORDING AND REPORTING

Adverse events, solicited AEs, and SAEs will be assessed at all study visits, documented in the source record, and recorded in the eCRF using accepted medical terms and/or the diagnosis that accurately characterize the event. When the diagnosis is known the AE term recorded in the eCRF will be the diagnosis rather than constellation of symptoms. The SI will assess all AEs for seriousness, relationship to investigational product, severity, and other possible causes.

The timeframe for the collection of AEs and SAEs begins at the first administration of investigational product through to the end of the trial. All adverse events occurring through Visit 4 for all study groups will be collected and recorded in the source document and eCRF.

When an AE has not resolved by the next visit it will be documented in the eCRF as ongoing. Documentation will include date of onset, detailed description of the event and relevant history and physical examination, severity, attribution of the AE, treatment given and date the AE improved or resolved. The medical monitor will review the AEs reported regularly and clarify with SI if there are queries. The data manager will review all AEs for consistency and provide summary of AEs to the medical monitor periodically. Non-clinically significant AEs still ongoing as the end of the study will be listed as continuing. SAEs continuing at the end of the study will be followed to resolution or stabilization. Details of AE reporting are included in the MOP.

The PI, SI, and site staff will exercise due diligence in ascertaining, accurately recording and promptly entering data on the eCRF for all AEs of all study participants. As data becomes available from the participant, the clinic and laboratories, adverse events should be recorded and entered by the site staff on regular basis. Site investigators will review, in a timely manner, the AE source data and determine the severity of the event and relation to the study agent. Site investigators will contact the study medical monitor for consultation of AEs as required.

9.4.2 SERIOUS ADVERSE EVENT REPORTING

The SI will complete a SAE Form within the following time frame:

- All SAEs will be recorded on the SAE Form and submitted by the site PI to the Overall study PI/Sponsor within 48 hours of initial receipt of the information (weekends and holidays are not included) and addressed to:

  Dr. Tarun Saluja

  Study Medical Monitor

  International Vaccine Institute
SNU Research Park, 1 Gwanak-ro, Gwanak-gu,
Seoul, 08826 Republic of Korea,
Phone: +82-2-881-1236
Fax: +82-2-881-1239
Mobile: +82-10-3349-7910
tarun.saluja@ivi.int

- NHRC/DDA to be notified within 2 calendar days by the sponsor/overall study PI/Designee with complete report due within 21 additional calendar days of the first information.

All information (which may include special investigations and treatment received) will be recorded on the SAE Form and submitted to NHRC/DDA. All SAEs will be followed until satisfactory resolution or until the site investigator deems the event to be chronic or to be stable. Other supporting documentation of the event may be requested by the sponsor and should be provided as soon as possible. SAE reporting to IVI IRB is not mandatory and will be reported with the annual renewal report.

- The sponsor will be responsible for notifying the IVI IRB of Suspected Unexpected Serious Adverse Reactions (SUSAR) within 24 hours of initial receipt of the information (weekends and holidays are not included).

The SUSAR report will include the following information:

- It is mandatory to include an identifiable patient, an identifiable reporter, a suspect drug, and an adverse event.
- Protocol information: protocol number and date
- A detailed description of the event, incident, experience, or outcome
- An explanation of the basis for determining that the event, incident, experience, or outcome represents an unexpected problem

9.4.3 SAFETY OVERSIGHT

An independent Data Safety Monitoring Board (DSMB) will be constituted having experts from various fields of Medicine. The DSMB will oversee the study in terms of safety data as per DSMB charter. DSMB will have the authority to halt or terminate the study in case of any safety signals. DSMB chair will issue a recommendation letter after each meeting.

10 STUDY MONITORING

Study monitoring and auditing will be performed in accordance with the sponsor's procedures, ICH E6 (R2) GCP guidelines and any other applicable regulatory requirements.

Upon successful approval of the protocol and establishment of the Regulatory File, the clinical monitor will establish a clinical monitoring plan. To ensure that the investigator and the study staff understand and accept their defined responsibilities, the clinical monitor will maintain

regular correspondence with the site and may be present during the course of the study to verify the acceptability of the facilities, compliance with the investigational plan and relevant regulations, and the maintenance of complete records.

Investigators and/or their study staff will be trained on the study protocol and all applicable study procedures prior to study initiation. Electronic CRFs supplied by the sponsor must be completed for each enrolled participant. The data entries as well as study related documents will be checked by the sponsor and/or trained delegates of the sponsor.

Study progress will be monitored by IVI study team or representative (e.g., a contract research organization) as frequently as necessary to ensure the rights and well-being of study participants are protected; to verify adequate, accurate and complete data collection; protocol compliance and to determine that the study is being conducted in conformance with applicable regulatory requirements. Arrangements for monitoring visits will be made in advance in accordance with the monitoring plan, except in case of emergency.

11 STATISTICAL CONSIDERATIONS

11.1 SAMPLE SIZE

The sample size of the study is calculated to provide about 90% power to show primary objectives of the study and increased to satisfy total safety population of about N=1800 The given sample size of N=450 participants per vaccine group would provide 99% power to detect the non-inferiority of immunogenicity of Vi-DT group (combined group A+B+C) compared to TypbarTCV® (group D) in terms of seroconversion rate. The assumed seroconversion rate of TypbarTCV® is 90% (conservatively assumed from 95-98% in phase 3 trial of Typbar TCV® in India) and the assumed non-inferiority margin is 10% based on WHO TRS 924. One sided test of non-inferiority is used with significance level of 0.0125. This sample size will also provide 97% power for three equivalence tests of GMT ratio of immunogenicity among three lots of Vi-DT (A vs. B, B vs. C and C vs. A) with overall two sided significance level of 0.025. Each of two sided equivalence test have 97% power to show the equivalence of GMT of immunogenicity of Vi-DT with two sided significance level of 0.0085 each. The equivalence margin of ratio was assumed as [0.67, 1.5] based on WHO TRS 924, the true GMT ratio is assumed as 1 and the coefficient of variation on titer of immunogenicity is assumed as 2.0. The CV of GMT of Vi-DT

was assumed conservatively based on phase 1 data. In both of sample size calculation, 15% drop out rate is assumed conservatively considering the potential large variation of experience between sites.

For the additional group,the given sample size of N=360,with 180 participants randomized into 1:1 ratio for Vi-DT+ MMR and MMR alone respectively would provide 80% power to detect the non-inferiority of sero-positive rate of IgG antibody titers for Measles (M), Mumps (M) and Rubella (R) following single dose of MMR at 4 weeks in participants who received MMR + Vi-DT to sero-positive rate in participants who received MMR alone.The assumed seropositive rate of Measles,Mumps and Rubella is 90% and the assumed non-inferiority margin is 10%. One sided test of non-inferiority is used with significance level of 0.025 and 10% drop out rate is considered.

11.2 STATISTICAL ANALYSIS PLAN

The statistical analysis will focus on comparisons of immunogenicity of Vi-DT (Group A,B and C combined) and Typbar TCV® (Group D), and on tests of lot to lot consistency of Vi-DT (comparison among group A, B and C) at 4 weeks post vaccination. Safety of Vi-DT will be assessed by descriptively comparing incidence of common solicited, unsolicited and serious AE between Vi-DT and Typbar TCV® and any incidence of unexpected AE. The interference of Vi-DT to MR vaccination will be assessed descriptively by showing that the immunogenicity of Measles and Rubella concomitantly administer with Vi-DT is similar to the ones when concomitantly administered with Typbar TCV® which already have shown no interference and the ones when administered Vi-DT only. A primary analysis will be performed after all participants complete week 4 visit post Test/Comparator vaccine dose in order to initiate the test vaccine licensure process. Immunogenicity and safety data up to week 4 will be included in this analysis and this will be done in a way so that the study and study personnel remain blinded to the allocation of test/comparator vaccine until the end of the study. The final analysis will be performed when all participants complete week 24 visit. Immunogenicity and safety data up to week 24 will be included in the final analysis

For additional group, the interference of Vi-DT to MMR vaccination will be demonstrated by showing that the immunogenicity of Measles, Mumps and Rubella when administered concomitantly with Vi-DT is non-inferior when administered without Vi-DT (MMR alone).

Additional statistical analysis details will be described in the statistical analysis plan (SAP) (which will be finalized prior to database lock) and any deviation(s) from the original SAP will be described and justified in the final study report.

11.3 STATISTICAL HYPOTHESES

There are two primary objectives to show the non-inferiority of Vi-DT compared to Typbar TCV® and lot to lot consistency of Vi-DT.

The statistical hypotheses for these two primary objectives are:

To demonstrate Non-inferiority of Vi-DT vaccine compared to Typbar TCV®

- Anti-Vi IgG seroconversion rate at wk4 post Vi-DT vaccine (25 µg) (Groups A+B+C) is non-inferior to seroconversion rate at wk4 post Typbar TCV® (Group D) using non-inferiority margin of 10%

To demonstrate the lot-to-lot consistency of Vi-DT vaccine

- Anti-Vi IgG GMT at D28 post each lot of Vi-DT vaccine is equivalent to each other (A vs. B, B vs. C, and C vs. A) using equivalence margin of GMT ratio of [0.67, 1.5]

To preserve the study-wise type 1 error rate 0.05, Bonferroni methods will be utilized to control the multiple testing procedure for two primary objectives. The non-inferiority test of Vi-DT vs. Typbar TCV® will be performed as 0.0125 significance level using one sided test (which is equivalent to significant level of 0.025 using two sided test) and three equivalent tests will be performed as 0.025 overall significant level using two sided test (each of equivalent test will be tested with 0.0083 significant level)

Secondary Comparisons

- Geometric Mean Titers (GMT) of anti-Vi IgG at D28 (4 weeks post vaccination) of Vi-DT (Groups A+B+C) is non-inferior to Typbar TCV® (Group D) using non-inferiority margin of GMT ratio $\geq$ 0.67

- Anti-Vi seroconversion rate at D28 post each lot of Vi-DT vaccine is equivalent to each other (A vs. B, B vs. C, and C vs. A) using equivalence margin of difference of [-10%, 10%]

- Sero-positive rate of IgG antibody titers for Measles (M),Mumps (M) and Rubella (R) following single dose of MMR at 4 weeks in participants who received MMR + Vi-DT is non-inferior to sero-positive rate in participants who received MMR alone with non-inferioirty margin of 10% (additional group only)

The following two secondary endpoints will be described by vaccine groups as well as by strata

- Geometric Mean Titers (GMT) of anti-Vi IgG at D168 (24 weeks post vaccination) of Vi-DT (Groups A+B+C) and Typbar TCV® (Group D)
- Anti-Vi seroconversion rate at D168 post Vi-DT vaccination (25 µg) (Groups A+B+C) and Typbar TCV® (Group D)
- Anti-Vi IgG seroconversion rate and GMT at 4 weeks (day 28) post Vi-DT vaccine (25 µg) (pooled Groups A+B+C) and Typbar TCV® (Group D) in participants who received comcomitantly MR vaccine
- Anti-Vi IgG seroconversion rate and GMT at 4 weeks (day 28) post Vi-DT vaccine (25 µg) (pooled Groups A+B+C) in participants 9-15 months who concomitantly received and did not receive MR vaccine
- Sero-positive rate of IgG antibody titers for Measles (M) and Rubella (R at 4 weeks (day 28) post vaccination of Vi-DT (Groups A+B+C combined) and Typbar TCV® (Group D) in participants who received concomitant MR vaccine
- Anti-Vi IgG seroconversion rate and GMT at 4 weeks (day 28) post Vi-DT vaccine (25 µg) (pooled Groups A+B+C) in participants 9-15 months who concomitantly did not receive MR vaccine and Group E who received MMR

11.4 ANALYSIS DATASETS

The Full Analysis set (FAS) is a modified intention-to-treat (m-ITT) analysis set that will include all participants randomized in the study who received at least one dose of investigational vaccines. This data set will be used for demographic information and safety analysis.

The immunogenicity analysis set is a subset of FAS of those who is randomized, received at least one dose of investigational vaccines and have at least one post-baseline immunogenicity data available.

The per-protocol (PP) analysis set will be a subset of the immunogenicity analysis set who do not have protocol violations (defined as major deviation from the protocol compromising the

scientific integrity of the study) with regards to the inclusion/exclusion criteria, are compliant with study procedures, completed all visits as scheduled and received the correct vaccinations.

The immunogenicity analysis set will be used for the primary analysis of the immunogenicity endpoints. A sensitivity analysis using the PP analysis sets will be conducted for the primary and secondary immunogenicity endpoints.

11.5 DESCRIPTION OF STATISTICAL METHODS

11.5.1 GENERAL APPROACH

This study is a randomized, observer-blinded phase III study in healthy participants with age 6 months to 45 years old at the time of vaccination of investigational vaccine to assess the non-inferiority of immunogenicity of Vi-DT compared to Typbar TCV® and lot to lot consistency among three lots of Vi-DT.

Unless specified as in section 11.3, for non-inferiority test, the significance level is 2.5% with one-sided test and for equivalence test, the significance level is 5% with two sided test.

Analysis of covariance will be used to adjust for baseline titers, stratification and imbalances in baseline characteristics if necessary.

Missing immunogenicity data will not be imputed for the analysis. If missing data is more than 10%, the analysis of missing pattern will be assessed and a multiple imputation technique will be utilized as a sensitivity analysis.

11.5.2 BASELINE DESCRIPTIVE STATISTICS

Demographic characteristics and other baseline data of participants enrolled will be tabulated by vaccine group and overall. Continuous variables such as age, height and weight will be summarized by number of participants, mean, standard deviation, median, minimum and maximum. Categorical variables such as sex will be summarized by frequency and percentage in each vaccine. If a difference in baseline characteristics among groups is suspected, the

statistical significance will be compared using ANOVA for continuous variables, and Chi-square test or Fisher's exact test for categorical variables.

11.5.3 SAFETY ANALYSIS

The following safety endpoints will be descriptively summarized by each group and in each age strata.

- Frequency of local and systemic solicited adverse events during the 7 days after vaccination: the solicited local reactions at the injection site include pain/ tenderness, erythema/redness, swelling/ induration, pruritus associated injection and solicited systemic reactions include fever, headache,fatigue, myalgia,lethargy, irritability, nausea, vomiting, arthralgia, diarrhea, drowsiness, loss of appetite, chills, rash, nasopharyngitis and persistent crying.
- Unsolicited adverse events during 4 weeks after vaccination
- Serious Adverse Events during the entire study period

Number of AEs and proportion of participants with safety endpoints after Vi-DT (Group A, B and C combined) and Typbar TCV® will be summarized and the 95% confidence interval of the proportion will be calculated for Vi-DT and Typbar TCV® within each age strata as well as overall strata. The confidence interval of propotion in case of rate event will be calculated using appropriate methods such Wilson's score or Agresti and Coull.
Occurrence of any SAE during the study and AE that lead participant early drop out of the study listed.

11.5.4 ANALYSIS OF THE PRIMARY IMMUNOGENICITY ENDPOINT(S)

There are two primary endpoints in this study and each of primary endpoint will be tested separately with significance level of 0.025 each using the Bonferroni method.

The first primary immunogenicity endpoint will be measured as seroconversion rate of Vi-DT (Group A, B and C combined) and Typbar TCV® after 4 week of vaccination for non-inferiority comparison.

For assessment of seroconversion rate, the proportion of participants with at least 4-fold rise anti-Vi IgG ELISA antibody titer at 4 weeks as compared to prior to the investigational product dosing (Day 0) will be calculated. The primary analysis of the primary endpoint will be done by the generalized linear model for binomial distribution with group and strata as covariates.
The two-sided 97.5% CI of sero-conversion rate for each vaccine group and their difference will be provided with an estimate and non-inferiority of Vi-DT will be confirmed if the lower limit of two tailed 97.5% confidence interval of the difference of seroconversion rate of Vi-DT and Typbar TCV is greater than the non-inferiority margin of -10%.

The second primary endpoint is geometric mean titer (GMT) of anti-Vi IgG of Vi-DT after 4 weeks of vaccination to assess the lot to lot consistency of Vi-DT.

To assess the lot to lot consistency, three equivalence tests (Group A vs. B, B vs. C and A vs. C) on GMT of Vi-DT at 4 weeks will be performed with significance level of 0.0083 each (overall significance level of 0.025 for lot-to-lot consistency).The equivalence of anti-Vi GMT at 4 weeks post vaccination of Vi-DT between two different lots will be analyzed using an analysis of covariance model with group and strata as covariates after log transformation. The equivalence of two lots will be confirmed if the both limits of two-tailed 99.17% confidence interval of the ratio of GMT between two lots of Vi-DT is within the equivalence margin of [0.67, 1.5].

11.5.5 ANALYSIS OF THE SECONDARY IMMUNOGENICITY ENDPOINT(S)

The first secondary endpoint is GMT at 4 weeks in the Vi-DT (Group A, B and C combined) compared to Typbar TCV (Group D). The non-inferiority of anti-Vi GMT at 4 weeks post vaccination of Vi-DT compared to anti-Vi GMT at 4 weeks of Typbar TCV® vaccine will be analyzed using an analysis of covariance model with group and strata as covariates after log transformation. The non-inferiority of Vi-DT will be confirmed if the lower limit of two-tailed 95% confidence interval of the ratio of GMT of Vi-DT to TypbarTCV® is greater than the non-inferiority margin of 0.67.

The seroconversion rate and anti-VI GMT of Vi-DT (Group A, B and C combined) and Typbar TCV® (Group D) at week 24 will be summarized descriptively by vaccine group as well as by strata.

The equivalence of anti-Vi seroconversion at 4 weeks post vaccination of Vi-DT between two different lots will be analyzed using the generalized linear model for binomial distribution with group and strata as covariates. The equivalence of two lots will be confirmed if both limits of two-tailed 98.3% confidence interval of the difference of seroconvergence between two lots of Vi-DT is within the equivalence margin of [-10%, 10%].

For participants who received MR vaccine concomitantly with Vi-DT or Typbar TCV® at 0 week, immunogenicity (seropositive rate, GMT) at 4 weeks will be summarized for Vi-DT (3 lots combined) vs. Typbar TCV® to assess the non-interference of Vi-DT on measles, and rubella vaccine

For participants who received Vi-DT concomitantly with or without MR vaccine, immunogenicity (seropositive rate, GMT) at 4 weeks will be summarized to assess the non-interference of Vi-DT + MR vs Vi-DT alone.

Anti-Vi IgG seroconversion rate and GMT at 4 weeks (day 28) post Vi-DT vaccine (25 μg) (pooled Groups A+B+C) in participants 9-15 months who concomitantly did not receive MR vaccine and Group E who received MMR will be summarized to assess the non-interference of concomitant MMR administration on Immunogenecity of ViDT.

In additional group for participants who received MMR vaccine concomitantly with or without Vi-DT at 0 week, the seropositive rate of anti-measles,anti-mumps and anti-rubella antibody titers at 4 weeks will be analyzed. The non-inferiority of Vi-DT + MMR group compared to MMR alone group will be confirmed if the lower limit of one tailed 97.5% confidence interval of their difference of seropositive rates is greater than the non-inferiority margin of -10%.

11.5.6 ADHERENCE AND RETENTION ANALYSES

Summaries of Participants Disposition will be based on full analysis set (FAS). A flow diagram of participant disposition (CONSORT flow diagram) will illustrate the progress of participants through the study duration from initial screening for eligibility to the completion of the final primary outcome assessment. Number and percentage by vaccine group will be given for participants in the FAS, immunogenicity analysis set and PP analysis sets, and reasons for study discontinuation.

11.5.7 PLANNED INTERIM ANALYSIS

There is no planned interim analysis.

11.5.8 ADDITIONAL SUB-GROUP ANALYSIS

The analyses of primary and secondary immunogenicity analyses will be repeated within each age strata. Potential difference in safety and immunogenicity by sex, age groups may be investigated.

11.5.9 MULTIPLE COMPARISON/MULTIPLICITY

To keep the overall study-wise significant level of 0.05, two primary endpoints will be tested with significant level of 0.025 each using Bonferroni approach. The 2nd primary endpoint for lot to lot consistency includes three equivalence tests where 0.0083 significant level is used in each test using Bonferroni approach to keep the overall significant level of 0.025.

11.5.10 EXPLORATORY ANALYSES

The number of laboratory-confirmed typhoid cases occurring within 24 weeks of follow-up period after 4 weeks post vaccination will be summarized by Vi-DT (Groups A, B and C combined) and Typbar TCV® (Group D).

12 SOURCE DOCUMENTS AND ACCESS TO SOURCE DOCUMENTS

Data recorded on the electronic Case Report Forms (eCRF) will be verified by checking the eCRF entries against source documents (i.e., all original records, laboratory reports, medical records, diary cards, memory aids, etc…) in order to ensure data completeness and accuracy as required by study protocol. Source documents will be stored at the clinical site in a secured place under lock and key. The investigator and/or site staff must make eCRFs and source

documents of participants enrolled in this study available for inspection by IVI clinical team, clinical research associate (CRA) or its representative at the time of each monitoring visit.

At a minimum, source documentation must be available to substantiate participant identification, eligibility and participation, proper informed consent procedures, dates of visits, adherence to protocol procedures, adequate reporting and follow-up of adverse events, administration of concomitant medication, study vaccine receipt/dispensing/return records, study vaccine administration information, and date of completion and reason. Specific items required as source documents will be reviewed with the investigator before the study.

The source documents must also be available for inspection, verification and copying, as required by regulations, by officials of the regulatory health authorities (e.g., NHRC, DDA, others) and/or site IRBs and for possible audit by IVI Quality Management, Regulatory agency, notified body and collaboratorys/donors (e.g., Bill and Malinda Gates Foundation). The investigator and study site staff must comply with applicable privacy, data protection and medical confidentiality laws for use and disclosure of information related to the study and enrolled participants.

The participant must also allow access to medical records. Each participant should be informed of this prior to the start of the study by administration of the inform consent per ICH E6 (R2).

Each participant will have a complete source documentation of records including study log books, ICF, lab reports and test results for the entire study period. Appropriate source documents will be prepared by study staffs. These records must be available to the IVI and regulatory authorities upon request for review.

13 DATA HANDLING AND RECORD KEEPING

13.1 DATA COLLECTION AND MANAGEMENT RESPONSIBILITIES

Electronic Case Report Forms (eCRF) will be used for recording data for each participant enrolled in the study. The site investigators are responsible to ensure the accuracy, completeness, legibility and timeliness of the data captured in eCRF. Data captured in the eCRF derived from source documents will be consistent with the source documents. In case of

discrepancies, data will be clarified and corrected. The IVI will provide guidance to investigator on making corrections to the eCRF.

Study staff will extract all data collected in source documents and work books for computerization into the eCRF. Data will be entered into computer in a dedicated area located using data entry programs specially created for the study.. Edit checks will be programmed in the eCRF to identify data entry errors during transcription, including range and consistency checks wherever applicable.

This data management system will provide error reports and summary reports for each activity. The data will be automatically backed up systematically onto Cloud server. In addition, the software will provide for an audit trail of all sequential changes made. Data security for this data management system will be augmented by automatic computer virus scanning at start-up of data entry and data management session, and password protection for accessing data and data management software. Data entry and cleaning will be conducted at the sites. Final data cleaning, data freezing and data analysis will be performed at the IVI. Unblinding of study vaccines will be carried out after database lock. All data should be stored in a secure data base (i.e., either in a vetted cloud network or controlled server environment with controlled access (e.g., only IT personnel).

13.2 STUDY RECORDS RETENTION

The site Investigators (SI) will retain all study records required by sponsor and by the applicable regulations in a secure and safe facility. The SI will consult IVI representative before disposal of any study records, and will notify the sponsor of any change in the location, disposition, or custody of the study files. These documents should be retained for at least 3 year after the approval of a marketing application or after discontinuation of clinical development. (ICH E6 (R2), 4.9.5). The IVI will inform the SI as to when these documents no longer need to be retained (ICH E6 (R2), 5.5.12).

13.3 PUBLICATION AND DATA SHARING POLICY

IVI assures that the key design elements of this protocol will be posted in a publicly accessible database such as Clinicaltrials.gov. All data collected during this study will be used to support

this vaccine development plan until licensure and WHO prequalification. All individual data will stay strictly confidential. Analyzed data may be presented in scientific conferences, and published in peer-reviewed scientific journals. IVI reserve the right for overall study results publication. Any abstract, presentation and manuscript must be shared sufficiently in advance for proper review and approval as per IVI procedures. Anyone wishing to publish or present site-specific data obtained during and/or after completion of the study will conform to study site and IVI data sharing policies and then forward the publication and/or presentation for review and approval by IVI.

14 QUALITY ASSURANCE AND QUALITY CONTROL

Quality Assurance (QA) oversight will be required at all stages of the trial process per ICH E6 (R2) section 5.0.

Quality Control (QC) procedures will be implemented beginning with the data entry system and data QC checks that will be run on the database will be generated. Any missing data or data anomalies will be communicated to the site(s) for clarification/resolution.

During study conduct, the Sponsor or its designee will conduct periodic monitoring visits (i.e., QC checks) to ensure that the protocol, ICH E6 (R2) Good Clinical Practice, local regulatory requirements and sponsor's controlled documents (e.g., Standard Operating Procedure) are being followed. The monitors will review source documents to confirm that the data recorded on CRFs is accurate.

In addition to on-going QA oversight, selected investigator sites will be subjected to quality assurance audits performed by the sponsor or its designee, and/or by regulatory authorities and/or notified bodies.

The investigational sites will provide direct access to all study related sites, source data/documents, and reports for the purpose of monitoring and auditing by the sponsor, and inspection by local and regulatory authorities and/or notified bodies.

15 ETHICS/PROTECTION OF HUMAN PARTICIPANTS

15.1 REGULATORY AND ETHICAL COMPLIANCE

The investigators will ensure that this study is conducted in full conformity with the ICH E6 (R2) and E11 GCP Guidelines, Council for International Organizations of Medical Science (CIOMS), local country's ethical policy statement or the Declaration of Helsinki, whichever provides the most protection to human participants.

15.2 PARTICIPANT AND DATA CONFIDENTIALITY

Participant confidentiality is strictly held in trust by the participating investigators, their staff, and the sponsor and their agents. This confidentiality is extended to cover testing of biological samples and genetic tests in addition to the clinical information relating to participants. Therefore, the study protocol, documentation, data, and all other information generated will be held in strict confidence. No information concerning the study or the data will be released to any unauthorized third party without prior written approval of the sponsor.

The study monitor, other authorized representatives of the sponsor, representatives of the IRB or pharmaceutical company supplying study product may inspect all documents and records required to be maintained by the investigator, including but not limited to, medical records (office, clinic, or hospital) and pharmacy records for the participants in this study. The clinical study site will permit access to such records.

The study participant's contact information will be securely stored at clinical site for internal use during the study. At the end of the study, all records will continue to be kept in a secure location for as long a period as dictated by local IRB and local regulations.

Individual participants and their research data will be identified by a unique study identification number. The study data entry and study management systems used by clinical site and by IVI Data Management will be secured and password-protected.

No personal identifier will be used in any publication or communication used to support this research study. The participant's identification number will be used in the event it becomes necessary to identify data specific to a single participant.

15.3. RESEARCH USE OF STORED HUMAN SAMPLES

- Intended Use: Samples and data collected under this protocol may be used to study immune responses to the vaccines administered and for safety purpose if deemed necessary per medical judgement of the SI or special request from the sponsor or IRBs. No genetic testing will be performed.
- Storage: Samples and data will be stored at respective sites and IVI using codes assigned by the investigators. Data will be kept in password-protected computers. Only investigators will have access to the stored samples and data.
- Disposition at the completion of the study: All stored samples will be sent to IVI and be stored as per informed consent for Research of Human Derivatives.
- Study participants who request destruction of samples will be notified of compliance with such request and all supporting details will be maintained for tracking.

15.4 FUTURE USE OF STORED SPECIMENS

With the participant's approval (consent form) and as approved by NHRC, local sites and IVI IRBs, the identified biological samples will be stored at the immunology lab in IVI for future use. The immunology lab at IVI will be attributed a code that will allow linking the biological specimens with the phenotypic data from each participant, maintaining the masking of the identity of the participant.

During the conduct of the study, an individual participant can choose to withdraw consent to have biological specimens stored for future research. However, withdrawal of consent with regard to bio sample storage will not be possible after the study is completed.

The stored samples may be used for additional assessment of immunogenicity, study of possible immune correlates of protection, validation of assays, testing of new assays, and for safety purpose.

16 REFERENCES

1. Background Paper on Typhoid Vaccines for SAGE Meeting (October 2017).
2. John J, Van Aart CJ, Grassly NC (2016) The Burden of Typhoid and Paratyphoid in India: Systematic Review and Meta-analysis. PLoS Negl Trop Dis 10: e0004616.
3. Buckle GC, Walker CL, Black RE (2012) Typhoid fever and paratyphoid fever: Systematic review to estimate global morbidity and mortality for 2010. J Glob Health 2: 010401.
4. Crump JA, Luby SP, Mintz ED (2004) The global burden of typhoid fever. Bull World Health Organ 82: 346-353.
5. Mogasale V, Maskery B, Ochiai RL, Lee JS, Mogasale VV, et al. (2014) Burden of typhoid fever in low-income and middle-income countries: a systematic, literature-based update with risk-factor adjustment. Lancet Glob Health 2: e570-580.
6. Marks F, von Kalckreuth V, Aaby P, Adu-Sarkodie Y, El Tayeb MA, et al. (2017) Incidence of invasive salmonella disease in sub-Saharan Africa: a multicentre population-based surveillance study. Lancet Glob Health 5: e310-e323.
7. Karkey, A., et al., Kathmandu, Nepal: still an enteric fever capital of the world. J Infect Dev Ctries, 2008. 2(6): p. 461-5.
8. Maskey AP, Basnyat B, Thwaites GE, Campbell JI, Farrar JJ, et al. (2008) Emerging trends in enteric fever in Nepal: 9124 cases confirmed by blood culture 1993–2003. Trans R Soc Trop Med Hyg 102: 91–95.
9. Karkey A, Arjyal A, Anders KL, Boni MF, Dongol S, et al. (2010) The burden and characteristics of enteric fever at a healthcare facility in a densely populated area of Kathmandu. PLoS One 5: e13988
10. McClelland M, Sanderson KE, Spieth J, Clifton SW, Latreille P, et al. (2001) Complete genome sequence of Salmonella enterica serovar Typhimurium LT2. Nature 413: 852-856.
11. Crump JA, Luby SP, Mintz ED (2004) The global burden of typhoid fever. Bull World Health Organ 82: 346-353.
12. Bhan MK, Bahl R, Bhatnagar S (2005) Typhoid and paratyphoid fever. Lancet 366: 749-762.
13. Hornick RB, Greisman SE, Woodward TE, DuPont HL, Dawkins AT, et al. (1970) Typhoid fever: pathogenesis and immunologic control. 2. N Engl J Med 283: 739-746.
14. Hornick RB, Greisman SE, Woodward TE, DuPont HL, Dawkins AT, et al. (1970) Typhoid fever: pathogenesis and immunologic control. N Engl J Med 283: 686-691.
15. DeRoeck D, Jodar L, Clemens J (2007) Putting typhoid vaccination on the global health agenda. N Engl J Med 357: 1069-1071.

16. Levine MM, Ferreccio C, Black RE, Germanier R (1987) Large-scale field trial of Ty21a live oral typhoid vaccine in enteric-coated capsule formulation. Lancet 1: 1049-1052.

17. Klugman KP, Koornhof HJ, Robbins JB, Le Cam NN (1996) Immunogenicity, efficacy and serological correlate of protection of Salmonella typhi Vi capsular polysaccharide vaccine three years after immunization. Vaccine 14: 435-438.

18. Szu SC TD, Trofa AC, et al. (1994) Laboratory and preliminary clinical characterization of Vi capsular polysaccharide-protein conjugate vaccines. Infection and Immunity 62:4440-4.

19. Thiem VD, Lin FY, Canh DG, Son NH, Anh DD, et al. (2011) The Vi conjugate typhoid vaccine is safe, elicits protective levels of IgG anti-Vi, and is compatible with routine infant vaccines. Clin Vaccine Immunol 18: 730-735.

20. Micoli F, Rondini S, Pisoni I, et al. (2011) Vi-CRM 197 as a new conjugate vaccine against Salmonella Typhi. Vaccine 29:712-20.

21. van Damme P KF, Anemona A, et al (2011) Safety, immunogenicity and dose ranging of a new Vi-CRM(1)(9)(7) conjugate vaccine against typhoid fever: randomized clinical testing in healthy adults. PLos One 6:e25398.

22. Mitra M, Shah N, Gosh A, et al. (2016) Efficacy and safety of vi-tetanus toxoid conjugated typhoid vaccine (PedaTyph™) in Indian children: School based cluster randomized study. Human Vaccin Immunother 12:939-45.

23. Mohan VK, Varanasi V, Singh A, Pasetti MF, Levine MM, et al. (2015) Safety and immunogenicity of a Vi polysaccharide-tetanus toxoid conjugate vaccine (Typbar-TCV) in healthy infants, children, and adults in typhoid endemic areas: a multicenter, 2-cohort, open-label, double-blind, randomized controlled phase 3 study. Clin Infect Dis 61: 393-402.

24. Maria Rosario Capeding, Samuel Teshome, Tarun Saluja, Khalid Ali Syed, Deok Ryun Kim et al. Safety and Immunogenicity of a Vi-DT Typhoid Conjugate Vaccine: Phase I Trial in Healthy Filipino Adults and Children.Vaccine 36 (2018),3794-3801

25. Cadoz M (1998) Potential and limitations of polysaccharide vaccines in infancy. Vaccine 16:1391-5.

26. Kanungo S, Dutta S, Sur D (2008) Epidemiology of typhoid and paratyphoid fever in India. J Infect Dev Ctries 2:454-60.

27. WHO. Typhoid vaccines: WHO position paper March 2018,93,153–172.

28. Engels EA, Lau J. Vaccines for preventing typhoid fever. The Cochrane database of systematic reviews. 2000 (2):Cd001261. doi: 10.1002/14651858.Cd001261. PubMed PMID: 10796623.

29. Bhutta ZA, Capeding MR, Bavdekar A, Marchetti E, Ariff S, et al. (2014) Immunogenicity and safety of the Vi-CRM197 conjugate vaccine against typhoid fever in adults, children, and infants in south and southeast Asia: results from two randomised, observer-blind, age de-escalation, phase 2 trials. Lancet Infect Dis 14: 119-129.

30. Canh DG, Lin FY, Thiem VD, Trach DD, Trong ND, et al. (2004) Effect of dosage on immunogenicity of a Vi conjugate vaccine injected twice into 2- to 5-year-old Vietnamese children. Infect Immun 72: 6586-6588.

31. http://www.hsa.gov.sg/content/dam/HSA/HPRG/Clinical_Trials/Guidance/HSA_CTB_Guidance_Labelling_MP_TP_2May2017.pdf

32. Division of AIDS/NIAID/NIH (November 2014) Table for Grading the severity of Adult and Pediatric Adverse Events. Version 2.0.

33. 백신임상시험 이상반응 중증도 평가 가이드라인_11-1470000-002625-14_DEC2011

17 APPENDICES

a. Statement of compliance

b. Vi-DT Phase II result summary
